# A collection of forcefield precursors for metal–organic frameworks†

**DOI:** 10.1039/c9ra07327b

**Published:** 2019-11-13

**Authors:** Taoyi Chen, Thomas A. Manz

**Affiliations:** Department of Chemical & Materials Engineering, New Mexico State University Las Cruces New Mexico 88003-8001 USA tmanz@nmsu.edu

## Abstract

A host of important performance properties for metal–organic frameworks (MOFs) and other complex materials can be calculated by modeling statistical ensembles. The principle challenge is to develop accurate and computationally efficient interaction models for these simulations. Two major approaches are (i) *ab initio* molecular dynamics in which the interaction model is provided by an exchange–correlation theory (*e.g.*, DFT + dispersion functional) and (ii) molecular mechanics in which the interaction model is a parameterized classical force field. The first approach requires further development to improve computational speed. The second approach requires further development to automate accurate forcefield parameterization. Because of the extreme chemical diversity across thousands of MOF structures, this problem is still mostly unsolved today. For example, here we show structures in the 2014 CoRE MOF database contain more than 8 thousand different atom types based on first and second neighbors. Our results showed that atom types based on both first and second neighbors adequately capture the chemical environment, but atom types based on only first neighbors do not. For 3056 MOFs, we used density functional theory (DFT) followed by DDEC6 atomic population analysis to extract a host of important forcefield precursors: partial atomic charges; atom-in-material (AIM) C_6_, C_8_, and C_10_ dispersion coefficients; AIM dipole and quadrupole moments; various AIM polarizabilities; quantum Drude oscillator parameters; AIM electron cloud parameters; *etc.* Electrostatic parameters were validated through comparisons to the DFT-computed electrostatic potential. These forcefield precursors should find widespread applications to developing MOF force fields.

## Abstract 2

Atom-in-material (AIM) partial charges, dipoles and quadrupoles, dispersion coefficients (C_6_, C_8_, C_10_), polarizabilities, electron cloud parameters, radial moments, and atom types were extracted from quantum chemistry calculations for >3000 MOFs.

## Graphical abstract

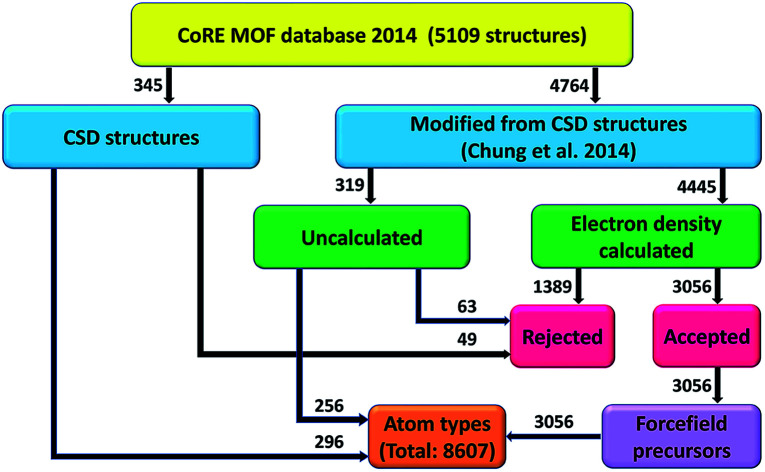

## 1. Introduction

Metal–organic frameworks (MOFs) are a kind of coordination network comprised of metal atoms connected by organic linkers.^1^ In this work, we are interested in MOFs that are porous crystals. Because of their nanoporous structures, these materials attract much interest for gas storage, gas separation, catalysis, and other applications.^2–6^ Many thousands of MOF crystal structures have been deposited in the Cambridge Structural Database (CSD).^1,7,8,115–117^ Most often, these crystal structures were measured using X-ray diffraction crystallography (XRDC). Since hydrogen atoms contain no core electrons, they diffract X-rays extremely weakly.^9^ This makes it much harder to refine hydrogen atom positions than positions for heavier atoms.^9–11^ Consequently, hydrogen atom positions may be unresolved in some of the reported crystal structures. Other complications include the presence of disordered atoms, solvent molecules, and/or free ions in some of the reported MOF crystal structures.

**Fig. 1:**
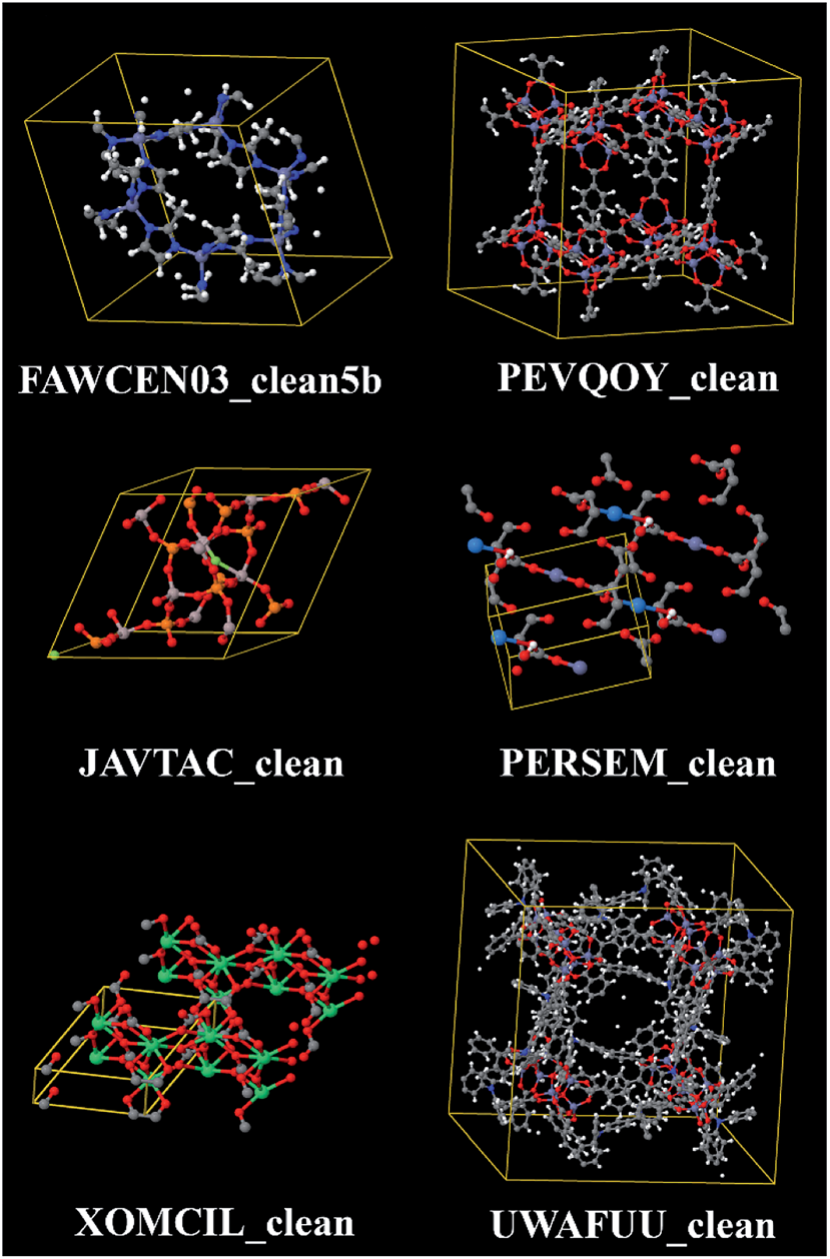
Example structures from the 2014 CoRE MOF database. Top left: ZIF-8 is a zeolitic imidazolate framework containing zinc atoms and imidazolate linkers. Top right: IRMOF-3-AM4XL is an isoreticular metal–organic framework (IRMOF) containing interligand crosslinks.^51^ Middle left: example metal–inorganic framework (MIF) containing no carbon or hydrogen atoms. Middle right: example MOF containing an actinide element (uranium). Bottom left: example MOF having a small unit cell, containing a lanthanide element (erbium), with no hydrogen atoms. Bottom right: example MOF having a large unit cell (1160 atoms).

In 2014, Chung *et al.* reported a Computation Ready Experimental (CoRE) MOF database that was constructed by first searching the CSD to identify MOFs and then partially cleaning these structures.^7^ The searching step was designed to identify structures containing metal atoms bonded to non-metal atoms that form 3-dimensional networks. The cleaning process was intended to fix or discard structures containing disordered atoms and partial occupancies. The cleaning process was also intended to remove solvent molecules and other small adsorbates in the MOF's pores but to retain charge-balancing ions. Finally, missing hydrogen atoms were added to some of the structures. However, this cleaning process was imperfect resulting in some structures with errors.^12,13,115^ This 2014 CoRE MOF database was the starting point for our study. It contains a total of 5109 structures, of which 4764 structures were modified during the cleaning process and 345 retained their original CSD structures.^7^Fig. 1 illustrates some of the chemical diversity within this database. Since organic compounds contain carbon, porous metal-linker networks that do not contain any carbon atoms are called metal–inorganic frameworks (MIFs).^14,15^ The 2014 CoRE database contains some structures that are MIFs.^13^

Several follow-ups were subsequently made to the CoRE MOF database. In 2016, Nazarian *et al.* reported DDEC net atomic charges (NACs) for 2932 structures.^16^ In 2017, Nazarian *et al.* reported DFT-optimized geometries for 838 structures including 502 with computed DDEC NACs.^17^ Soon, a major revision of the CoRE MOF database will be published that expands the number of structures to approximately 15 thousand (Yongchul G. Chung, Emmanuel Haldoupis, Benjamin J. Bucior, Maciej Haranczyk, Seulchan Lee, Hongda Zhang, Konstantinos D. Vogiatzis, Marija Milisavljevic, Sanliang Ling, Jeff S. Camp, Ben Slater, J. Ilja Siepmann, David S. Sholl, Randall Q. Snurr, in revision). Altintas *et al.*^115^ reported a comparative analysis between the 2014 CoRE MOF database and the CSD-derived MOF database of Moghadam *et al.*^116^

Although many thousands of MOFs have been synthesized to date, this is only a tiny fraction of the number of MOFs that could potentially be made.^1^ Owing to the large number of different metal atoms and organic linkers that could be combined in various ways, there is a nearly infinite number of potentially synthesizable MOFs.^1,18–20^ Computational chemistry can be an efficient way to search this vast chemical space to help identify the most promising materials to later synthesize and experimentally test.^21^ This will avoid unnecessary efforts to synthesize a large number of materials that do not perform well for the desired applications.

A host of important performance properties for MOFs and other complex materials can be calculated by modeling statistical ensembles (*e.g.*, constant NVE, NVT, NPT, μVT, μPT, NPH, *etc.*). Here, the properties can be roughly divided into thermodynamic properties that occur at equilibrium and dynamic properties that describe transient system response to non-equilibrium. Gas adsorption isotherms are often computed in the grand canonical (μVT) or Gibbs ensembles.^22,23^ Gas diffusion constants are often computed in the canonical (NVT) or microcanonical (NVE) ensembles using equilibrium molecular dynamics.^24,25^ System behaviors under extreme conditions, such as high pressures, low or high temperatures, applied electromagnetic fields, irradiation, high stresses, and corrosive conditions are of emerging interest.

When modeling statistical ensembles, an interaction model is required to compute the system's energy as a function of chemical configuration. Several different types of interaction models exist depending on the length scale at which the system is modeled. The smallest length scale considers individual electrons using an exchange–correlation theory (*e.g.*, DFT + dispersion functional^26,27^) to solve the Schrodinger equation for the material's electron distribution (*i.e.*, quantum chemistry). A somewhat larger length scale considers individual atoms interacting *via* a classical force field in atomistic simulations.^21,28,29^ Coarse-grained and continuum models treat even larger length scales.^30,31^

Quantum chemistry using an exchange–correlation theory has different advantages and limitations compared to classical atomistic simulations using a force field. An exact exchange–correlation theory (*e.g.*, full configuration interaction) would describe all material types but is extremely computationally expensive. In practice, approximate exchange–correlation theories are used that describe an extremely broad range of different material types with acceptable accuracy and computational efficiency. Because exchange–correlation theories are not material specific, they should not have to be reparameterized for a new material type. In contrast, classical force fields have to be extensively parameterized for individual atoms or atom types.^29,32^ Because computing the system's energy is usually much faster using a classical force field than an exchange–correlation theory, classical atomistic simulations can usually be performed faster and over larger length and time scales than quantum chemistry calculations.

Classical force fields typically contain bonded and non-bonded terms. Bonded terms describe flexibility (bond stretching, angle bending, torsion, and/or out-of-plane parameters) and have been incorporated into many flexible force fields for MOFs.^33–38^ Non-bonded terms describe electrostatic interactions (NACs, atomic multipoles, charge penetration, polarizability^39–43^), London dispersion interactions, and/or short-range exchange-repulsion.^44,45^ When optimizing the bonded parameter values, it is orders of magnitude more efficient to fit them to atom-in-material forces across multiple geometries than to only fit them to the system's total energy across multiple geometries.^46,47^ Because each geometry yields many atom-in-material forces but only one total energy, fewer geometries are needed to fit the bonded parameter values to forces than to energies.

**Fig. 2:**
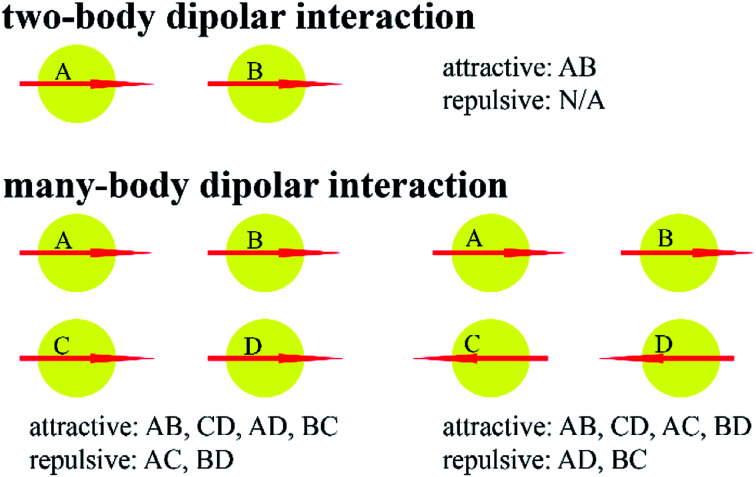
Two-body (top) *versus* many-body (bottom) dipolar interactions. Two-body dipolar interactions can be purely attractive. As shown in the bottom left and bottom right, many-body dipolar interactions often contain a mixture of attractive and repulsive contributions.

A classical force field must contain many-body dispersion and/or many-body polarization to accurately describe system properties over a wide range of conditions.^48^ As shown in Fig. 2, dipolar interactions between two particles can be purely attractive, but dipolar interactions between many non-collinear particles are partly attractive and partly repulsive. Consequently, fitting two-body potentials (*e.g.*, Lennard-Jones) to two-particle interaction curves gives force fields that often overestimate liquid-phase densities.^49^ If the parameters of the two-body potential are adjusted to yield correct liquid-phase densities, then the two-particle interaction curve will be incorrectly described.^45^ This problem can be fixed by explicitly including many-body dispersion and/or many-body polarization in the force field. For example, Kiss and Baranyai showed polarizability must be included in a force field to describe liquid water accurately over a wide range of conditions.^50^

Currently, there is a bottleneck to effectively model statistical ensembles: the quantum chemistry calculations are computationally expensive while the classical force fields are tedious to accurately parameterize. To resolve this bottleneck, either the quantum chemistry calculations should be sped up by orders of magnitude or classical force field parameterization should be made orders of magnitude easier. Some progress was made, but much more work remains to be done. Car-Parrinello molecular dynamics (CPMD) is a quantum chemistry method in which the wavefunction does not have to be self-consistently solved at each time step, because it evolves *via* an effective Lagrangian. CPMD improves the computational efficiency of *ab initio* molecular dynamics, but CPMD is limited to materials containing a band gap (*i.e.*, insulators and semi-conductors).^52–54^ VASP can perform *ab initio* molecular dynamics even for metals, but this requires computing self-consistent orbitals at each time step.^54,55^ Recently, many studies focused on first-principles derived classical force fields whose parameters are extracted from quantum chemistry calculations.^22,35,40,44,45,49,56,57^ Other studies parameterized force fields by fitting to experimental data^36,58^ or using machine learning (ref. 59–62).

**Fig. 3:**
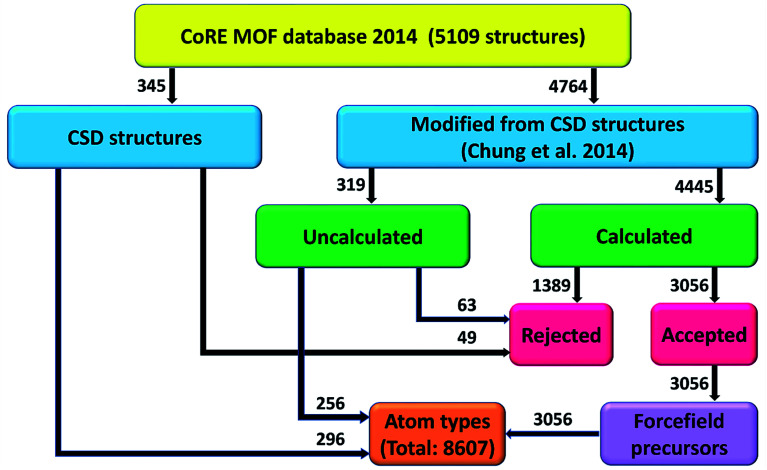
Project flow diagram. Number of structures labeled next to each arrow.

This work is a large-scale computation of atom types and non-bonded parameters for MOFs. Fig. 3 summarizes our project workflow. The 5109 structures from the 2014 CoRE MOF database included 345 structures directly from CSD plus 4764 structures modified by Chung *et al.*^7^ We attempted DFT calculations on all of the modified structures except the largest ones containing >∼1700 atoms; however, some DFT calculations did not converge. DFT calculations converged for 4445 structures that formed the calculated group, while the unconverged and large structures comprised 319 uncalculated structures. The acceptance or rejection criteria described in Section 2.4 were then applied to the CSD, calculated, and uncalculated structures. Atom typing was then applied to all of the accepted structures. A total of 8607 different atom types were identified. Various forcefield precursors were computed and reported in the ESI† for 3056 accepted calculated structures.

## 2. Methods

### 2.1 Electron density calculation

The periodic quantum chemistry calculations of MOFs were performed using the PBE^63^ exchange–correlation functional and the VASP^64,65^ software. Frozen-core all-electron calculations were performed using the projector augmented wave^66,67^ (PAW) method that uses a scalar-relativistic treatment of relaxed valence electrons and high-level relativistic treatment of frozen-core electrons.^68^ The PAW potentials recommended on the VASP website were used for all these calculations. The planewave energy cutoff was 400 eV. Following prior recommendation,^69^ the number of k-points along each lattice vector times the lattice vector length was ≥16 Å. If the *k*-points mesh was 1 × 1 × 1 then the Gamma point was used, otherwise we used Monkhorst–Pack^70^*k*-point grids. Real-space grids were chosen to avoid aliasing errors (*i.e.*, PREC = accurate).^69^ The magnetic alignment of unpaired electron spins was optimized starting from the VASP default guess (which corresponds to a ferromagnetic alignment).

Calculations for the small organic molecules in Section 3.3 were performed using Gaussian16 (ref. 71) with the B3LYP^72,73^ functional and def2QZVPPDD^74,75^ basis set. Geometries of these molecules were optimized at this level of theory.

### 2.2 Computing forcefield precursors *via* atoms-in-material partitioning

Table 1 lists the computed forcefield precursors. The net atomic charge, atomic dipole, atomic quadrupole, electron cloud parameters, 〈*r*^3^〉 and 〈*r*^4^〉 radial moments, and atomic spin moment were computed in the Chargemol program using DDEC6 partitioning.^69,76,77^

**Table 1:** List of computed forcefield precursors

Forcefield precursor	Method reference
Net atomic charge	69 and 76
Atomic dipole and quadrupole	76
Electron cloud parameters	This work
〈*r*^3^〉 and 〈*r*^4^〉 radial moments	69
Atomic spin moment	69 and 77
C_6_, C_8_, C_10_ dispersion coefficients	74
QDO parameters	74
Forcefield, fluctuating, and static polarizability scalars	74
Static polarizability tensor	74

The electron cloud parameters fit the electron density tail of each atom in the material to an exponential decay function. Specifically, the logarithm of the spherically averaged electron density assigned to atom A was fit to a straight line

1 ln(*ρ*^avg^_A_(*r*_A_)) ≈ *a* − *br*_A_

using least squares regression over the *r*_A_ values for which 10^−4^ ≤ *ρ*^avg^_A_(*r*_A_) ≤ 10^−2^ e bohr^−3^. *r*_A_ is the distance from position *r⃑* to atom A's nuclear position. The fitted intercept *a*, slope *b*, and squared correlation coefficient *R*^2^ are reported. The *R*^2^ values were nearly always > 0.99 indicating nearly perfect linearity. These electron cloud parameters have several uses in molecular mechanics force fields. First, they are useful to describe charge penetration (also called cloud penetration).^76^ Second, they are useful to describe short-range exchange-repulsion.^44^ van Vleet *et al.* showed the Born-Mayer exponential term describing short-range exchange-repulsion has an effective exponent of ∼0.84 × *b*.^44^ Although their results were derived for iterated stockholder atom^78^ (ISA) partitioning,^44^ we expect the same relation to hold for DDEC6 partitioning. Third, these electron cloud parameters are useful to parameterize dispersion damping functions (*e.g.*, Tang–Toennies^79,80^) that keep the dispersion energy finite as the distance between two atoms approaches zero.^44,45^

The polarizabilities, dispersion coefficients, and quantum Drude oscillator (QDO) parameters were computed using the MCLF method^74^ and software (ref. 81). The MCLF method yields several different types of polarizabilities and dispersion coefficients. The forcefield polarizability is a non-directionally screened polarizability that is suitable for use as an input parameter in polarizable force fields.^74^ This avoids double counting the directional screening, because directional screening naturally arises during the classical atomistic simulation when the force field is used.^74^ The fluctuating polarizability is the polarizability associated with the fluctuating dipoles of the London dispersion interaction.^74^ The static polarizability and static polarizability tensor quantify the system's response to an externally applied constant electric field.^74^ Various dispersion coefficients are associated with different kinds of fluctuating multipoles: C_6_ (dipole–dipole), C_8_ (dipole–quadrupole), C_9_ (dipole–dipole–dipole), C_10_ (dipole–octupole and quadrupole–quadrupole).^74,82,83^ The C_9_ coefficients were not printed to the forcefield precursors file, because they can be readily computed^74^ from the printed forcefield polarizability and QDO parameters. The MCLF method includes convenient mixing rules (based on a QDO model) to easily calculate each of these dispersion coefficients between unlike atoms using only parameters of the individual atoms.^74^

Of key importance, the C_6_ dispersion coefficient does not equal the *r*^−6^ coefficient of the Lennard-Jones force field.^84^ Because the Lennard-Jones force field does not explicitly include higher-order dispersion (*e.g.*, C_8_, C_10_, *etc.*) terms, the Lennard-Jones *r*^−6^ coefficient must be made artificially higher than C_6_ to effectively compensate for the neglected C_8_ and C_10_ dispersion terms.^74,84^ Near the minimum energy separation between two atoms, higher-order dispersion (*e.g.*, C_8_, C_10_, *etc.*) can contribute ∼35% of the total dispersion energy.^79^

A QDO is a quantum harmonic oscillator containing a pseudoelectron attracted to a pseudonucleus.^85,86^ Three QDO parameters were computed for each atom in the material: (a) the pseudoelectron's effective charge, (b) the effective QDO frequency, and (c) the QDO's reduced mass.^74,85^ Force fields based on QDO models describe multibody dispersion and multibody polarization beyond the dipole approximation but require advanced simulation techniques.^85–87^

### 2.3 Quantifying electrostatic model accuracy

The root mean squared error (RMSE) of an electrostatic model quantifies its error compared to the quantum mechanically computed (*e.g.*, DFT) electrostatic potential over a chosen set of grid points:^88^2
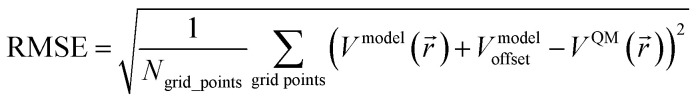
3
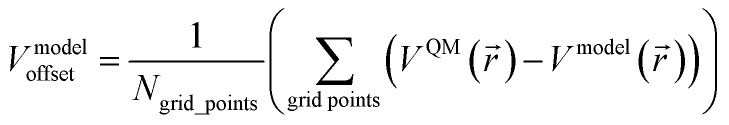
where *N*_grid_points_ is the number of grid points used to compute RMSE. *V*^model^_offset_ is the average difference between V^QM^(*r⃑*) and *V*^model^(*r⃑*) over these grid points.^89^ These grid points are normally chosen to be outside the material's van der Waals surface.^90^ For a MOF crystal, these grid points occur inside the MOF's pores.

In this study, the RMSE grid points were uniformly distributed in the volume of space that simultaneously met all three of the following criteria: (i) the material's total electron density, *ρ*(*r⃑*), was ≤10^−4^ e bohr^−3^, (ii) the grid point was no closer than 5 bohr to any atom in the material, and (iii) the grid point was closer than 12 bohr to at least one atom in the material. If any one of these three criteria were not met, the grid point was not used.

The relative root mean squared error (RRMSE) is the ratio of the electrostatic model's RMSE to the RMSE of a null model for which V^null_model^(*r⃑*) = 0.^91^ Note that *V*^null_model^_offset_ equals the average of V^QM^(*r⃑*) over the RMSE grid points, *V*^QM^_avg_. Therefore,4
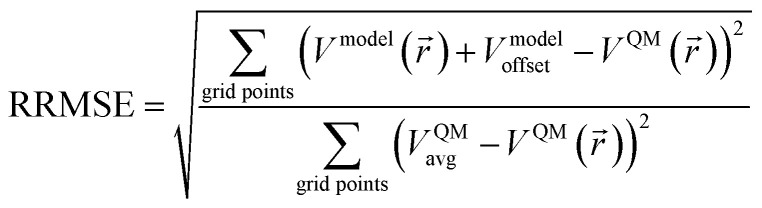
The RRMSE quantifies the fraction of electrostatic potential variations that are not described by the model. For example, an RRMSE = 0.1 means that 90% of the electrostatic potential variations are captured by the model and 10% are not. This RRMSE definition was used in several prior studies^76,88,91,92^ co-authored by one of us and differs from the even earlier literature^89,93^ by including the potential offsets in this RRMSE definition. These potential offsets must be included in the RMSE and RRMSE definitions, because in periodic materials the electrostatic potential has no obvious spatial position where it approaches zero value and the energy of a system having no net charge is invariant to a constant electrostatic potential shift.

In this work, the DFT electrostatic potential V^QM^(*r⃑*) was output from VASP using the keyword LVHAR = .true. We wrote an OpenMP parallelized Fortran program to compute RMSE and RRMSE. The RMSE and RRMSE were computed for the following four electrostatic models: (A) NACs only (aka monopole order), (B) NACs plus spherical cloud penetration, (C) NACs plus atomic dipoles (aka dipole order), and (D) NACs plus atomic dipoles plus spherical cloud penetration.

### 2.4 Structure acceptance or rejection

The 2014 CoRE MOF database was developed with goals of fixing disordered atoms, adding missing hydrogens, removing solvent molecules, discarding unfixable structures, and other cleaning protocols.^7^ These goals were partially but not fully achieved.

To partially address remaining structural deficiencies in the 2014 CoRE MOF database, we performed: (i) reasonability checks on our computed forcefield precursors for the calculated structures and (ii) connectivity checks for all structure categories. Table 2 lists reasons for rejecting a MOF structure.

**Table 2:** Reasons for rejecting MOF structures

Rejection reason	Identification method	Category
Unreasonable SBO for non-metal atom	DDEC6	Calculated
Negative NAC on metal atom	DDEC6	Calculated
RRMSE > 0.5	Direct calculation	Calculated
Isolated atom	Radii based connectivity	All
Unreasonable connectivity	Visual inspection	CSD and uncalculated

The sum of bond orders (SBO) was used as a screening criterion for reasonableness. DDEC6 bond orders and SBO for each atom in a material were computed using the Chargemol program.^69,94^Table 3 lists accepted SBO ranges for non-metal elements appearing in the database. A MOF structure was rejected if the SBO for any atom in the material was outside these ranges. Since a carbon atom has four valence electrons available to share in covalent bonding, its SBO is normally expected to be ∼4. The acceptable range of SBO for C atoms was set to [3.5, 4.75]. Since F and H have one electron to share in covalent bonding, their accepted SBO ranges were set to [0.5, 1.5]. The remaining halogens (Cl, Br, and I) had accepted SBO ranges of [0.5, 5.0] to accommodate situations in which these atoms were bonded to several O atoms (*e.g.*, ClO_4_^−^, BrO_4_^−^, IO_4_^−^) which can give relatively large SBOs. Oxygen atom has two electrons to share in covalent bonding but can also bond *via* lone-pairs (*e.g.*, Lewis acid–base interaction and hydrogen bonding); therefore, its accepted SBO range was set to [1.5, 3.0]. Boron and nitrogen atoms can exhibit variable bonding involving ∼3 or ∼4 covalent bonds (*e.g.*, BF_3_, B_2_H_6_, NH_3_, NH_4_^+^); therefore, accepted SBO ranges were set to [2.5, 4.5] for B and [2.5, 4.75] for N. The upper bound for N was slightly higher than for B to accommodate the formally higher SBO of N in nitrates than B in borates. Since Si prefers ∼4 bonds, its accepted SBO range was set to [3.5, 4.5]. The accepted SBO ranges for S, Se, and Te were set to [1.5, 6.0] to accommodate their ability to form ∼2 (*e.g.*, H_2_S, H_2_Se, H_2_Te) to ∼6 heuristic bonds (*e.g.*, SO_4_^2−^, SeO_4_^2−^, TeO_4_^2−^). The accepted SBO ranges for P and As were set to [3.0, 6.0] to accommodate their ability to form ∼3 (*e.g.*, PF_3_, AsF_3_) to ∼6 heuristic bonds (*e.g.*, H_2_PO_4_^−^, H_2_AsO_4_^−^). We do not claim these choices are perfect, but they can screen out some bad structures.

**Table 3:** Accepted ranges of SBOs for non-metal elements

H	[0.5, 1.5]	S	[1.5, 6.0]
B	[2.5, 4.5]	Cl	[0.5, 5.0]
C	[3.5, 4.75]	As	[3.0, 6.0]
N	[2.5, 4.75]	Se	[1.5, 6.0]
O	[1.5, 3.0]	Br	[0.5, 5.0]
F	[0.5, 1.5]	Te	[1.5, 6.0]
Si	[3.5, 4.5]	I	[0.5, 5.0]
P	[3.0, 6.0]		

A calculated structure was rejected if any metal atom had negative NAC. In this article, the definition of metal atom included all elements not listed in Table 3 except rare gases (He, Ne, Ar, Kr, Xe, Rn) and At. The rational for this criterion is that metal atoms in MOFs are typically bound to more electronegative elements (*e.g.*, N, O, *etc.*).

A calculated structure was rejected if any one of the four RRMSE described in Section 2.3 was greater than 0.5. This criterion ensured each of our four electrostatic models described the electrostatic potential with an error less than half of a no charges model. In other words, each electrostatic model described the majority of electrostatic potential variations in the MOF's pores.

Structures were rejected from all categories if they contained any isolated atoms. An atom was considered isolated if it was not connected to any other atom based on the radii listed in Table 5.

For CSD and uncalculated structures, some structures were rejected based on strange connectivity identified by visual inspection. Because this visual inspection was not performed systematically, we do not claim all instances of bad connectivity were identified.

Table 4 lists the category breakdown for 2014 CoRE MOF structures. Calculated structures were those for which an electron density distribution was calculated using DFT. Uncalculated structures were non-CSD structures for which the electron density distribution was not calculated. This could be due to one of two factors: (i) the MOF structure was so large that DFT calculation was not practical within our computational budget or (ii) the DFT calculation did not readily converge. CSD structures were those for which the geometry came directly from the CSD database.^7^ We do not report forcefield precursors for the CSD structures, because CSD licensing terms may not allow us to publicly distribute their geometries.

**Table 4:** Category breakdown for 2014 CoRE MOF structures

	Total structures	Rejected	Accepted
Calculated	4445	1389	3056
Uncalculated	319	63	256
CSD	345	49	296
Total	5109	1501	3608

The second column of Table 4 lists the total number of structures in each category. The third and fourth columns list the number of rejected and non-rejected structures in each category, respectively. The ESI† contains detailed lists of MOFs in each category. For each rejected structure, the ESI† lists the specific reason(s) that structure was rejected.

## 3. Results and discussion

### 3.1 Forcefield precursors

Files containing forcefield precursors for 3056 accepted calculated structures are contained in the ESI.† The data collected for each atom in a MOF includes: the coordinates; the net atomic charge (NAC); C_6_, C_8_ and C_10_ dispersion coefficients; fluctuating polarizability; forcefield polarizability; electron cloud parameters; 〈*r*^3^〉 and 〈 *r*^4^〉 moments; QDO parameters; atomic dipole and quadrupole; atomic screened static polarizability tensor and isotropic polarizability; atomic spin moment. The data collected for the MOF's unit cell includes: the lattice vectors, the total spin magnetic moment, the net charge, and the RRMSE of the electrostatic potential for electrostatic models including (A) NACs only, (B) NACs plus spherical cloud penetration, (C) NACs plus atomic dipoles, and (D) NACs plus atomic dipoles and spherical cloud penetration. For each MOF, these data were written to a *xyz* file whose chemical structure can be visualized with the Jmol^95,96^ program. In the future, these forcefield precursors could be used as building blocks to construct force fields for these materials.

### 3.2 RRMSE of electrostatic potential

**Fig. 4:**
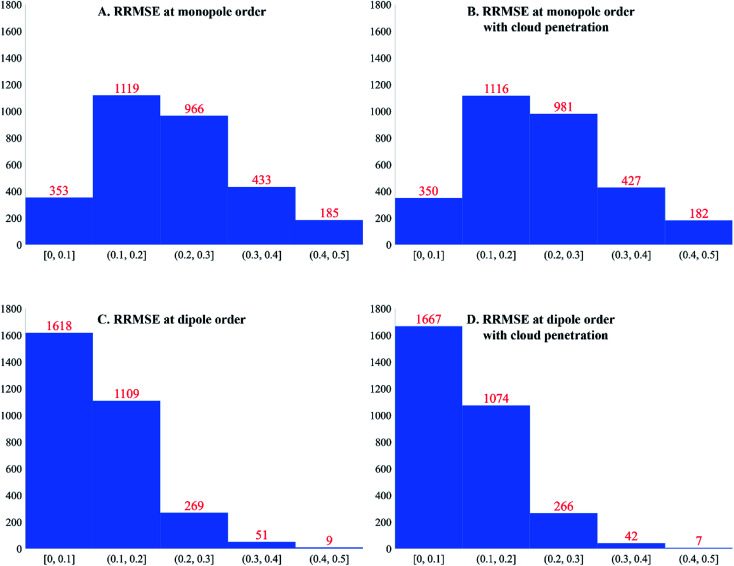
Histograms of electrostatic potential RRMSE (dimensionless) for accepted calculated structures.

Fig. 4 plots histograms of electrostatic potential RRMSE for the accepted calculated structures. At monopole order (with or without spherical cloud penetration), the histograms peak at RRMSE = (0.1, 0.2). At both monopole and dipole orders, spherical cloud penetration had negligible effect on the histograms. Dipole order showed dramatic improvement over monopole order.

Some additional comments are in order. Even though spherical cloud penetration had negligible effect on the RRMSE values, this does not necessarily mean charge penetration effects are not important for constructing accurate electrostatic models. In MOF's, the RRMSE probes volume in space that is likely occupied by an adsorbate atom's nuclear position. Because charge penetration extends over the volume occupied by an adsorbate atom's full electron density distribution, it affects the electrostatic potential over a much larger volume than that probed by RRMSE calculation. For this reason, charge penetration can be more important than indicated by the RRMSE values. Prior studies demonstrated intermolecular electrostatic interaction energies are substantially improved by including charge penetration.^97–99^ Therefore, including spherical cloud penetration (aka ‘charge penetration’) will likely improve the adsorbate–MOF and adsorbate–adsorbate electrostatic interaction energies even though its effects on RRMSE were negligible.

When constructing force fields, decisions have to be made about tradeoffs between simplicity and accuracy. For best accuracy, the force field should include NACs, atomic dipoles (and possibly atomic quadrupoles), and spherical charge penetration. However, the simplest force field would include NACs only and neglect atomic multipoles and charge penetration.

### 3.3 Atom types

Atom types are widely used in force fields.^29^ The main idea of atom typing is to classify similar atoms into the same atom type to facilitate forcefield parameterization, where all atoms of the same atom type are normally assigned identical forcefield parameters.^32^ Biomolecular force fields have used atom types for several decades.^100–106^ The universal force field (UFF) and its extensions to MOFs (*e.g.*, UFF4MOF) focused on atom types for structural optimizations.^107–109^

The connectivity based atom contribution (CBAC) method defined atom types in porous materials (*e.g.*, MOFs and covalent organic frameworks) using first neighbors only.^110,111^ Two significant limitations of the CBAC method are: (i) its atom types are based on first neighbors only which limits chemical transferability and (ii) the NACs for its atom types were obtained by CHELPG analysis of small fragment clusters but it is unclear how accurately these may reproduce the fully periodic electrostatic potential.^110,111^

**Fig. 5:**
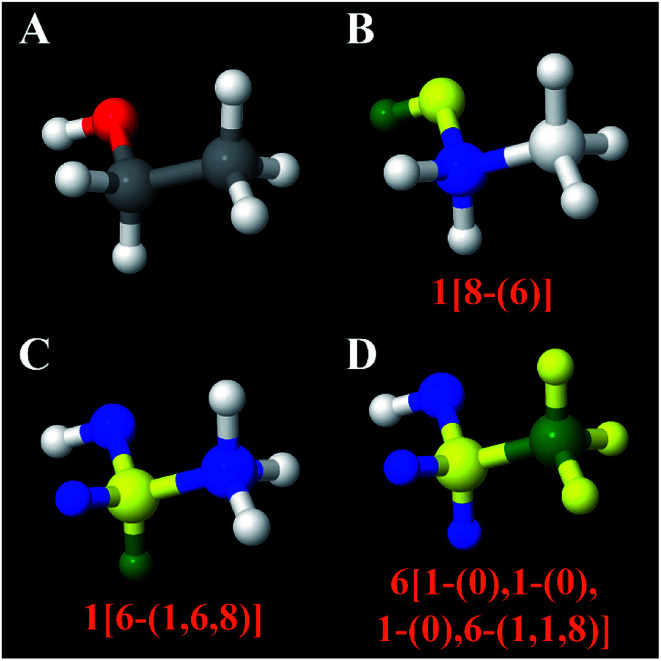
Illustration of labeling atom types. Panel (A) shows an ethanol molecule with atoms colored by element: grey (C), red (O), white (H). Panels (B–D) illustrate the typing of different atoms in this molecule. In each panel, the chosen atom is colored green. Its first neighbors are colored yellow. Its second neighbors are colored blue. In Panel (B), the hydrogen in the hydroxy group is chosen as the central atom. It is connected to one oxygen that is connected to a carbon atom plus the central atom. In Panel (C), the α-H is chosen as the central atom. It is connected to one carbon that is connected to one hydrogen, one carbon, and one oxygen plus the central atom. In Panel (D), the β-C is chosen as the central atom. It is connected to three hydrogens and one carbon. The carbon is connected to two hydrogens and one oxygen plus the central atom.

In our work, atom types are defined based on both first and second neighbors leading to better chemical transferability. Also, the NACs for our atom types were calculated using the fully periodic electrostatic potential, rather than small fragment clusters. Fig. 5 illustrates our atom typing scheme. When multiple neighbor groups are present, they are sorted by the atomic number of the neighbor atoms from smallest to largest. If a first neighbor has no second neighbors, its lack of second neighbor is indicated by (0) in the atom type label. For example, the atom type 6[1-(0),1-(0),1-(0),6-(1,1,8)] indicates a central carbon atom with four first neighbors (H, H, H, C) where each of the first neighbor H atoms is not directly bonded to any second neighbors and the first-neighbor C atom is directly bonded to H, H, and O plus the central atom. As another example, the atom type 15[6-(6),6-(6,6),6-(6,6),29-(7,7,7)] demonstrates that 6-(6) is listed prior to 6-(6,6). As another example, the atom type 7[1-(0),6-(1,1,6),6-(6,8)] demonstrates that 6-(1,1,6) is listed prior to 6-(6,8).

During atom typing, two atoms were considered directly connected if the distance between them was no greater than the sum of the atom typing radii listed in Table 5. These radii have the following history. Starting with the atom connectivity radii of OpenBabel version 1.100.1, we added 0.18 Å (*i.e.*, a skin distance was incorporated into the radii). Second, we reduced some transition metal radii to eliminate unreasonable metal–metal bonds and excessively high coordination numbers. Third, the carbon and oxygen radii were slightly increased to maintain carbon/oxygen–metal bonds after decreasing the metal radii. These radii values are unique only in an approximate sense, because small increases or decreases in the radii values may be feasible. Large changes in these radii values are not feasible, because bad atom connectivity would result.

**Table 5:** Radii (Å) used for atom typing. Two atoms were considered directly connected if the distance between them was no greater than the sum of these radii

H	0.38	Cr	1.53	Pd	1.68	Er	1.80
Li	0.86	Mn	1.53	Ag	1.56	Tm	1.84
Be	0.53	Fe	1.43	Cd	1.56	Yb	1.80
B	1.01	Co	1.31	In	1.53	Lu	1.86
C	0.88	Ni	1.33	Sn	1.64	Hf	1.73
N	0.86	Cu	1.31	Sb	1.64	W	1.33
O	0.89	Zn	1.41	Te	1.65	Re	1.29
F	0.82	Ga	1.40	I	1.58	Ir	1.50
Na	1.15	Ge	1.35	Cs	1.85	Pt	1.66
Mg	1.28	As	1.39	Ba	1.52	Au	1.68
Al	1.53	Se	1.40	La	1.91	Hg	1.88
Si	1.38	Br	1.39	Ce	1.98	Pb	1.72
P	1.28	Rb	1.65	Pr	1.75	Bi	1.72
S	1.20	Sr	1.30	Nd	1.92	Th	1.97
Cl	1.17	Y	1.84	Sm	1.89	U	1.76
K	1.44	Zr	1.73	Eu	1.83	Np	1.73
Ca	1.17	Nb	1.66	Gd	1.79	Pu	1.71
Sc	1.62	Mo	1.57	Tb	1.82		
Ti	1.65	Ru	1.58	Dy	1.79		
V	1.51	Rh	1.63	Ho	1.63		

These atom type definitions gave a total of 1313 first-neighbor atom types and 7033 second-neighbor atom types for the 3056 accepted calculated MOFs. Of these, 783 first-neighbor and 3015 second-neighbor atom types appeared in more than one MOF.

**Fig. 6:**
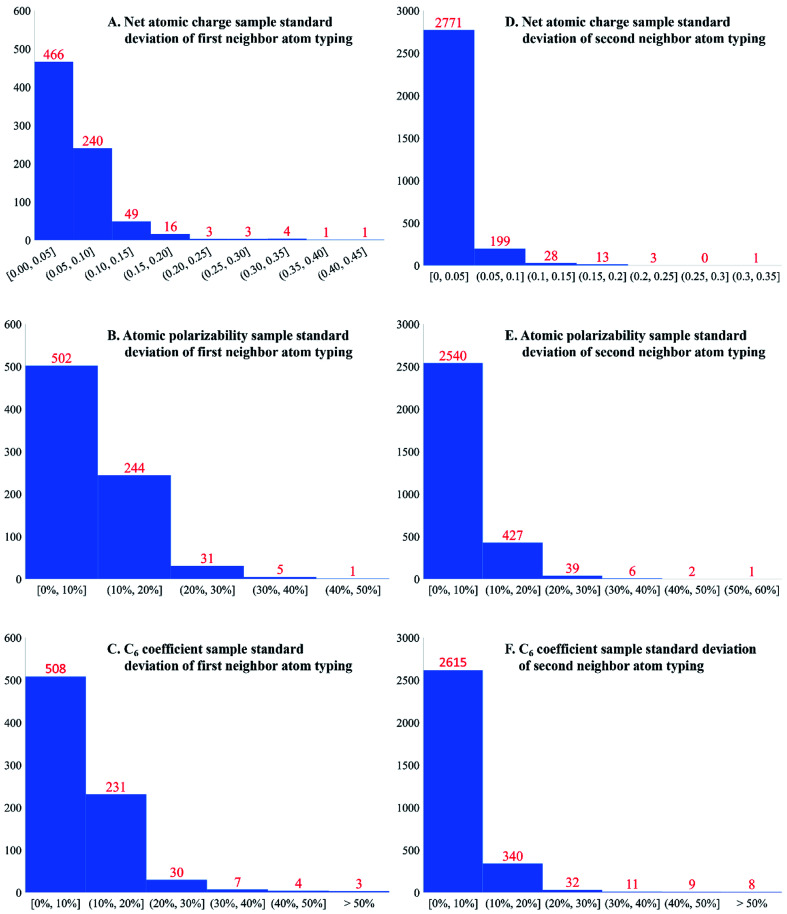
Histograms of standard deviations of NACs, forcefield polarizabilities, and C_6_ dispersion coefficients for first-neighbor-based and second-neighbor-based atom types. Second-neighbor-based atom types included both first and second neighbors. Each standard deviation was computed across different occurrences of the same atom type. The second-neighbor-based atom types showed much smaller standard deviations than the first-neighbor-based atom types.

Is second-neighbor-based atom typing optimal? To explore this question, the averages and standard deviations of 17 different forcefield precursors were computed and listed in the ESI† for each first-neighbor-based and each second-neighbor-based atom type: NAC; C_6_, C_8_, and C_10_ dispersion coefficients; fluctuating, forcefield, and static polarizabilities; 〈*r*^3^〉 and 〈*r*^4^〉 radial moments; *a*, *b*, and *R*^2^ electron cloud parameters; atomic dipole moment magnitude; three QDO parameters; and atomic spin moment. Fig. 6 presents histograms of the standard deviations for NAC, forcefield polarizability, and C_6_ across first-neighbor-based and second-neighbor-based atom types. Only atom types appearing in more than one accepted calculated structure were included in this analysis. Second-neighbor-based atom types showed remarkably better chemical transferability compared to first-neighbor-based atom types. For example, 91.9% of the second-neighbor-based atom types had NAC standard deviations ≤0.05*e*, while only 59.5% of the first-neighbor-based atom types did.

This atom typing is theoretically justified, because in organic molecules electronic effects play a major role in group reactivity and induction contributes a significant portion of that effect. In organic chemistry, the inductive effect is the electron donating or withdrawing ability of a chemical group that can be transmitted to other parts of the molecule through chemical bonds.^112^ The inductive effect usually decreases across each bond and is usually limited to 2 to 3 bonds lengths.^112^ Our decision to base atom typing on first and second neighbors is a pragmatic one. As shown in Fig. 6, using only first neighbors leads to large standard deviations in the calculated forcefield parameters. The standard deviation is greatly reduced by incorporating second neighbors in the atom types. Incorporating third neighbors into the atom type definition would lead to an enormous number of different atom types, thus making forcefield parameterization too tedious. Therefore, second-neighbor-based atom types are optimal.

**Fig. 7:**
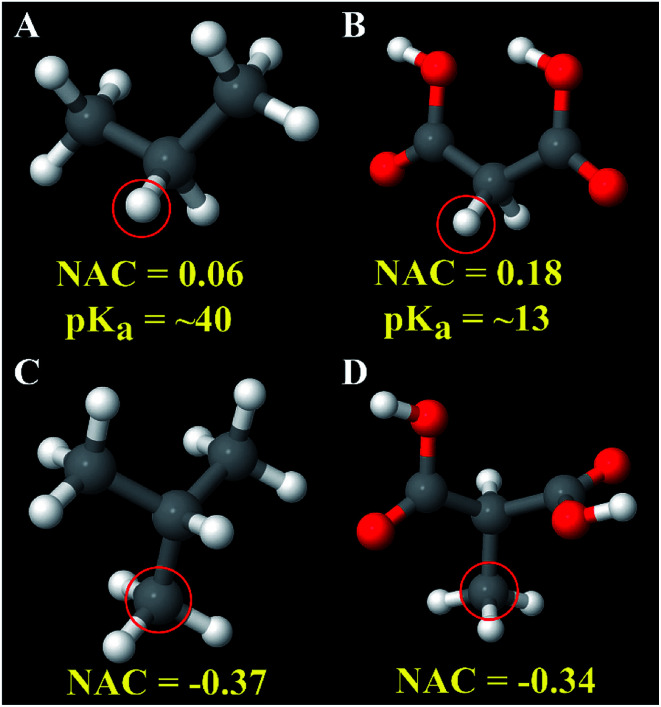
Atoms of the same atom type can sometimes have different chemical reactivities. Panels (A and B) show hydrogen atoms having the same atom type but very different p*K*_a_ values. The approximate p*K*_a_ values are from ref. 113. Panels (C and D) show the good transferability of carbon atom NAC for analogous compounds in which the circled hydrogen atoms in Panels (A and B) were replaced by a methyl group.

Fig. 7 explores an extreme case of third neighbor inductive effects. In malonic acid (Fig. 7B), the four highly electronegative oxygen groups withdraw electrons increasing the acidity of the circled hydrogen atom compared to the circled hydrogen in propane (Fig. 7A). Specifically, the circled hydrogen in Fig. 7B can be stripped away from the carbon atom by strong base while the one in Fig. 7A does not possess the same reactivity. The NACs of those hydrogens are 0.06 for Fig. 7A and 0.18 for Fig. 7B. If these hydrogens are replaced with a methyl group, the methyl carbon NACs are −0.37 and −0.34 respectively. These differences in NACs are within an acceptable range for atoms sharing the same atom type.

Table 6 summarizes the breakdown of atom types in the 3608 accepted structures. A total of 8607 different atom types appeared in these structures. 3015 atom types appeared in more than one accepted calculated structure, while 4018 atom types appeared in only one accepted calculated structure. An additional 1574 atom types appeared only in the uncalculated and/or CSD structures. These results clearly show that many atom types appeared in only one MOF in the dataset. These results also show the nearly infinite chemical diversity of MOFs. For example, the 296 accepted CSD structures contained 320 new atom types that did not appear in either the calculated or uncalculated structures.

**Table 6:** Breakdown of atom types in accepted structures

Appeared in more than one calculated MOFs	3015
Appeared in only one calculated MOF	4018
Unique to uncalculated MOFs	1241
Unique to CSD MOFs	320
Appeared in both uncalculated and CSD MOFs but not calculated MOFs	13
Total	8607

Do a relatively small number of atom types completely describe a large percentage of the accepted calculated structures? Table 7 summarizes calculations performed to explore this question. Starting with a ‘frequency threshold’, any MOF containing any atom type that appeared in fewer than ‘frequency threshold’ different MOFs (within the 3056 accepted calculated structures) was discarded. All atom types appearing in the remaining MOFs occurred in at least ‘frequency threshold’ different MOFs in the 3056 accepted calculated structures. Then, the number of distinct atom types appearing in the remaining MOFs was counted and reported in the second column of Table 7. The results show the chemical diversity of MOFs in this dataset is well-mixed. In other words, there was not a large segregated subset of MOFs in this dataset that are completely described by a few atom types.

**Table 7:** Exploration of most important atom types in accepted calculated structures. Each atom type appearing in the enumerated MOFs appeared in at least ‘frequency threshold’ different accepted calculated structures. The listed number of atom types is the number of different atom types in these enumerated MOFs

Frequency threshold	# atom types	# MOFs	Frequency threshold	# atom types	# MOFs
5	1167	1120	30	166	219
10	572	682	35	138	188
15	385	508	40	120	151
20	273	334	45	106	107
25	208	268	50	94	77

Zn and Cu were the most common metal elements in the 2014 CoRE MOF database.^7^ Among the 3056 accepted calculated structures, 785 structures were built entirely of the elements Cu, Zn, C, H, N, and O. These 785 structures contained 1314 different atom types.

**Fig. 8:**
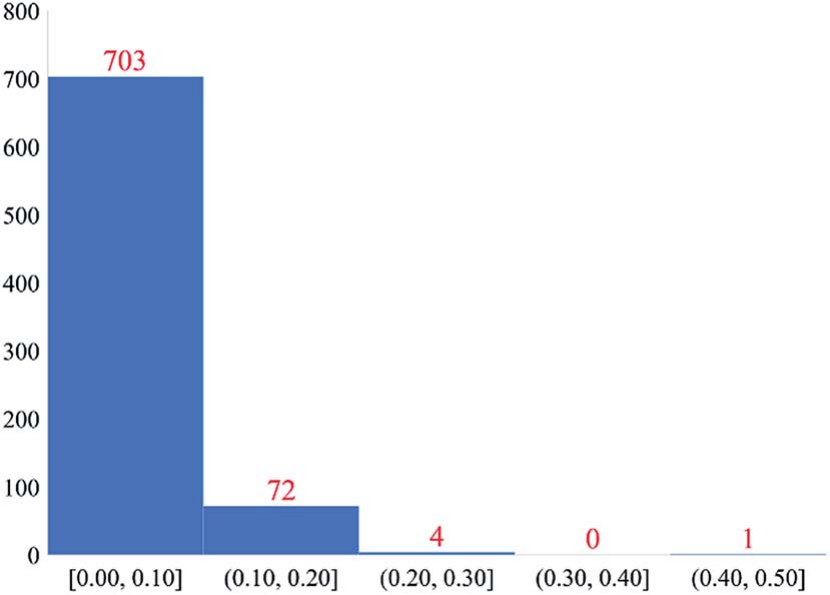
Histogram of absolute difference of average DDEC6 NAC between 130k molecules dataset and 3056 accepted calculated MOFs dataset. This histogram includes 780 atom types that have more than one occurrence in each dataset.

Finally, we tested the transferability of atom typing across different classes of materials. Applying the second-neighbor atom typing procedure discussed above to the molecular test set of Bleiziffer *et al.*,^62^ we obtained 32 530 atom types from 130 265 molecules in the dataset (aka ‘130k’ dataset). (Bleiziffer *et al.*'s dataset contained 130 267 structures, but we did not include the two of these structures containing unbonded atoms.) The DDEC6 NACs for these molecules are obtained from Bleiziffer *et al.*'s data for a dielectric constant of 4.^62^ Of the atom types from the 130k molecules and 3056 accepted calculated CoRE MOFs, 798 are shared in both datasets and 780 of these have more than one occurrence (in the same or multiple structures) in each dataset. Fig. 8 is a histogram of the absolute difference of the average DDEC6 NAC between the 130k molecules dataset and the 3056 accepted calculated MOF dataset for these 780 atom types. As shown in Fig. 8, most of these atom types had an average DDEC6 NAC absolute difference less than 0.1. This shows our atom typing scheme has good chemical transferability and can be applied to different material types. Also, Bleiziffer *et al.* trained a machine learning model on the 130k dataset to predict DDEC6 NACs with high accuracy.^62^ The similar DDEC6 NAC between CoRE MOF and 130k molecules datasets for a shared atom type and Bleiziffer *et al.*'s successful machine learning model indicate it should be feasible to train a machine learning model to predict the properties of new atom types. Due to the extremely large number of distinct atom types we found in these datasets, a machine learning model could be highly useful to assign force-field parameter values for such a large number of atom types.

## 4. Conclusions

In this article, we considered the problem of how to automatically extract forcefield precursors from quantum chemistry calculations using DDEC6 atomic population analysis. We focused on calculating non-bonded parameters for each atom in the material: net atomic charge, atomic dipole and quadrupole, electron cloud parameters, atomic spin moment, dispersion coefficients, various polarizabilities, and QDO parameters. Our first main result was calculated values of these atomistic descriptors for 3056 MOFs that will serve as building blocks to construct classical force fields for these materials.

Regarding the electrostatic parameters, our results confirm the general belief that NACs can often reproduce the electrostatic potential surrounding a material with acceptable accuracy. Including atomic dipoles dramatically reduced the electrostatic potential errors; therefore, it is preferable to include atomic dipoles in the electrostatic model. Spherical cloud penetration had negligible effect on the RRMSE histograms; however, charge penetration effects can still be important at short interatomic distances.

Our second main result was the practical assessment of atom typing for MOFs. We improved values of the atom typing radii that define whether two atoms in a material are directly bonded to each other. Our results showed that atom types based on both first and second neighbors adequately captures the chemical environment, but atom types based on only first neighbors does not. Specifically, the standard deviation of calculated forcefield precursors was relatively large across atoms sharing similar first neighbor environments but relatively small across atoms sharing similar first and second neighbor environments. Including third neighbors in the atom type definition would create an unnecessarily large and burdensome number of different atom types. Therefore, atom typing including both first and second neighbors is optimal.

Our results demonstrate the large chemical diversity of MOFs. 8607 different atom types were identified in the 3608 non-rejected MOFs. These atom types should be useful to develop future force fields for MOFs. Although the MOF to atom type ratio was lower than 1.0 in our study, there are two key reasons to be believe this ratio will improve in the future: (1) the number of chemical elements from which MOFs can be synthesized is practically limited to ∼100, because heavier chemical elements undergo rapid radioactive decay. Therefore, new MOFs will reuse many of the same chemical elements. (2) There is an almost infinite number of different ways to combine common metals and common organic linkers to form MOFs.^18,19^ To date, only a tiny fraction of these hypothetical MOFs have been chemically synthesized.^18,19^ As more and more of these hypothetical MOFs are synthesized, many atom types will be reused leading to a higher MOF to atom type ratio. Moreover, parameterizing only the popular atom types could describe a substantial percentage of MOFs with higher MOF to atom type ratio.

In summary, more research is urgently needed to develop accurate interaction models for MOFs. Because of the large chemical diversity of real and hypothetical MOFs, it is impractical to evaluate all possible MOFs experimentally. Therefore, computational assessment is required. The interaction model is a crucial ingredient of computational assessment. Two major types of interaction models are exchange–correlation theories (*e.g.*, DFT + dispersion) and classical force fields. The former needs orders of magnitude improvement in computational speed to yield rapid *ab initio* molecular dynamics. The latter requires extensive parameterization to yield accurate force fields. To actually develop working force fields, the non-bonded interaction terms studied herein will need to be combined with bonded interaction terms (*e.g.*, bond springs, angle springs, torsion and/or out-of-plane parameters) and further parameterization of short-range exchange-repulsion. However, in simulations using a rigid framework approximation (*e.g.*, Monte Carlo simulations of adsorption in rigid frameworks), the bonded interaction terms are not required.

## Funding

National Science Foundation (NSF) CAREER Award DMR-1555376 provided financial support. Supercomputing resources were provided by the Extreme Science and Engineering Discovery Environment (XSEDE).^114^ XSEDE is funded by NSF grant ACI-1548562. XSEDE project grant TG-CTS100027 provided allocations on the Stampede2 cluster at the Texas Advanced Computing Center and the Comet cluster at the San Diego Supercomputing Center.

## Authors' contributions

T. C. performed the calculations and wrote Python scripts to prepare input files, analyze output files, perform atom typing, determine whether to accept or reject structures, and write files containing force-field precursors and atom type statistics. T. A. M. wrote the programs to compute the electrostatic potential RRMSE and electron cloud parameters. T. A. M. obtained funding for the project. Both authors designed the study, interpreted data, and wrote the manuscript.

## Conflicts of interest

There are no conflicts of interest to declare.

## Supplementary Material

RA-009-C9RA07327B-s001

RA-009-C9RA07327B-s002

RA-009-C9RA07327B-s003

RA-009-C9RA07327B-s004

RA-009-C9RA07327B-s005

RA-009-C9RA07327B-s006

RA-009-C9RA07327B-s007

RA-009-C9RA07327B-s008

RA-009-C9RA07327B-s009

RA-009-C9RA07327B-s010

RA-009-C9RA07327B-s011

RA-009-C9RA07327B-s012

RA-009-C9RA07327B-s013

RA-009-C9RA07327B-s014

RA-009-C9RA07327B-s015

RA-009-C9RA07327B-s016

RA-009-C9RA07327B-s017

RA-009-C9RA07327B-s018

RA-009-C9RA07327B-s019

RA-009-C9RA07327B-s020

RA-009-C9RA07327B-s021

RA-009-C9RA07327B-s022

RA-009-C9RA07327B-s023

RA-009-C9RA07327B-s024

RA-009-C9RA07327B-s025

RA-009-C9RA07327B-s026

RA-009-C9RA07327B-s027

RA-009-C9RA07327B-s028

RA-009-C9RA07327B-s029

RA-009-C9RA07327B-s030

RA-009-C9RA07327B-s031

RA-009-C9RA07327B-s032

RA-009-C9RA07327B-s033

RA-009-C9RA07327B-s034

RA-009-C9RA07327B-s035

RA-009-C9RA07327B-s036

RA-009-C9RA07327B-s037

RA-009-C9RA07327B-s038

RA-009-C9RA07327B-s039

RA-009-C9RA07327B-s040

RA-009-C9RA07327B-s041

RA-009-C9RA07327B-s042

RA-009-C9RA07327B-s043

RA-009-C9RA07327B-s044

RA-009-C9RA07327B-s045

RA-009-C9RA07327B-s046

RA-009-C9RA07327B-s047

RA-009-C9RA07327B-s048

RA-009-C9RA07327B-s049

RA-009-C9RA07327B-s050

RA-009-C9RA07327B-s051

RA-009-C9RA07327B-s052

RA-009-C9RA07327B-s053

RA-009-C9RA07327B-s054

RA-009-C9RA07327B-s055

RA-009-C9RA07327B-s056

RA-009-C9RA07327B-s057

RA-009-C9RA07327B-s058

RA-009-C9RA07327B-s059

RA-009-C9RA07327B-s060

RA-009-C9RA07327B-s061

RA-009-C9RA07327B-s062

RA-009-C9RA07327B-s063

RA-009-C9RA07327B-s064

RA-009-C9RA07327B-s065

RA-009-C9RA07327B-s066

RA-009-C9RA07327B-s067

RA-009-C9RA07327B-s068

RA-009-C9RA07327B-s069

RA-009-C9RA07327B-s070

RA-009-C9RA07327B-s071

RA-009-C9RA07327B-s072

RA-009-C9RA07327B-s073

RA-009-C9RA07327B-s074

RA-009-C9RA07327B-s075

RA-009-C9RA07327B-s076

RA-009-C9RA07327B-s077

RA-009-C9RA07327B-s078

RA-009-C9RA07327B-s079

RA-009-C9RA07327B-s080

RA-009-C9RA07327B-s081

RA-009-C9RA07327B-s082

RA-009-C9RA07327B-s083

RA-009-C9RA07327B-s084

RA-009-C9RA07327B-s085

RA-009-C9RA07327B-s086

RA-009-C9RA07327B-s087

RA-009-C9RA07327B-s088

RA-009-C9RA07327B-s089

RA-009-C9RA07327B-s090

RA-009-C9RA07327B-s091

RA-009-C9RA07327B-s092

RA-009-C9RA07327B-s093

RA-009-C9RA07327B-s094

RA-009-C9RA07327B-s095

RA-009-C9RA07327B-s096

RA-009-C9RA07327B-s097

RA-009-C9RA07327B-s098

RA-009-C9RA07327B-s099

RA-009-C9RA07327B-s100

RA-009-C9RA07327B-s101

RA-009-C9RA07327B-s102

RA-009-C9RA07327B-s103

RA-009-C9RA07327B-s104

RA-009-C9RA07327B-s105

RA-009-C9RA07327B-s106

RA-009-C9RA07327B-s107

RA-009-C9RA07327B-s108

RA-009-C9RA07327B-s109

RA-009-C9RA07327B-s110

RA-009-C9RA07327B-s111

RA-009-C9RA07327B-s112

RA-009-C9RA07327B-s113

RA-009-C9RA07327B-s114

RA-009-C9RA07327B-s115

RA-009-C9RA07327B-s116

RA-009-C9RA07327B-s117

RA-009-C9RA07327B-s118

RA-009-C9RA07327B-s119

RA-009-C9RA07327B-s120

RA-009-C9RA07327B-s121

RA-009-C9RA07327B-s122

RA-009-C9RA07327B-s123

RA-009-C9RA07327B-s124

RA-009-C9RA07327B-s125

RA-009-C9RA07327B-s126

RA-009-C9RA07327B-s127

RA-009-C9RA07327B-s128

RA-009-C9RA07327B-s129

RA-009-C9RA07327B-s130

RA-009-C9RA07327B-s131

RA-009-C9RA07327B-s132

RA-009-C9RA07327B-s133

RA-009-C9RA07327B-s134

RA-009-C9RA07327B-s135

RA-009-C9RA07327B-s136

RA-009-C9RA07327B-s137

RA-009-C9RA07327B-s138

RA-009-C9RA07327B-s139

RA-009-C9RA07327B-s140

RA-009-C9RA07327B-s141

RA-009-C9RA07327B-s142

RA-009-C9RA07327B-s143

RA-009-C9RA07327B-s144

RA-009-C9RA07327B-s145

RA-009-C9RA07327B-s146

RA-009-C9RA07327B-s147

RA-009-C9RA07327B-s148

RA-009-C9RA07327B-s149

RA-009-C9RA07327B-s150

RA-009-C9RA07327B-s151

RA-009-C9RA07327B-s152

RA-009-C9RA07327B-s153

RA-009-C9RA07327B-s154

RA-009-C9RA07327B-s155

RA-009-C9RA07327B-s156

RA-009-C9RA07327B-s157

RA-009-C9RA07327B-s158

RA-009-C9RA07327B-s159

RA-009-C9RA07327B-s160

RA-009-C9RA07327B-s161

RA-009-C9RA07327B-s162

RA-009-C9RA07327B-s163

RA-009-C9RA07327B-s164

RA-009-C9RA07327B-s165

RA-009-C9RA07327B-s166

RA-009-C9RA07327B-s167

RA-009-C9RA07327B-s168

RA-009-C9RA07327B-s169

RA-009-C9RA07327B-s170

RA-009-C9RA07327B-s171

RA-009-C9RA07327B-s172

RA-009-C9RA07327B-s173

RA-009-C9RA07327B-s174

RA-009-C9RA07327B-s175

RA-009-C9RA07327B-s176

RA-009-C9RA07327B-s177

RA-009-C9RA07327B-s178

RA-009-C9RA07327B-s179

RA-009-C9RA07327B-s180

RA-009-C9RA07327B-s181

RA-009-C9RA07327B-s182

RA-009-C9RA07327B-s183

RA-009-C9RA07327B-s184

RA-009-C9RA07327B-s185

RA-009-C9RA07327B-s186

RA-009-C9RA07327B-s187

RA-009-C9RA07327B-s188

RA-009-C9RA07327B-s189

RA-009-C9RA07327B-s190

RA-009-C9RA07327B-s191

RA-009-C9RA07327B-s192

RA-009-C9RA07327B-s193

RA-009-C9RA07327B-s194

RA-009-C9RA07327B-s195

RA-009-C9RA07327B-s196

RA-009-C9RA07327B-s197

RA-009-C9RA07327B-s198

RA-009-C9RA07327B-s199

RA-009-C9RA07327B-s200

RA-009-C9RA07327B-s201

RA-009-C9RA07327B-s202

RA-009-C9RA07327B-s203

RA-009-C9RA07327B-s204

RA-009-C9RA07327B-s205

RA-009-C9RA07327B-s206

RA-009-C9RA07327B-s207

RA-009-C9RA07327B-s208

RA-009-C9RA07327B-s209

RA-009-C9RA07327B-s210

RA-009-C9RA07327B-s211

RA-009-C9RA07327B-s212

RA-009-C9RA07327B-s213

RA-009-C9RA07327B-s214

RA-009-C9RA07327B-s215

RA-009-C9RA07327B-s216

RA-009-C9RA07327B-s217

RA-009-C9RA07327B-s218

RA-009-C9RA07327B-s219

RA-009-C9RA07327B-s220

RA-009-C9RA07327B-s221

RA-009-C9RA07327B-s222

RA-009-C9RA07327B-s223

RA-009-C9RA07327B-s224

RA-009-C9RA07327B-s225

RA-009-C9RA07327B-s226

RA-009-C9RA07327B-s227

RA-009-C9RA07327B-s228

RA-009-C9RA07327B-s229

RA-009-C9RA07327B-s230

RA-009-C9RA07327B-s231

RA-009-C9RA07327B-s232

RA-009-C9RA07327B-s233

RA-009-C9RA07327B-s234

RA-009-C9RA07327B-s235

RA-009-C9RA07327B-s236

RA-009-C9RA07327B-s237

RA-009-C9RA07327B-s238

RA-009-C9RA07327B-s239

RA-009-C9RA07327B-s240

RA-009-C9RA07327B-s241

RA-009-C9RA07327B-s242

RA-009-C9RA07327B-s243

RA-009-C9RA07327B-s244

RA-009-C9RA07327B-s245

RA-009-C9RA07327B-s246

RA-009-C9RA07327B-s247

RA-009-C9RA07327B-s248

RA-009-C9RA07327B-s249

RA-009-C9RA07327B-s250

RA-009-C9RA07327B-s251

RA-009-C9RA07327B-s252

RA-009-C9RA07327B-s253

RA-009-C9RA07327B-s254

RA-009-C9RA07327B-s255

RA-009-C9RA07327B-s256

RA-009-C9RA07327B-s257

RA-009-C9RA07327B-s258

RA-009-C9RA07327B-s259

RA-009-C9RA07327B-s260

RA-009-C9RA07327B-s261

RA-009-C9RA07327B-s262

RA-009-C9RA07327B-s263

RA-009-C9RA07327B-s264

RA-009-C9RA07327B-s265

RA-009-C9RA07327B-s266

RA-009-C9RA07327B-s267

RA-009-C9RA07327B-s268

RA-009-C9RA07327B-s269

RA-009-C9RA07327B-s270

RA-009-C9RA07327B-s271

RA-009-C9RA07327B-s272

RA-009-C9RA07327B-s273

RA-009-C9RA07327B-s274

RA-009-C9RA07327B-s275

RA-009-C9RA07327B-s276

RA-009-C9RA07327B-s277

RA-009-C9RA07327B-s278

RA-009-C9RA07327B-s279

RA-009-C9RA07327B-s280

RA-009-C9RA07327B-s281

RA-009-C9RA07327B-s282

RA-009-C9RA07327B-s283

RA-009-C9RA07327B-s284

RA-009-C9RA07327B-s285

RA-009-C9RA07327B-s286

RA-009-C9RA07327B-s287

RA-009-C9RA07327B-s288

RA-009-C9RA07327B-s289

RA-009-C9RA07327B-s290

RA-009-C9RA07327B-s291

RA-009-C9RA07327B-s292

RA-009-C9RA07327B-s293

RA-009-C9RA07327B-s294

RA-009-C9RA07327B-s295

RA-009-C9RA07327B-s296

RA-009-C9RA07327B-s297

RA-009-C9RA07327B-s298

RA-009-C9RA07327B-s299

RA-009-C9RA07327B-s300

RA-009-C9RA07327B-s301

RA-009-C9RA07327B-s302

RA-009-C9RA07327B-s303

RA-009-C9RA07327B-s304

RA-009-C9RA07327B-s305

RA-009-C9RA07327B-s306

RA-009-C9RA07327B-s307

RA-009-C9RA07327B-s308

RA-009-C9RA07327B-s309

RA-009-C9RA07327B-s310

RA-009-C9RA07327B-s311

RA-009-C9RA07327B-s312

RA-009-C9RA07327B-s313

RA-009-C9RA07327B-s314

RA-009-C9RA07327B-s315

RA-009-C9RA07327B-s316

RA-009-C9RA07327B-s317

RA-009-C9RA07327B-s318

RA-009-C9RA07327B-s319

RA-009-C9RA07327B-s320

RA-009-C9RA07327B-s321

RA-009-C9RA07327B-s322

RA-009-C9RA07327B-s323

RA-009-C9RA07327B-s324

RA-009-C9RA07327B-s325

RA-009-C9RA07327B-s326

RA-009-C9RA07327B-s327

RA-009-C9RA07327B-s328

RA-009-C9RA07327B-s329

RA-009-C9RA07327B-s330

RA-009-C9RA07327B-s331

RA-009-C9RA07327B-s332

RA-009-C9RA07327B-s333

RA-009-C9RA07327B-s334

RA-009-C9RA07327B-s335

RA-009-C9RA07327B-s336

RA-009-C9RA07327B-s337

RA-009-C9RA07327B-s338

RA-009-C9RA07327B-s339

RA-009-C9RA07327B-s340

RA-009-C9RA07327B-s341

RA-009-C9RA07327B-s342

RA-009-C9RA07327B-s343

RA-009-C9RA07327B-s344

RA-009-C9RA07327B-s345

RA-009-C9RA07327B-s346

RA-009-C9RA07327B-s347

RA-009-C9RA07327B-s348

RA-009-C9RA07327B-s349

RA-009-C9RA07327B-s350

RA-009-C9RA07327B-s351

RA-009-C9RA07327B-s352

RA-009-C9RA07327B-s353

RA-009-C9RA07327B-s354

RA-009-C9RA07327B-s355

RA-009-C9RA07327B-s356

RA-009-C9RA07327B-s357

RA-009-C9RA07327B-s358

RA-009-C9RA07327B-s359

RA-009-C9RA07327B-s360

RA-009-C9RA07327B-s361

RA-009-C9RA07327B-s362

RA-009-C9RA07327B-s363

RA-009-C9RA07327B-s364

RA-009-C9RA07327B-s365

RA-009-C9RA07327B-s366

RA-009-C9RA07327B-s367

RA-009-C9RA07327B-s368

RA-009-C9RA07327B-s369

RA-009-C9RA07327B-s370

RA-009-C9RA07327B-s371

RA-009-C9RA07327B-s372

RA-009-C9RA07327B-s373

RA-009-C9RA07327B-s374

RA-009-C9RA07327B-s375

RA-009-C9RA07327B-s376

RA-009-C9RA07327B-s377

RA-009-C9RA07327B-s378

RA-009-C9RA07327B-s379

RA-009-C9RA07327B-s380

RA-009-C9RA07327B-s381

RA-009-C9RA07327B-s382

RA-009-C9RA07327B-s383

RA-009-C9RA07327B-s384

RA-009-C9RA07327B-s385

RA-009-C9RA07327B-s386

RA-009-C9RA07327B-s387

RA-009-C9RA07327B-s388

RA-009-C9RA07327B-s389

RA-009-C9RA07327B-s390

RA-009-C9RA07327B-s391

RA-009-C9RA07327B-s392

RA-009-C9RA07327B-s393

RA-009-C9RA07327B-s394

RA-009-C9RA07327B-s395

RA-009-C9RA07327B-s396

RA-009-C9RA07327B-s397

RA-009-C9RA07327B-s398

RA-009-C9RA07327B-s399

RA-009-C9RA07327B-s400

RA-009-C9RA07327B-s401

RA-009-C9RA07327B-s402

RA-009-C9RA07327B-s403

RA-009-C9RA07327B-s404

RA-009-C9RA07327B-s405

RA-009-C9RA07327B-s406

RA-009-C9RA07327B-s407

RA-009-C9RA07327B-s408

RA-009-C9RA07327B-s409

RA-009-C9RA07327B-s410

RA-009-C9RA07327B-s411

RA-009-C9RA07327B-s412

RA-009-C9RA07327B-s413

RA-009-C9RA07327B-s414

RA-009-C9RA07327B-s415

RA-009-C9RA07327B-s416

RA-009-C9RA07327B-s417

RA-009-C9RA07327B-s418

RA-009-C9RA07327B-s419

RA-009-C9RA07327B-s420

RA-009-C9RA07327B-s421

RA-009-C9RA07327B-s422

RA-009-C9RA07327B-s423

RA-009-C9RA07327B-s424

RA-009-C9RA07327B-s425

RA-009-C9RA07327B-s426

RA-009-C9RA07327B-s427

RA-009-C9RA07327B-s428

RA-009-C9RA07327B-s429

RA-009-C9RA07327B-s430

RA-009-C9RA07327B-s431

RA-009-C9RA07327B-s432

RA-009-C9RA07327B-s433

RA-009-C9RA07327B-s434

RA-009-C9RA07327B-s435

RA-009-C9RA07327B-s436

RA-009-C9RA07327B-s437

RA-009-C9RA07327B-s438

RA-009-C9RA07327B-s439

RA-009-C9RA07327B-s440

RA-009-C9RA07327B-s441

RA-009-C9RA07327B-s442

RA-009-C9RA07327B-s443

RA-009-C9RA07327B-s444

RA-009-C9RA07327B-s445

RA-009-C9RA07327B-s446

RA-009-C9RA07327B-s447

RA-009-C9RA07327B-s448

RA-009-C9RA07327B-s449

RA-009-C9RA07327B-s450

RA-009-C9RA07327B-s451

RA-009-C9RA07327B-s452

RA-009-C9RA07327B-s453

RA-009-C9RA07327B-s454

RA-009-C9RA07327B-s455

RA-009-C9RA07327B-s456

RA-009-C9RA07327B-s457

RA-009-C9RA07327B-s458

RA-009-C9RA07327B-s459

RA-009-C9RA07327B-s460

RA-009-C9RA07327B-s461

RA-009-C9RA07327B-s462

RA-009-C9RA07327B-s463

RA-009-C9RA07327B-s464

RA-009-C9RA07327B-s465

RA-009-C9RA07327B-s466

RA-009-C9RA07327B-s467

RA-009-C9RA07327B-s468

RA-009-C9RA07327B-s469

RA-009-C9RA07327B-s470

RA-009-C9RA07327B-s471

RA-009-C9RA07327B-s472

RA-009-C9RA07327B-s473

RA-009-C9RA07327B-s474

RA-009-C9RA07327B-s475

RA-009-C9RA07327B-s476

RA-009-C9RA07327B-s477

RA-009-C9RA07327B-s478

RA-009-C9RA07327B-s479

RA-009-C9RA07327B-s480

RA-009-C9RA07327B-s481

RA-009-C9RA07327B-s482

RA-009-C9RA07327B-s483

RA-009-C9RA07327B-s484

RA-009-C9RA07327B-s485

RA-009-C9RA07327B-s486

RA-009-C9RA07327B-s487

RA-009-C9RA07327B-s488

RA-009-C9RA07327B-s489

RA-009-C9RA07327B-s490

RA-009-C9RA07327B-s491

RA-009-C9RA07327B-s492

RA-009-C9RA07327B-s493

RA-009-C9RA07327B-s494

RA-009-C9RA07327B-s495

RA-009-C9RA07327B-s496

RA-009-C9RA07327B-s497

RA-009-C9RA07327B-s498

RA-009-C9RA07327B-s499

RA-009-C9RA07327B-s500

RA-009-C9RA07327B-s501

RA-009-C9RA07327B-s502

RA-009-C9RA07327B-s503

RA-009-C9RA07327B-s504

RA-009-C9RA07327B-s505

RA-009-C9RA07327B-s506

RA-009-C9RA07327B-s507

RA-009-C9RA07327B-s508

RA-009-C9RA07327B-s509

RA-009-C9RA07327B-s510

RA-009-C9RA07327B-s511

RA-009-C9RA07327B-s512

RA-009-C9RA07327B-s513

RA-009-C9RA07327B-s514

RA-009-C9RA07327B-s515

RA-009-C9RA07327B-s516

RA-009-C9RA07327B-s517

RA-009-C9RA07327B-s518

RA-009-C9RA07327B-s519

RA-009-C9RA07327B-s520

RA-009-C9RA07327B-s521

RA-009-C9RA07327B-s522

RA-009-C9RA07327B-s523

RA-009-C9RA07327B-s524

RA-009-C9RA07327B-s525

RA-009-C9RA07327B-s526

RA-009-C9RA07327B-s527

RA-009-C9RA07327B-s528

RA-009-C9RA07327B-s529

RA-009-C9RA07327B-s530

RA-009-C9RA07327B-s531

RA-009-C9RA07327B-s532

RA-009-C9RA07327B-s533

RA-009-C9RA07327B-s534

RA-009-C9RA07327B-s535

RA-009-C9RA07327B-s536

RA-009-C9RA07327B-s537

RA-009-C9RA07327B-s538

RA-009-C9RA07327B-s539

RA-009-C9RA07327B-s540

RA-009-C9RA07327B-s541

RA-009-C9RA07327B-s542

RA-009-C9RA07327B-s543

RA-009-C9RA07327B-s544

RA-009-C9RA07327B-s545

RA-009-C9RA07327B-s546

RA-009-C9RA07327B-s547

RA-009-C9RA07327B-s548

RA-009-C9RA07327B-s549

RA-009-C9RA07327B-s550

RA-009-C9RA07327B-s551

RA-009-C9RA07327B-s552

RA-009-C9RA07327B-s553

RA-009-C9RA07327B-s554

RA-009-C9RA07327B-s555

RA-009-C9RA07327B-s556

RA-009-C9RA07327B-s557

RA-009-C9RA07327B-s558

RA-009-C9RA07327B-s559

RA-009-C9RA07327B-s560

RA-009-C9RA07327B-s561

RA-009-C9RA07327B-s562

RA-009-C9RA07327B-s563

RA-009-C9RA07327B-s564

RA-009-C9RA07327B-s565

RA-009-C9RA07327B-s566

RA-009-C9RA07327B-s567

RA-009-C9RA07327B-s568

RA-009-C9RA07327B-s569

RA-009-C9RA07327B-s570

RA-009-C9RA07327B-s571

RA-009-C9RA07327B-s572

RA-009-C9RA07327B-s573

RA-009-C9RA07327B-s574

RA-009-C9RA07327B-s575

RA-009-C9RA07327B-s576

RA-009-C9RA07327B-s577

RA-009-C9RA07327B-s578

RA-009-C9RA07327B-s579

RA-009-C9RA07327B-s580

RA-009-C9RA07327B-s581

RA-009-C9RA07327B-s582

RA-009-C9RA07327B-s583

RA-009-C9RA07327B-s584

RA-009-C9RA07327B-s585

RA-009-C9RA07327B-s586

RA-009-C9RA07327B-s587

RA-009-C9RA07327B-s588

RA-009-C9RA07327B-s589

RA-009-C9RA07327B-s590

RA-009-C9RA07327B-s591

RA-009-C9RA07327B-s592

RA-009-C9RA07327B-s593

RA-009-C9RA07327B-s594

RA-009-C9RA07327B-s595

RA-009-C9RA07327B-s596

RA-009-C9RA07327B-s597

RA-009-C9RA07327B-s598

RA-009-C9RA07327B-s599

RA-009-C9RA07327B-s600

RA-009-C9RA07327B-s601

RA-009-C9RA07327B-s602

RA-009-C9RA07327B-s603

RA-009-C9RA07327B-s604

RA-009-C9RA07327B-s605

RA-009-C9RA07327B-s606

RA-009-C9RA07327B-s607

RA-009-C9RA07327B-s608

RA-009-C9RA07327B-s609

RA-009-C9RA07327B-s610

RA-009-C9RA07327B-s611

RA-009-C9RA07327B-s612

RA-009-C9RA07327B-s613

RA-009-C9RA07327B-s614

RA-009-C9RA07327B-s615

RA-009-C9RA07327B-s616

RA-009-C9RA07327B-s617

RA-009-C9RA07327B-s618

RA-009-C9RA07327B-s619

RA-009-C9RA07327B-s620

RA-009-C9RA07327B-s621

RA-009-C9RA07327B-s622

RA-009-C9RA07327B-s623

RA-009-C9RA07327B-s624

RA-009-C9RA07327B-s625

RA-009-C9RA07327B-s626

RA-009-C9RA07327B-s627

RA-009-C9RA07327B-s628

RA-009-C9RA07327B-s629

RA-009-C9RA07327B-s630

RA-009-C9RA07327B-s631

RA-009-C9RA07327B-s632

RA-009-C9RA07327B-s633

RA-009-C9RA07327B-s634

RA-009-C9RA07327B-s635

RA-009-C9RA07327B-s636

RA-009-C9RA07327B-s637

RA-009-C9RA07327B-s638

RA-009-C9RA07327B-s639

RA-009-C9RA07327B-s640

RA-009-C9RA07327B-s641

RA-009-C9RA07327B-s642

RA-009-C9RA07327B-s643

RA-009-C9RA07327B-s644

RA-009-C9RA07327B-s645

RA-009-C9RA07327B-s646

RA-009-C9RA07327B-s647

RA-009-C9RA07327B-s648

RA-009-C9RA07327B-s649

RA-009-C9RA07327B-s650

RA-009-C9RA07327B-s651

RA-009-C9RA07327B-s652

RA-009-C9RA07327B-s653

RA-009-C9RA07327B-s654

RA-009-C9RA07327B-s655

RA-009-C9RA07327B-s656

RA-009-C9RA07327B-s657

RA-009-C9RA07327B-s658

RA-009-C9RA07327B-s659

RA-009-C9RA07327B-s660

RA-009-C9RA07327B-s661

RA-009-C9RA07327B-s662

RA-009-C9RA07327B-s663

RA-009-C9RA07327B-s664

RA-009-C9RA07327B-s665

RA-009-C9RA07327B-s666

RA-009-C9RA07327B-s667

RA-009-C9RA07327B-s668

RA-009-C9RA07327B-s669

RA-009-C9RA07327B-s670

RA-009-C9RA07327B-s671

RA-009-C9RA07327B-s672

RA-009-C9RA07327B-s673

RA-009-C9RA07327B-s674

RA-009-C9RA07327B-s675

RA-009-C9RA07327B-s676

RA-009-C9RA07327B-s677

RA-009-C9RA07327B-s678

RA-009-C9RA07327B-s679

RA-009-C9RA07327B-s680

RA-009-C9RA07327B-s681

RA-009-C9RA07327B-s682

RA-009-C9RA07327B-s683

RA-009-C9RA07327B-s684

RA-009-C9RA07327B-s685

RA-009-C9RA07327B-s686

RA-009-C9RA07327B-s687

RA-009-C9RA07327B-s688

RA-009-C9RA07327B-s689

RA-009-C9RA07327B-s690

RA-009-C9RA07327B-s691

RA-009-C9RA07327B-s692

RA-009-C9RA07327B-s693

RA-009-C9RA07327B-s694

RA-009-C9RA07327B-s695

RA-009-C9RA07327B-s696

RA-009-C9RA07327B-s697

RA-009-C9RA07327B-s698

RA-009-C9RA07327B-s699

RA-009-C9RA07327B-s700

RA-009-C9RA07327B-s701

RA-009-C9RA07327B-s702

RA-009-C9RA07327B-s703

RA-009-C9RA07327B-s704

RA-009-C9RA07327B-s705

RA-009-C9RA07327B-s706

RA-009-C9RA07327B-s707

RA-009-C9RA07327B-s708

RA-009-C9RA07327B-s709

RA-009-C9RA07327B-s710

RA-009-C9RA07327B-s711

RA-009-C9RA07327B-s712

RA-009-C9RA07327B-s713

RA-009-C9RA07327B-s714

RA-009-C9RA07327B-s715

RA-009-C9RA07327B-s716

RA-009-C9RA07327B-s717

RA-009-C9RA07327B-s718

RA-009-C9RA07327B-s719

RA-009-C9RA07327B-s720

RA-009-C9RA07327B-s721

RA-009-C9RA07327B-s722

RA-009-C9RA07327B-s723

RA-009-C9RA07327B-s724

RA-009-C9RA07327B-s725

RA-009-C9RA07327B-s726

RA-009-C9RA07327B-s727

RA-009-C9RA07327B-s728

RA-009-C9RA07327B-s729

RA-009-C9RA07327B-s730

RA-009-C9RA07327B-s731

RA-009-C9RA07327B-s732

RA-009-C9RA07327B-s733

RA-009-C9RA07327B-s734

RA-009-C9RA07327B-s735

RA-009-C9RA07327B-s736

RA-009-C9RA07327B-s737

RA-009-C9RA07327B-s738

RA-009-C9RA07327B-s739

RA-009-C9RA07327B-s740

RA-009-C9RA07327B-s741

RA-009-C9RA07327B-s742

RA-009-C9RA07327B-s743

RA-009-C9RA07327B-s744

RA-009-C9RA07327B-s745

RA-009-C9RA07327B-s746

RA-009-C9RA07327B-s747

RA-009-C9RA07327B-s748

RA-009-C9RA07327B-s749

RA-009-C9RA07327B-s750

RA-009-C9RA07327B-s751

RA-009-C9RA07327B-s752

RA-009-C9RA07327B-s753

RA-009-C9RA07327B-s754

RA-009-C9RA07327B-s755

RA-009-C9RA07327B-s756

RA-009-C9RA07327B-s757

RA-009-C9RA07327B-s758

RA-009-C9RA07327B-s759

RA-009-C9RA07327B-s760

RA-009-C9RA07327B-s761

RA-009-C9RA07327B-s762

RA-009-C9RA07327B-s763

RA-009-C9RA07327B-s764

RA-009-C9RA07327B-s765

RA-009-C9RA07327B-s766

RA-009-C9RA07327B-s767

RA-009-C9RA07327B-s768

RA-009-C9RA07327B-s769

RA-009-C9RA07327B-s770

RA-009-C9RA07327B-s771

RA-009-C9RA07327B-s772

RA-009-C9RA07327B-s773

RA-009-C9RA07327B-s774

RA-009-C9RA07327B-s775

RA-009-C9RA07327B-s776

RA-009-C9RA07327B-s777

RA-009-C9RA07327B-s778

RA-009-C9RA07327B-s779

RA-009-C9RA07327B-s780

RA-009-C9RA07327B-s781

RA-009-C9RA07327B-s782

RA-009-C9RA07327B-s783

RA-009-C9RA07327B-s784

RA-009-C9RA07327B-s785

RA-009-C9RA07327B-s786

RA-009-C9RA07327B-s787

RA-009-C9RA07327B-s788

RA-009-C9RA07327B-s789

RA-009-C9RA07327B-s790

RA-009-C9RA07327B-s791

RA-009-C9RA07327B-s792

RA-009-C9RA07327B-s793

RA-009-C9RA07327B-s794

RA-009-C9RA07327B-s795

RA-009-C9RA07327B-s796

RA-009-C9RA07327B-s797

RA-009-C9RA07327B-s798

RA-009-C9RA07327B-s799

RA-009-C9RA07327B-s800

RA-009-C9RA07327B-s801

RA-009-C9RA07327B-s802

RA-009-C9RA07327B-s803

RA-009-C9RA07327B-s804

RA-009-C9RA07327B-s805

RA-009-C9RA07327B-s806

RA-009-C9RA07327B-s807

RA-009-C9RA07327B-s808

RA-009-C9RA07327B-s809

RA-009-C9RA07327B-s810

RA-009-C9RA07327B-s811

RA-009-C9RA07327B-s812

RA-009-C9RA07327B-s813

RA-009-C9RA07327B-s814

RA-009-C9RA07327B-s815

RA-009-C9RA07327B-s816

RA-009-C9RA07327B-s817

RA-009-C9RA07327B-s818

RA-009-C9RA07327B-s819

RA-009-C9RA07327B-s820

RA-009-C9RA07327B-s821

RA-009-C9RA07327B-s822

RA-009-C9RA07327B-s823

RA-009-C9RA07327B-s824

RA-009-C9RA07327B-s825

RA-009-C9RA07327B-s826

RA-009-C9RA07327B-s827

RA-009-C9RA07327B-s828

RA-009-C9RA07327B-s829

RA-009-C9RA07327B-s830

RA-009-C9RA07327B-s831

RA-009-C9RA07327B-s832

RA-009-C9RA07327B-s833

RA-009-C9RA07327B-s834

RA-009-C9RA07327B-s835

RA-009-C9RA07327B-s836

RA-009-C9RA07327B-s837

RA-009-C9RA07327B-s838

RA-009-C9RA07327B-s839

RA-009-C9RA07327B-s840

RA-009-C9RA07327B-s841

RA-009-C9RA07327B-s842

RA-009-C9RA07327B-s843

RA-009-C9RA07327B-s844

RA-009-C9RA07327B-s845

RA-009-C9RA07327B-s846

RA-009-C9RA07327B-s847

RA-009-C9RA07327B-s848

RA-009-C9RA07327B-s849

RA-009-C9RA07327B-s850

RA-009-C9RA07327B-s851

RA-009-C9RA07327B-s852

RA-009-C9RA07327B-s853

RA-009-C9RA07327B-s854

RA-009-C9RA07327B-s855

RA-009-C9RA07327B-s856

RA-009-C9RA07327B-s857

RA-009-C9RA07327B-s858

RA-009-C9RA07327B-s859

RA-009-C9RA07327B-s860

RA-009-C9RA07327B-s861

RA-009-C9RA07327B-s862

RA-009-C9RA07327B-s863

RA-009-C9RA07327B-s864

RA-009-C9RA07327B-s865

RA-009-C9RA07327B-s866

RA-009-C9RA07327B-s867

RA-009-C9RA07327B-s868

RA-009-C9RA07327B-s869

RA-009-C9RA07327B-s870

RA-009-C9RA07327B-s871

RA-009-C9RA07327B-s872

RA-009-C9RA07327B-s873

RA-009-C9RA07327B-s874

RA-009-C9RA07327B-s875

RA-009-C9RA07327B-s876

RA-009-C9RA07327B-s877

RA-009-C9RA07327B-s878

RA-009-C9RA07327B-s879

RA-009-C9RA07327B-s880

RA-009-C9RA07327B-s881

RA-009-C9RA07327B-s882

RA-009-C9RA07327B-s883

RA-009-C9RA07327B-s884

RA-009-C9RA07327B-s885

RA-009-C9RA07327B-s886

RA-009-C9RA07327B-s887

RA-009-C9RA07327B-s888

RA-009-C9RA07327B-s889

RA-009-C9RA07327B-s890

RA-009-C9RA07327B-s891

RA-009-C9RA07327B-s892

RA-009-C9RA07327B-s893

RA-009-C9RA07327B-s894

RA-009-C9RA07327B-s895

RA-009-C9RA07327B-s896

RA-009-C9RA07327B-s897

RA-009-C9RA07327B-s898

RA-009-C9RA07327B-s899

RA-009-C9RA07327B-s900

RA-009-C9RA07327B-s901

RA-009-C9RA07327B-s902

RA-009-C9RA07327B-s903

RA-009-C9RA07327B-s904

RA-009-C9RA07327B-s905

RA-009-C9RA07327B-s906

RA-009-C9RA07327B-s907

RA-009-C9RA07327B-s908

RA-009-C9RA07327B-s909

RA-009-C9RA07327B-s910

RA-009-C9RA07327B-s911

RA-009-C9RA07327B-s912

RA-009-C9RA07327B-s913

RA-009-C9RA07327B-s914

RA-009-C9RA07327B-s915

RA-009-C9RA07327B-s916

RA-009-C9RA07327B-s917

RA-009-C9RA07327B-s918

RA-009-C9RA07327B-s919

RA-009-C9RA07327B-s920

RA-009-C9RA07327B-s921

RA-009-C9RA07327B-s922

RA-009-C9RA07327B-s923

RA-009-C9RA07327B-s924

RA-009-C9RA07327B-s925

RA-009-C9RA07327B-s926

RA-009-C9RA07327B-s927

RA-009-C9RA07327B-s928

RA-009-C9RA07327B-s929

RA-009-C9RA07327B-s930

RA-009-C9RA07327B-s931

RA-009-C9RA07327B-s932

RA-009-C9RA07327B-s933

RA-009-C9RA07327B-s934

RA-009-C9RA07327B-s935

RA-009-C9RA07327B-s936

RA-009-C9RA07327B-s937

RA-009-C9RA07327B-s938

RA-009-C9RA07327B-s939

RA-009-C9RA07327B-s940

RA-009-C9RA07327B-s941

RA-009-C9RA07327B-s942

RA-009-C9RA07327B-s943

RA-009-C9RA07327B-s944

RA-009-C9RA07327B-s945

RA-009-C9RA07327B-s946

RA-009-C9RA07327B-s947

RA-009-C9RA07327B-s948

RA-009-C9RA07327B-s949

RA-009-C9RA07327B-s950

RA-009-C9RA07327B-s951

RA-009-C9RA07327B-s952

RA-009-C9RA07327B-s953

RA-009-C9RA07327B-s954

RA-009-C9RA07327B-s955

RA-009-C9RA07327B-s956

RA-009-C9RA07327B-s957

RA-009-C9RA07327B-s958

RA-009-C9RA07327B-s959

RA-009-C9RA07327B-s960

RA-009-C9RA07327B-s961

RA-009-C9RA07327B-s962

RA-009-C9RA07327B-s963

RA-009-C9RA07327B-s964

RA-009-C9RA07327B-s965

RA-009-C9RA07327B-s966

RA-009-C9RA07327B-s967

RA-009-C9RA07327B-s968

RA-009-C9RA07327B-s969

RA-009-C9RA07327B-s970

RA-009-C9RA07327B-s971

RA-009-C9RA07327B-s972

RA-009-C9RA07327B-s973

RA-009-C9RA07327B-s974

RA-009-C9RA07327B-s975

RA-009-C9RA07327B-s976

RA-009-C9RA07327B-s977

RA-009-C9RA07327B-s978

RA-009-C9RA07327B-s979

RA-009-C9RA07327B-s980

RA-009-C9RA07327B-s981

RA-009-C9RA07327B-s982

RA-009-C9RA07327B-s983

RA-009-C9RA07327B-s984

RA-009-C9RA07327B-s985

RA-009-C9RA07327B-s986

RA-009-C9RA07327B-s987

RA-009-C9RA07327B-s988

RA-009-C9RA07327B-s989

RA-009-C9RA07327B-s990

RA-009-C9RA07327B-s991

RA-009-C9RA07327B-s992

RA-009-C9RA07327B-s993

RA-009-C9RA07327B-s994

RA-009-C9RA07327B-s995

RA-009-C9RA07327B-s996

RA-009-C9RA07327B-s997

RA-009-C9RA07327B-s998

RA-009-C9RA07327B-s999

RA-009-C9RA07327B-s1000

RA-009-C9RA07327B-s1001

RA-009-C9RA07327B-s1002

RA-009-C9RA07327B-s1003

RA-009-C9RA07327B-s1004

RA-009-C9RA07327B-s1005

RA-009-C9RA07327B-s1006

RA-009-C9RA07327B-s1007

RA-009-C9RA07327B-s1008

RA-009-C9RA07327B-s1009

RA-009-C9RA07327B-s1010

RA-009-C9RA07327B-s1011

RA-009-C9RA07327B-s1012

RA-009-C9RA07327B-s1013

RA-009-C9RA07327B-s1014

RA-009-C9RA07327B-s1015

RA-009-C9RA07327B-s1016

RA-009-C9RA07327B-s1017

RA-009-C9RA07327B-s1018

RA-009-C9RA07327B-s1019

RA-009-C9RA07327B-s1020

RA-009-C9RA07327B-s1021

RA-009-C9RA07327B-s1022

RA-009-C9RA07327B-s1023

RA-009-C9RA07327B-s1024

RA-009-C9RA07327B-s1025

RA-009-C9RA07327B-s1026

RA-009-C9RA07327B-s1027

RA-009-C9RA07327B-s1028

RA-009-C9RA07327B-s1029

RA-009-C9RA07327B-s1030

RA-009-C9RA07327B-s1031

RA-009-C9RA07327B-s1032

RA-009-C9RA07327B-s1033

RA-009-C9RA07327B-s1034

RA-009-C9RA07327B-s1035

RA-009-C9RA07327B-s1036

RA-009-C9RA07327B-s1037

RA-009-C9RA07327B-s1038

RA-009-C9RA07327B-s1039

RA-009-C9RA07327B-s1040

RA-009-C9RA07327B-s1041

RA-009-C9RA07327B-s1042

RA-009-C9RA07327B-s1043

RA-009-C9RA07327B-s1044

RA-009-C9RA07327B-s1045

RA-009-C9RA07327B-s1046

RA-009-C9RA07327B-s1047

RA-009-C9RA07327B-s1048

RA-009-C9RA07327B-s1049

RA-009-C9RA07327B-s1050

RA-009-C9RA07327B-s1051

RA-009-C9RA07327B-s1052

RA-009-C9RA07327B-s1053

RA-009-C9RA07327B-s1054

RA-009-C9RA07327B-s1055

RA-009-C9RA07327B-s1056

RA-009-C9RA07327B-s1057

RA-009-C9RA07327B-s1058

RA-009-C9RA07327B-s1059

RA-009-C9RA07327B-s1060

RA-009-C9RA07327B-s1061

RA-009-C9RA07327B-s1062

RA-009-C9RA07327B-s1063

RA-009-C9RA07327B-s1064

RA-009-C9RA07327B-s1065

RA-009-C9RA07327B-s1066

RA-009-C9RA07327B-s1067

RA-009-C9RA07327B-s1068

RA-009-C9RA07327B-s1069

RA-009-C9RA07327B-s1070

RA-009-C9RA07327B-s1071

RA-009-C9RA07327B-s1072

RA-009-C9RA07327B-s1073

RA-009-C9RA07327B-s1074

RA-009-C9RA07327B-s1075

RA-009-C9RA07327B-s1076

RA-009-C9RA07327B-s1077

RA-009-C9RA07327B-s1078

RA-009-C9RA07327B-s1079

RA-009-C9RA07327B-s1080

RA-009-C9RA07327B-s1081

RA-009-C9RA07327B-s1082

RA-009-C9RA07327B-s1083

RA-009-C9RA07327B-s1084

RA-009-C9RA07327B-s1085

RA-009-C9RA07327B-s1086

RA-009-C9RA07327B-s1087

RA-009-C9RA07327B-s1088

RA-009-C9RA07327B-s1089

RA-009-C9RA07327B-s1090

RA-009-C9RA07327B-s1091

RA-009-C9RA07327B-s1092

RA-009-C9RA07327B-s1093

RA-009-C9RA07327B-s1094

RA-009-C9RA07327B-s1095

RA-009-C9RA07327B-s1096

RA-009-C9RA07327B-s1097

RA-009-C9RA07327B-s1098

RA-009-C9RA07327B-s1099

RA-009-C9RA07327B-s1100

RA-009-C9RA07327B-s1101

RA-009-C9RA07327B-s1102

RA-009-C9RA07327B-s1103

RA-009-C9RA07327B-s1104

RA-009-C9RA07327B-s1105

RA-009-C9RA07327B-s1106

RA-009-C9RA07327B-s1107

RA-009-C9RA07327B-s1108

RA-009-C9RA07327B-s1109

RA-009-C9RA07327B-s1110

RA-009-C9RA07327B-s1111

RA-009-C9RA07327B-s1112

RA-009-C9RA07327B-s1113

RA-009-C9RA07327B-s1114

RA-009-C9RA07327B-s1115

RA-009-C9RA07327B-s1116

RA-009-C9RA07327B-s1117

RA-009-C9RA07327B-s1118

RA-009-C9RA07327B-s1119

RA-009-C9RA07327B-s1120

RA-009-C9RA07327B-s1121

RA-009-C9RA07327B-s1122

RA-009-C9RA07327B-s1123

RA-009-C9RA07327B-s1124

RA-009-C9RA07327B-s1125

RA-009-C9RA07327B-s1126

RA-009-C9RA07327B-s1127

RA-009-C9RA07327B-s1128

RA-009-C9RA07327B-s1129

RA-009-C9RA07327B-s1130

RA-009-C9RA07327B-s1131

RA-009-C9RA07327B-s1132

RA-009-C9RA07327B-s1133

RA-009-C9RA07327B-s1134

RA-009-C9RA07327B-s1135

RA-009-C9RA07327B-s1136

RA-009-C9RA07327B-s1137

RA-009-C9RA07327B-s1138

RA-009-C9RA07327B-s1139

RA-009-C9RA07327B-s1140

RA-009-C9RA07327B-s1141

RA-009-C9RA07327B-s1142

RA-009-C9RA07327B-s1143

RA-009-C9RA07327B-s1144

RA-009-C9RA07327B-s1145

RA-009-C9RA07327B-s1146

RA-009-C9RA07327B-s1147

RA-009-C9RA07327B-s1148

RA-009-C9RA07327B-s1149

RA-009-C9RA07327B-s1150

RA-009-C9RA07327B-s1151

RA-009-C9RA07327B-s1152

RA-009-C9RA07327B-s1153

RA-009-C9RA07327B-s1154

RA-009-C9RA07327B-s1155

RA-009-C9RA07327B-s1156

RA-009-C9RA07327B-s1157

RA-009-C9RA07327B-s1158

RA-009-C9RA07327B-s1159

RA-009-C9RA07327B-s1160

RA-009-C9RA07327B-s1161

RA-009-C9RA07327B-s1162

RA-009-C9RA07327B-s1163

RA-009-C9RA07327B-s1164

RA-009-C9RA07327B-s1165

RA-009-C9RA07327B-s1166

RA-009-C9RA07327B-s1167

RA-009-C9RA07327B-s1168

RA-009-C9RA07327B-s1169

RA-009-C9RA07327B-s1170

RA-009-C9RA07327B-s1171

RA-009-C9RA07327B-s1172

RA-009-C9RA07327B-s1173

RA-009-C9RA07327B-s1174

RA-009-C9RA07327B-s1175

RA-009-C9RA07327B-s1176

RA-009-C9RA07327B-s1177

RA-009-C9RA07327B-s1178

RA-009-C9RA07327B-s1179

RA-009-C9RA07327B-s1180

RA-009-C9RA07327B-s1181

RA-009-C9RA07327B-s1182

RA-009-C9RA07327B-s1183

RA-009-C9RA07327B-s1184

RA-009-C9RA07327B-s1185

RA-009-C9RA07327B-s1186

RA-009-C9RA07327B-s1187

RA-009-C9RA07327B-s1188

RA-009-C9RA07327B-s1189

RA-009-C9RA07327B-s1190

RA-009-C9RA07327B-s1191

RA-009-C9RA07327B-s1192

RA-009-C9RA07327B-s1193

RA-009-C9RA07327B-s1194

RA-009-C9RA07327B-s1195

RA-009-C9RA07327B-s1196

RA-009-C9RA07327B-s1197

RA-009-C9RA07327B-s1198

RA-009-C9RA07327B-s1199

RA-009-C9RA07327B-s1200

RA-009-C9RA07327B-s1201

RA-009-C9RA07327B-s1202

RA-009-C9RA07327B-s1203

RA-009-C9RA07327B-s1204

RA-009-C9RA07327B-s1205

RA-009-C9RA07327B-s1206

RA-009-C9RA07327B-s1207

RA-009-C9RA07327B-s1208

RA-009-C9RA07327B-s1209

RA-009-C9RA07327B-s1210

RA-009-C9RA07327B-s1211

RA-009-C9RA07327B-s1212

RA-009-C9RA07327B-s1213

RA-009-C9RA07327B-s1214

RA-009-C9RA07327B-s1215

RA-009-C9RA07327B-s1216

RA-009-C9RA07327B-s1217

RA-009-C9RA07327B-s1218

RA-009-C9RA07327B-s1219

RA-009-C9RA07327B-s1220

RA-009-C9RA07327B-s1221

RA-009-C9RA07327B-s1222

RA-009-C9RA07327B-s1223

RA-009-C9RA07327B-s1224

RA-009-C9RA07327B-s1225

RA-009-C9RA07327B-s1226

RA-009-C9RA07327B-s1227

RA-009-C9RA07327B-s1228

RA-009-C9RA07327B-s1229

RA-009-C9RA07327B-s1230

RA-009-C9RA07327B-s1231

RA-009-C9RA07327B-s1232

RA-009-C9RA07327B-s1233

RA-009-C9RA07327B-s1234

RA-009-C9RA07327B-s1235

RA-009-C9RA07327B-s1236

RA-009-C9RA07327B-s1237

RA-009-C9RA07327B-s1238

RA-009-C9RA07327B-s1239

RA-009-C9RA07327B-s1240

RA-009-C9RA07327B-s1241

RA-009-C9RA07327B-s1242

RA-009-C9RA07327B-s1243

RA-009-C9RA07327B-s1244

RA-009-C9RA07327B-s1245

RA-009-C9RA07327B-s1246

RA-009-C9RA07327B-s1247

RA-009-C9RA07327B-s1248

RA-009-C9RA07327B-s1249

RA-009-C9RA07327B-s1250

RA-009-C9RA07327B-s1251

RA-009-C9RA07327B-s1252

RA-009-C9RA07327B-s1253

RA-009-C9RA07327B-s1254

RA-009-C9RA07327B-s1255

RA-009-C9RA07327B-s1256

RA-009-C9RA07327B-s1257

RA-009-C9RA07327B-s1258

RA-009-C9RA07327B-s1259

RA-009-C9RA07327B-s1260

RA-009-C9RA07327B-s1261

RA-009-C9RA07327B-s1262

RA-009-C9RA07327B-s1263

RA-009-C9RA07327B-s1264

RA-009-C9RA07327B-s1265

RA-009-C9RA07327B-s1266

RA-009-C9RA07327B-s1267

RA-009-C9RA07327B-s1268

RA-009-C9RA07327B-s1269

RA-009-C9RA07327B-s1270

RA-009-C9RA07327B-s1271

RA-009-C9RA07327B-s1272

RA-009-C9RA07327B-s1273

RA-009-C9RA07327B-s1274

RA-009-C9RA07327B-s1275

RA-009-C9RA07327B-s1276

RA-009-C9RA07327B-s1277

RA-009-C9RA07327B-s1278

RA-009-C9RA07327B-s1279

RA-009-C9RA07327B-s1280

RA-009-C9RA07327B-s1281

RA-009-C9RA07327B-s1282

RA-009-C9RA07327B-s1283

RA-009-C9RA07327B-s1284

RA-009-C9RA07327B-s1285

RA-009-C9RA07327B-s1286

RA-009-C9RA07327B-s1287

RA-009-C9RA07327B-s1288

RA-009-C9RA07327B-s1289

RA-009-C9RA07327B-s1290

RA-009-C9RA07327B-s1291

RA-009-C9RA07327B-s1292

RA-009-C9RA07327B-s1293

RA-009-C9RA07327B-s1294

RA-009-C9RA07327B-s1295

RA-009-C9RA07327B-s1296

RA-009-C9RA07327B-s1297

RA-009-C9RA07327B-s1298

RA-009-C9RA07327B-s1299

RA-009-C9RA07327B-s1300

RA-009-C9RA07327B-s1301

RA-009-C9RA07327B-s1302

RA-009-C9RA07327B-s1303

RA-009-C9RA07327B-s1304

RA-009-C9RA07327B-s1305

RA-009-C9RA07327B-s1306

RA-009-C9RA07327B-s1307

RA-009-C9RA07327B-s1308

RA-009-C9RA07327B-s1309

RA-009-C9RA07327B-s1310

RA-009-C9RA07327B-s1311

RA-009-C9RA07327B-s1312

RA-009-C9RA07327B-s1313

RA-009-C9RA07327B-s1314

RA-009-C9RA07327B-s1315

RA-009-C9RA07327B-s1316

RA-009-C9RA07327B-s1317

RA-009-C9RA07327B-s1318

RA-009-C9RA07327B-s1319

RA-009-C9RA07327B-s1320

RA-009-C9RA07327B-s1321

RA-009-C9RA07327B-s1322

RA-009-C9RA07327B-s1323

RA-009-C9RA07327B-s1324

RA-009-C9RA07327B-s1325

RA-009-C9RA07327B-s1326

RA-009-C9RA07327B-s1327

RA-009-C9RA07327B-s1328

RA-009-C9RA07327B-s1329

RA-009-C9RA07327B-s1330

RA-009-C9RA07327B-s1331

RA-009-C9RA07327B-s1332

RA-009-C9RA07327B-s1333

RA-009-C9RA07327B-s1334

RA-009-C9RA07327B-s1335

RA-009-C9RA07327B-s1336

RA-009-C9RA07327B-s1337

RA-009-C9RA07327B-s1338

RA-009-C9RA07327B-s1339

RA-009-C9RA07327B-s1340

RA-009-C9RA07327B-s1341

RA-009-C9RA07327B-s1342

RA-009-C9RA07327B-s1343

RA-009-C9RA07327B-s1344

RA-009-C9RA07327B-s1345

RA-009-C9RA07327B-s1346

RA-009-C9RA07327B-s1347

RA-009-C9RA07327B-s1348

RA-009-C9RA07327B-s1349

RA-009-C9RA07327B-s1350

RA-009-C9RA07327B-s1351

RA-009-C9RA07327B-s1352

RA-009-C9RA07327B-s1353

RA-009-C9RA07327B-s1354

RA-009-C9RA07327B-s1355

RA-009-C9RA07327B-s1356

RA-009-C9RA07327B-s1357

RA-009-C9RA07327B-s1358

RA-009-C9RA07327B-s1359

RA-009-C9RA07327B-s1360

RA-009-C9RA07327B-s1361

RA-009-C9RA07327B-s1362

RA-009-C9RA07327B-s1363

RA-009-C9RA07327B-s1364

RA-009-C9RA07327B-s1365

RA-009-C9RA07327B-s1366

RA-009-C9RA07327B-s1367

RA-009-C9RA07327B-s1368

RA-009-C9RA07327B-s1369

RA-009-C9RA07327B-s1370

RA-009-C9RA07327B-s1371

RA-009-C9RA07327B-s1372

RA-009-C9RA07327B-s1373

RA-009-C9RA07327B-s1374

RA-009-C9RA07327B-s1375

RA-009-C9RA07327B-s1376

RA-009-C9RA07327B-s1377

RA-009-C9RA07327B-s1378

RA-009-C9RA07327B-s1379

RA-009-C9RA07327B-s1380

RA-009-C9RA07327B-s1381

RA-009-C9RA07327B-s1382

RA-009-C9RA07327B-s1383

RA-009-C9RA07327B-s1384

RA-009-C9RA07327B-s1385

RA-009-C9RA07327B-s1386

RA-009-C9RA07327B-s1387

RA-009-C9RA07327B-s1388

RA-009-C9RA07327B-s1389

RA-009-C9RA07327B-s1390

RA-009-C9RA07327B-s1391

RA-009-C9RA07327B-s1392

RA-009-C9RA07327B-s1393

RA-009-C9RA07327B-s1394

RA-009-C9RA07327B-s1395

RA-009-C9RA07327B-s1396

RA-009-C9RA07327B-s1397

RA-009-C9RA07327B-s1398

RA-009-C9RA07327B-s1399

RA-009-C9RA07327B-s1400

RA-009-C9RA07327B-s1401

RA-009-C9RA07327B-s1402

RA-009-C9RA07327B-s1403

RA-009-C9RA07327B-s1404

RA-009-C9RA07327B-s1405

RA-009-C9RA07327B-s1406

RA-009-C9RA07327B-s1407

RA-009-C9RA07327B-s1408

RA-009-C9RA07327B-s1409

RA-009-C9RA07327B-s1410

RA-009-C9RA07327B-s1411

RA-009-C9RA07327B-s1412

RA-009-C9RA07327B-s1413

RA-009-C9RA07327B-s1414

RA-009-C9RA07327B-s1415

RA-009-C9RA07327B-s1416

RA-009-C9RA07327B-s1417

RA-009-C9RA07327B-s1418

RA-009-C9RA07327B-s1419

RA-009-C9RA07327B-s1420

RA-009-C9RA07327B-s1421

RA-009-C9RA07327B-s1422

RA-009-C9RA07327B-s1423

RA-009-C9RA07327B-s1424

RA-009-C9RA07327B-s1425

RA-009-C9RA07327B-s1426

RA-009-C9RA07327B-s1427

RA-009-C9RA07327B-s1428

RA-009-C9RA07327B-s1429

RA-009-C9RA07327B-s1430

RA-009-C9RA07327B-s1431

RA-009-C9RA07327B-s1432

RA-009-C9RA07327B-s1433

RA-009-C9RA07327B-s1434

RA-009-C9RA07327B-s1435

RA-009-C9RA07327B-s1436

RA-009-C9RA07327B-s1437

RA-009-C9RA07327B-s1438

RA-009-C9RA07327B-s1439

RA-009-C9RA07327B-s1440

RA-009-C9RA07327B-s1441

RA-009-C9RA07327B-s1442

RA-009-C9RA07327B-s1443

RA-009-C9RA07327B-s1444

RA-009-C9RA07327B-s1445

RA-009-C9RA07327B-s1446

RA-009-C9RA07327B-s1447

RA-009-C9RA07327B-s1448

RA-009-C9RA07327B-s1449

RA-009-C9RA07327B-s1450

RA-009-C9RA07327B-s1451

RA-009-C9RA07327B-s1452

RA-009-C9RA07327B-s1453

RA-009-C9RA07327B-s1454

RA-009-C9RA07327B-s1455

RA-009-C9RA07327B-s1456

RA-009-C9RA07327B-s1457

RA-009-C9RA07327B-s1458

RA-009-C9RA07327B-s1459

RA-009-C9RA07327B-s1460

RA-009-C9RA07327B-s1461

RA-009-C9RA07327B-s1462

RA-009-C9RA07327B-s1463

RA-009-C9RA07327B-s1464

RA-009-C9RA07327B-s1465

RA-009-C9RA07327B-s1466

RA-009-C9RA07327B-s1467

RA-009-C9RA07327B-s1468

RA-009-C9RA07327B-s1469

RA-009-C9RA07327B-s1470

RA-009-C9RA07327B-s1471

RA-009-C9RA07327B-s1472

RA-009-C9RA07327B-s1473

RA-009-C9RA07327B-s1474

RA-009-C9RA07327B-s1475

RA-009-C9RA07327B-s1476

RA-009-C9RA07327B-s1477

RA-009-C9RA07327B-s1478

RA-009-C9RA07327B-s1479

RA-009-C9RA07327B-s1480

RA-009-C9RA07327B-s1481

RA-009-C9RA07327B-s1482

RA-009-C9RA07327B-s1483

RA-009-C9RA07327B-s1484

RA-009-C9RA07327B-s1485

RA-009-C9RA07327B-s1486

RA-009-C9RA07327B-s1487

RA-009-C9RA07327B-s1488

RA-009-C9RA07327B-s1489

RA-009-C9RA07327B-s1490

RA-009-C9RA07327B-s1491

RA-009-C9RA07327B-s1492

RA-009-C9RA07327B-s1493

RA-009-C9RA07327B-s1494

RA-009-C9RA07327B-s1495

RA-009-C9RA07327B-s1496

RA-009-C9RA07327B-s1497

RA-009-C9RA07327B-s1498

RA-009-C9RA07327B-s1499

RA-009-C9RA07327B-s1500

RA-009-C9RA07327B-s1501

RA-009-C9RA07327B-s1502

RA-009-C9RA07327B-s1503

RA-009-C9RA07327B-s1504

RA-009-C9RA07327B-s1505

RA-009-C9RA07327B-s1506

RA-009-C9RA07327B-s1507

RA-009-C9RA07327B-s1508

RA-009-C9RA07327B-s1509

RA-009-C9RA07327B-s1510

RA-009-C9RA07327B-s1511

RA-009-C9RA07327B-s1512

RA-009-C9RA07327B-s1513

RA-009-C9RA07327B-s1514

RA-009-C9RA07327B-s1515

RA-009-C9RA07327B-s1516

RA-009-C9RA07327B-s1517

RA-009-C9RA07327B-s1518

RA-009-C9RA07327B-s1519

RA-009-C9RA07327B-s1520

RA-009-C9RA07327B-s1521

RA-009-C9RA07327B-s1522

RA-009-C9RA07327B-s1523

RA-009-C9RA07327B-s1524

RA-009-C9RA07327B-s1525

RA-009-C9RA07327B-s1526

RA-009-C9RA07327B-s1527

RA-009-C9RA07327B-s1528

RA-009-C9RA07327B-s1529

RA-009-C9RA07327B-s1530

RA-009-C9RA07327B-s1531

RA-009-C9RA07327B-s1532

RA-009-C9RA07327B-s1533

RA-009-C9RA07327B-s1534

RA-009-C9RA07327B-s1535

RA-009-C9RA07327B-s1536

RA-009-C9RA07327B-s1537

RA-009-C9RA07327B-s1538

RA-009-C9RA07327B-s1539

RA-009-C9RA07327B-s1540

RA-009-C9RA07327B-s1541

RA-009-C9RA07327B-s1542

RA-009-C9RA07327B-s1543

RA-009-C9RA07327B-s1544

RA-009-C9RA07327B-s1545

RA-009-C9RA07327B-s1546

RA-009-C9RA07327B-s1547

RA-009-C9RA07327B-s1548

RA-009-C9RA07327B-s1549

RA-009-C9RA07327B-s1550

RA-009-C9RA07327B-s1551

RA-009-C9RA07327B-s1552

RA-009-C9RA07327B-s1553

RA-009-C9RA07327B-s1554

RA-009-C9RA07327B-s1555

RA-009-C9RA07327B-s1556

RA-009-C9RA07327B-s1557

RA-009-C9RA07327B-s1558

RA-009-C9RA07327B-s1559

RA-009-C9RA07327B-s1560

RA-009-C9RA07327B-s1561

RA-009-C9RA07327B-s1562

RA-009-C9RA07327B-s1563

RA-009-C9RA07327B-s1564

RA-009-C9RA07327B-s1565

RA-009-C9RA07327B-s1566

RA-009-C9RA07327B-s1567

RA-009-C9RA07327B-s1568

RA-009-C9RA07327B-s1569

RA-009-C9RA07327B-s1570

RA-009-C9RA07327B-s1571

RA-009-C9RA07327B-s1572

RA-009-C9RA07327B-s1573

RA-009-C9RA07327B-s1574

RA-009-C9RA07327B-s1575

RA-009-C9RA07327B-s1576

RA-009-C9RA07327B-s1577

RA-009-C9RA07327B-s1578

RA-009-C9RA07327B-s1579

RA-009-C9RA07327B-s1580

RA-009-C9RA07327B-s1581

RA-009-C9RA07327B-s1582

RA-009-C9RA07327B-s1583

RA-009-C9RA07327B-s1584

RA-009-C9RA07327B-s1585

RA-009-C9RA07327B-s1586

RA-009-C9RA07327B-s1587

RA-009-C9RA07327B-s1588

RA-009-C9RA07327B-s1589

RA-009-C9RA07327B-s1590

RA-009-C9RA07327B-s1591

RA-009-C9RA07327B-s1592

RA-009-C9RA07327B-s1593

RA-009-C9RA07327B-s1594

RA-009-C9RA07327B-s1595

RA-009-C9RA07327B-s1596

RA-009-C9RA07327B-s1597

RA-009-C9RA07327B-s1598

RA-009-C9RA07327B-s1599

RA-009-C9RA07327B-s1600

RA-009-C9RA07327B-s1601

RA-009-C9RA07327B-s1602

RA-009-C9RA07327B-s1603

RA-009-C9RA07327B-s1604

RA-009-C9RA07327B-s1605

RA-009-C9RA07327B-s1606

RA-009-C9RA07327B-s1607

RA-009-C9RA07327B-s1608

RA-009-C9RA07327B-s1609

RA-009-C9RA07327B-s1610

RA-009-C9RA07327B-s1611

RA-009-C9RA07327B-s1612

RA-009-C9RA07327B-s1613

RA-009-C9RA07327B-s1614

RA-009-C9RA07327B-s1615

RA-009-C9RA07327B-s1616

RA-009-C9RA07327B-s1617

RA-009-C9RA07327B-s1618

RA-009-C9RA07327B-s1619

RA-009-C9RA07327B-s1620

RA-009-C9RA07327B-s1621

RA-009-C9RA07327B-s1622

RA-009-C9RA07327B-s1623

RA-009-C9RA07327B-s1624

RA-009-C9RA07327B-s1625

RA-009-C9RA07327B-s1626

RA-009-C9RA07327B-s1627

RA-009-C9RA07327B-s1628

RA-009-C9RA07327B-s1629

RA-009-C9RA07327B-s1630

RA-009-C9RA07327B-s1631

RA-009-C9RA07327B-s1632

RA-009-C9RA07327B-s1633

RA-009-C9RA07327B-s1634

RA-009-C9RA07327B-s1635

RA-009-C9RA07327B-s1636

RA-009-C9RA07327B-s1637

RA-009-C9RA07327B-s1638

RA-009-C9RA07327B-s1639

RA-009-C9RA07327B-s1640

RA-009-C9RA07327B-s1641

RA-009-C9RA07327B-s1642

RA-009-C9RA07327B-s1643

RA-009-C9RA07327B-s1644

RA-009-C9RA07327B-s1645

RA-009-C9RA07327B-s1646

RA-009-C9RA07327B-s1647

RA-009-C9RA07327B-s1648

RA-009-C9RA07327B-s1649

RA-009-C9RA07327B-s1650

RA-009-C9RA07327B-s1651

RA-009-C9RA07327B-s1652

RA-009-C9RA07327B-s1653

RA-009-C9RA07327B-s1654

RA-009-C9RA07327B-s1655

RA-009-C9RA07327B-s1656

RA-009-C9RA07327B-s1657

RA-009-C9RA07327B-s1658

RA-009-C9RA07327B-s1659

RA-009-C9RA07327B-s1660

RA-009-C9RA07327B-s1661

RA-009-C9RA07327B-s1662

RA-009-C9RA07327B-s1663

RA-009-C9RA07327B-s1664

RA-009-C9RA07327B-s1665

RA-009-C9RA07327B-s1666

RA-009-C9RA07327B-s1667

RA-009-C9RA07327B-s1668

RA-009-C9RA07327B-s1669

RA-009-C9RA07327B-s1670

RA-009-C9RA07327B-s1671

RA-009-C9RA07327B-s1672

RA-009-C9RA07327B-s1673

RA-009-C9RA07327B-s1674

RA-009-C9RA07327B-s1675

RA-009-C9RA07327B-s1676

RA-009-C9RA07327B-s1677

RA-009-C9RA07327B-s1678

RA-009-C9RA07327B-s1679

RA-009-C9RA07327B-s1680

RA-009-C9RA07327B-s1681

RA-009-C9RA07327B-s1682

RA-009-C9RA07327B-s1683

RA-009-C9RA07327B-s1684

RA-009-C9RA07327B-s1685

RA-009-C9RA07327B-s1686

RA-009-C9RA07327B-s1687

RA-009-C9RA07327B-s1688

RA-009-C9RA07327B-s1689

RA-009-C9RA07327B-s1690

RA-009-C9RA07327B-s1691

RA-009-C9RA07327B-s1692

RA-009-C9RA07327B-s1693

RA-009-C9RA07327B-s1694

RA-009-C9RA07327B-s1695

RA-009-C9RA07327B-s1696

RA-009-C9RA07327B-s1697

RA-009-C9RA07327B-s1698

RA-009-C9RA07327B-s1699

RA-009-C9RA07327B-s1700

RA-009-C9RA07327B-s1701

RA-009-C9RA07327B-s1702

RA-009-C9RA07327B-s1703

RA-009-C9RA07327B-s1704

RA-009-C9RA07327B-s1705

RA-009-C9RA07327B-s1706

RA-009-C9RA07327B-s1707

RA-009-C9RA07327B-s1708

RA-009-C9RA07327B-s1709

RA-009-C9RA07327B-s1710

RA-009-C9RA07327B-s1711

RA-009-C9RA07327B-s1712

RA-009-C9RA07327B-s1713

RA-009-C9RA07327B-s1714

RA-009-C9RA07327B-s1715

RA-009-C9RA07327B-s1716

RA-009-C9RA07327B-s1717

RA-009-C9RA07327B-s1718

RA-009-C9RA07327B-s1719

RA-009-C9RA07327B-s1720

RA-009-C9RA07327B-s1721

RA-009-C9RA07327B-s1722

RA-009-C9RA07327B-s1723

RA-009-C9RA07327B-s1724

RA-009-C9RA07327B-s1725

RA-009-C9RA07327B-s1726

RA-009-C9RA07327B-s1727

RA-009-C9RA07327B-s1728

RA-009-C9RA07327B-s1729

RA-009-C9RA07327B-s1730

RA-009-C9RA07327B-s1731

RA-009-C9RA07327B-s1732

RA-009-C9RA07327B-s1733

RA-009-C9RA07327B-s1734

RA-009-C9RA07327B-s1735

RA-009-C9RA07327B-s1736

RA-009-C9RA07327B-s1737

RA-009-C9RA07327B-s1738

RA-009-C9RA07327B-s1739

RA-009-C9RA07327B-s1740

RA-009-C9RA07327B-s1741

RA-009-C9RA07327B-s1742

RA-009-C9RA07327B-s1743

RA-009-C9RA07327B-s1744

RA-009-C9RA07327B-s1745

RA-009-C9RA07327B-s1746

RA-009-C9RA07327B-s1747

RA-009-C9RA07327B-s1748

RA-009-C9RA07327B-s1749

RA-009-C9RA07327B-s1750

RA-009-C9RA07327B-s1751

RA-009-C9RA07327B-s1752

RA-009-C9RA07327B-s1753

RA-009-C9RA07327B-s1754

RA-009-C9RA07327B-s1755

RA-009-C9RA07327B-s1756

RA-009-C9RA07327B-s1757

RA-009-C9RA07327B-s1758

RA-009-C9RA07327B-s1759

RA-009-C9RA07327B-s1760

RA-009-C9RA07327B-s1761

RA-009-C9RA07327B-s1762

RA-009-C9RA07327B-s1763

RA-009-C9RA07327B-s1764

RA-009-C9RA07327B-s1765

RA-009-C9RA07327B-s1766

RA-009-C9RA07327B-s1767

RA-009-C9RA07327B-s1768

RA-009-C9RA07327B-s1769

RA-009-C9RA07327B-s1770

RA-009-C9RA07327B-s1771

RA-009-C9RA07327B-s1772

RA-009-C9RA07327B-s1773

RA-009-C9RA07327B-s1774

RA-009-C9RA07327B-s1775

RA-009-C9RA07327B-s1776

RA-009-C9RA07327B-s1777

RA-009-C9RA07327B-s1778

RA-009-C9RA07327B-s1779

RA-009-C9RA07327B-s1780

RA-009-C9RA07327B-s1781

RA-009-C9RA07327B-s1782

RA-009-C9RA07327B-s1783

RA-009-C9RA07327B-s1784

RA-009-C9RA07327B-s1785

RA-009-C9RA07327B-s1786

RA-009-C9RA07327B-s1787

RA-009-C9RA07327B-s1788

RA-009-C9RA07327B-s1789

RA-009-C9RA07327B-s1790

RA-009-C9RA07327B-s1791

RA-009-C9RA07327B-s1792

RA-009-C9RA07327B-s1793

RA-009-C9RA07327B-s1794

RA-009-C9RA07327B-s1795

RA-009-C9RA07327B-s1796

RA-009-C9RA07327B-s1797

RA-009-C9RA07327B-s1798

RA-009-C9RA07327B-s1799

RA-009-C9RA07327B-s1800

RA-009-C9RA07327B-s1801

RA-009-C9RA07327B-s1802

RA-009-C9RA07327B-s1803

RA-009-C9RA07327B-s1804

RA-009-C9RA07327B-s1805

RA-009-C9RA07327B-s1806

RA-009-C9RA07327B-s1807

RA-009-C9RA07327B-s1808

RA-009-C9RA07327B-s1809

RA-009-C9RA07327B-s1810

RA-009-C9RA07327B-s1811

RA-009-C9RA07327B-s1812

RA-009-C9RA07327B-s1813

RA-009-C9RA07327B-s1814

RA-009-C9RA07327B-s1815

RA-009-C9RA07327B-s1816

RA-009-C9RA07327B-s1817

RA-009-C9RA07327B-s1818

RA-009-C9RA07327B-s1819

RA-009-C9RA07327B-s1820

RA-009-C9RA07327B-s1821

RA-009-C9RA07327B-s1822

RA-009-C9RA07327B-s1823

RA-009-C9RA07327B-s1824

RA-009-C9RA07327B-s1825

RA-009-C9RA07327B-s1826

RA-009-C9RA07327B-s1827

RA-009-C9RA07327B-s1828

RA-009-C9RA07327B-s1829

RA-009-C9RA07327B-s1830

RA-009-C9RA07327B-s1831

RA-009-C9RA07327B-s1832

RA-009-C9RA07327B-s1833

RA-009-C9RA07327B-s1834

RA-009-C9RA07327B-s1835

RA-009-C9RA07327B-s1836

RA-009-C9RA07327B-s1837

RA-009-C9RA07327B-s1838

RA-009-C9RA07327B-s1839

RA-009-C9RA07327B-s1840

RA-009-C9RA07327B-s1841

RA-009-C9RA07327B-s1842

RA-009-C9RA07327B-s1843

RA-009-C9RA07327B-s1844

RA-009-C9RA07327B-s1845

RA-009-C9RA07327B-s1846

RA-009-C9RA07327B-s1847

RA-009-C9RA07327B-s1848

RA-009-C9RA07327B-s1849

RA-009-C9RA07327B-s1850

RA-009-C9RA07327B-s1851

RA-009-C9RA07327B-s1852

RA-009-C9RA07327B-s1853

RA-009-C9RA07327B-s1854

RA-009-C9RA07327B-s1855

RA-009-C9RA07327B-s1856

RA-009-C9RA07327B-s1857

RA-009-C9RA07327B-s1858

RA-009-C9RA07327B-s1859

RA-009-C9RA07327B-s1860

RA-009-C9RA07327B-s1861

RA-009-C9RA07327B-s1862

RA-009-C9RA07327B-s1863

RA-009-C9RA07327B-s1864

RA-009-C9RA07327B-s1865

RA-009-C9RA07327B-s1866

RA-009-C9RA07327B-s1867

RA-009-C9RA07327B-s1868

RA-009-C9RA07327B-s1869

RA-009-C9RA07327B-s1870

RA-009-C9RA07327B-s1871

RA-009-C9RA07327B-s1872

RA-009-C9RA07327B-s1873

RA-009-C9RA07327B-s1874

RA-009-C9RA07327B-s1875

RA-009-C9RA07327B-s1876

RA-009-C9RA07327B-s1877

RA-009-C9RA07327B-s1878

RA-009-C9RA07327B-s1879

RA-009-C9RA07327B-s1880

RA-009-C9RA07327B-s1881

RA-009-C9RA07327B-s1882

RA-009-C9RA07327B-s1883

RA-009-C9RA07327B-s1884

RA-009-C9RA07327B-s1885

RA-009-C9RA07327B-s1886

RA-009-C9RA07327B-s1887

RA-009-C9RA07327B-s1888

RA-009-C9RA07327B-s1889

RA-009-C9RA07327B-s1890

RA-009-C9RA07327B-s1891

RA-009-C9RA07327B-s1892

RA-009-C9RA07327B-s1893

RA-009-C9RA07327B-s1894

RA-009-C9RA07327B-s1895

RA-009-C9RA07327B-s1896

RA-009-C9RA07327B-s1897

RA-009-C9RA07327B-s1898

RA-009-C9RA07327B-s1899

RA-009-C9RA07327B-s1900

RA-009-C9RA07327B-s1901

RA-009-C9RA07327B-s1902

RA-009-C9RA07327B-s1903

RA-009-C9RA07327B-s1904

RA-009-C9RA07327B-s1905

RA-009-C9RA07327B-s1906

RA-009-C9RA07327B-s1907

RA-009-C9RA07327B-s1908

RA-009-C9RA07327B-s1909

RA-009-C9RA07327B-s1910

RA-009-C9RA07327B-s1911

RA-009-C9RA07327B-s1912

RA-009-C9RA07327B-s1913

RA-009-C9RA07327B-s1914

RA-009-C9RA07327B-s1915

RA-009-C9RA07327B-s1916

RA-009-C9RA07327B-s1917

RA-009-C9RA07327B-s1918

RA-009-C9RA07327B-s1919

RA-009-C9RA07327B-s1920

RA-009-C9RA07327B-s1921

RA-009-C9RA07327B-s1922

RA-009-C9RA07327B-s1923

RA-009-C9RA07327B-s1924

RA-009-C9RA07327B-s1925

RA-009-C9RA07327B-s1926

RA-009-C9RA07327B-s1927

RA-009-C9RA07327B-s1928

RA-009-C9RA07327B-s1929

RA-009-C9RA07327B-s1930

RA-009-C9RA07327B-s1931

RA-009-C9RA07327B-s1932

RA-009-C9RA07327B-s1933

RA-009-C9RA07327B-s1934

RA-009-C9RA07327B-s1935

RA-009-C9RA07327B-s1936

RA-009-C9RA07327B-s1937

RA-009-C9RA07327B-s1938

RA-009-C9RA07327B-s1939

RA-009-C9RA07327B-s1940

RA-009-C9RA07327B-s1941

RA-009-C9RA07327B-s1942

RA-009-C9RA07327B-s1943

RA-009-C9RA07327B-s1944

RA-009-C9RA07327B-s1945

RA-009-C9RA07327B-s1946

RA-009-C9RA07327B-s1947

RA-009-C9RA07327B-s1948

RA-009-C9RA07327B-s1949

RA-009-C9RA07327B-s1950

RA-009-C9RA07327B-s1951

RA-009-C9RA07327B-s1952

RA-009-C9RA07327B-s1953

RA-009-C9RA07327B-s1954

RA-009-C9RA07327B-s1955

RA-009-C9RA07327B-s1956

RA-009-C9RA07327B-s1957

RA-009-C9RA07327B-s1958

RA-009-C9RA07327B-s1959

RA-009-C9RA07327B-s1960

RA-009-C9RA07327B-s1961

RA-009-C9RA07327B-s1962

RA-009-C9RA07327B-s1963

RA-009-C9RA07327B-s1964

RA-009-C9RA07327B-s1965

RA-009-C9RA07327B-s1966

RA-009-C9RA07327B-s1967

RA-009-C9RA07327B-s1968

RA-009-C9RA07327B-s1969

RA-009-C9RA07327B-s1970

RA-009-C9RA07327B-s1971

RA-009-C9RA07327B-s1972

RA-009-C9RA07327B-s1973

RA-009-C9RA07327B-s1974

RA-009-C9RA07327B-s1975

RA-009-C9RA07327B-s1976

RA-009-C9RA07327B-s1977

RA-009-C9RA07327B-s1978

RA-009-C9RA07327B-s1979

RA-009-C9RA07327B-s1980

RA-009-C9RA07327B-s1981

RA-009-C9RA07327B-s1982

RA-009-C9RA07327B-s1983

RA-009-C9RA07327B-s1984

RA-009-C9RA07327B-s1985

RA-009-C9RA07327B-s1986

RA-009-C9RA07327B-s1987

RA-009-C9RA07327B-s1988

RA-009-C9RA07327B-s1989

RA-009-C9RA07327B-s1990

RA-009-C9RA07327B-s1991

RA-009-C9RA07327B-s1992

RA-009-C9RA07327B-s1993

RA-009-C9RA07327B-s1994

RA-009-C9RA07327B-s1995

RA-009-C9RA07327B-s1996

RA-009-C9RA07327B-s1997

RA-009-C9RA07327B-s1998

RA-009-C9RA07327B-s1999

RA-009-C9RA07327B-s2000

RA-009-C9RA07327B-s2001

RA-009-C9RA07327B-s2002

RA-009-C9RA07327B-s2003

RA-009-C9RA07327B-s2004

RA-009-C9RA07327B-s2005

RA-009-C9RA07327B-s2006

RA-009-C9RA07327B-s2007

RA-009-C9RA07327B-s2008

RA-009-C9RA07327B-s2009

RA-009-C9RA07327B-s2010

RA-009-C9RA07327B-s2011

RA-009-C9RA07327B-s2012

RA-009-C9RA07327B-s2013

RA-009-C9RA07327B-s2014

RA-009-C9RA07327B-s2015

RA-009-C9RA07327B-s2016

RA-009-C9RA07327B-s2017

RA-009-C9RA07327B-s2018

RA-009-C9RA07327B-s2019

RA-009-C9RA07327B-s2020

RA-009-C9RA07327B-s2021

RA-009-C9RA07327B-s2022

RA-009-C9RA07327B-s2023

RA-009-C9RA07327B-s2024

RA-009-C9RA07327B-s2025

RA-009-C9RA07327B-s2026

RA-009-C9RA07327B-s2027

RA-009-C9RA07327B-s2028

RA-009-C9RA07327B-s2029

RA-009-C9RA07327B-s2030

RA-009-C9RA07327B-s2031

RA-009-C9RA07327B-s2032

RA-009-C9RA07327B-s2033

RA-009-C9RA07327B-s2034

RA-009-C9RA07327B-s2035

RA-009-C9RA07327B-s2036

RA-009-C9RA07327B-s2037

RA-009-C9RA07327B-s2038

RA-009-C9RA07327B-s2039

RA-009-C9RA07327B-s2040

RA-009-C9RA07327B-s2041

RA-009-C9RA07327B-s2042

RA-009-C9RA07327B-s2043

RA-009-C9RA07327B-s2044

RA-009-C9RA07327B-s2045

RA-009-C9RA07327B-s2046

RA-009-C9RA07327B-s2047

RA-009-C9RA07327B-s2048

RA-009-C9RA07327B-s2049

RA-009-C9RA07327B-s2050

RA-009-C9RA07327B-s2051

RA-009-C9RA07327B-s2052

RA-009-C9RA07327B-s2053

RA-009-C9RA07327B-s2054

RA-009-C9RA07327B-s2055

RA-009-C9RA07327B-s2056

RA-009-C9RA07327B-s2057

RA-009-C9RA07327B-s2058

RA-009-C9RA07327B-s2059

RA-009-C9RA07327B-s2060

RA-009-C9RA07327B-s2061

RA-009-C9RA07327B-s2062

RA-009-C9RA07327B-s2063

RA-009-C9RA07327B-s2064

RA-009-C9RA07327B-s2065

RA-009-C9RA07327B-s2066

RA-009-C9RA07327B-s2067

RA-009-C9RA07327B-s2068

RA-009-C9RA07327B-s2069

RA-009-C9RA07327B-s2070

RA-009-C9RA07327B-s2071

RA-009-C9RA07327B-s2072

RA-009-C9RA07327B-s2073

RA-009-C9RA07327B-s2074

RA-009-C9RA07327B-s2075

RA-009-C9RA07327B-s2076

RA-009-C9RA07327B-s2077

RA-009-C9RA07327B-s2078

RA-009-C9RA07327B-s2079

RA-009-C9RA07327B-s2080

RA-009-C9RA07327B-s2081

RA-009-C9RA07327B-s2082

RA-009-C9RA07327B-s2083

RA-009-C9RA07327B-s2084

RA-009-C9RA07327B-s2085

RA-009-C9RA07327B-s2086

RA-009-C9RA07327B-s2087

RA-009-C9RA07327B-s2088

RA-009-C9RA07327B-s2089

RA-009-C9RA07327B-s2090

RA-009-C9RA07327B-s2091

RA-009-C9RA07327B-s2092

RA-009-C9RA07327B-s2093

RA-009-C9RA07327B-s2094

RA-009-C9RA07327B-s2095

RA-009-C9RA07327B-s2096

RA-009-C9RA07327B-s2097

RA-009-C9RA07327B-s2098

RA-009-C9RA07327B-s2099

RA-009-C9RA07327B-s2100

RA-009-C9RA07327B-s2101

RA-009-C9RA07327B-s2102

RA-009-C9RA07327B-s2103

RA-009-C9RA07327B-s2104

RA-009-C9RA07327B-s2105

RA-009-C9RA07327B-s2106

RA-009-C9RA07327B-s2107

RA-009-C9RA07327B-s2108

RA-009-C9RA07327B-s2109

RA-009-C9RA07327B-s2110

RA-009-C9RA07327B-s2111

RA-009-C9RA07327B-s2112

RA-009-C9RA07327B-s2113

RA-009-C9RA07327B-s2114

RA-009-C9RA07327B-s2115

RA-009-C9RA07327B-s2116

RA-009-C9RA07327B-s2117

RA-009-C9RA07327B-s2118

RA-009-C9RA07327B-s2119

RA-009-C9RA07327B-s2120

RA-009-C9RA07327B-s2121

RA-009-C9RA07327B-s2122

RA-009-C9RA07327B-s2123

RA-009-C9RA07327B-s2124

RA-009-C9RA07327B-s2125

RA-009-C9RA07327B-s2126

RA-009-C9RA07327B-s2127

RA-009-C9RA07327B-s2128

RA-009-C9RA07327B-s2129

RA-009-C9RA07327B-s2130

RA-009-C9RA07327B-s2131

RA-009-C9RA07327B-s2132

RA-009-C9RA07327B-s2133

RA-009-C9RA07327B-s2134

RA-009-C9RA07327B-s2135

RA-009-C9RA07327B-s2136

RA-009-C9RA07327B-s2137

RA-009-C9RA07327B-s2138

RA-009-C9RA07327B-s2139

RA-009-C9RA07327B-s2140

RA-009-C9RA07327B-s2141

RA-009-C9RA07327B-s2142

RA-009-C9RA07327B-s2143

RA-009-C9RA07327B-s2144

RA-009-C9RA07327B-s2145

RA-009-C9RA07327B-s2146

RA-009-C9RA07327B-s2147

RA-009-C9RA07327B-s2148

RA-009-C9RA07327B-s2149

RA-009-C9RA07327B-s2150

RA-009-C9RA07327B-s2151

RA-009-C9RA07327B-s2152

RA-009-C9RA07327B-s2153

RA-009-C9RA07327B-s2154

RA-009-C9RA07327B-s2155

RA-009-C9RA07327B-s2156

RA-009-C9RA07327B-s2157

RA-009-C9RA07327B-s2158

RA-009-C9RA07327B-s2159

RA-009-C9RA07327B-s2160

RA-009-C9RA07327B-s2161

RA-009-C9RA07327B-s2162

RA-009-C9RA07327B-s2163

RA-009-C9RA07327B-s2164

RA-009-C9RA07327B-s2165

RA-009-C9RA07327B-s2166

RA-009-C9RA07327B-s2167

RA-009-C9RA07327B-s2168

RA-009-C9RA07327B-s2169

RA-009-C9RA07327B-s2170

RA-009-C9RA07327B-s2171

RA-009-C9RA07327B-s2172

RA-009-C9RA07327B-s2173

RA-009-C9RA07327B-s2174

RA-009-C9RA07327B-s2175

RA-009-C9RA07327B-s2176

RA-009-C9RA07327B-s2177

RA-009-C9RA07327B-s2178

RA-009-C9RA07327B-s2179

RA-009-C9RA07327B-s2180

RA-009-C9RA07327B-s2181

RA-009-C9RA07327B-s2182

RA-009-C9RA07327B-s2183

RA-009-C9RA07327B-s2184

RA-009-C9RA07327B-s2185

RA-009-C9RA07327B-s2186

RA-009-C9RA07327B-s2187

RA-009-C9RA07327B-s2188

RA-009-C9RA07327B-s2189

RA-009-C9RA07327B-s2190

RA-009-C9RA07327B-s2191

RA-009-C9RA07327B-s2192

RA-009-C9RA07327B-s2193

RA-009-C9RA07327B-s2194

RA-009-C9RA07327B-s2195

RA-009-C9RA07327B-s2196

RA-009-C9RA07327B-s2197

RA-009-C9RA07327B-s2198

RA-009-C9RA07327B-s2199

RA-009-C9RA07327B-s2200

RA-009-C9RA07327B-s2201

RA-009-C9RA07327B-s2202

RA-009-C9RA07327B-s2203

RA-009-C9RA07327B-s2204

RA-009-C9RA07327B-s2205

RA-009-C9RA07327B-s2206

RA-009-C9RA07327B-s2207

RA-009-C9RA07327B-s2208

RA-009-C9RA07327B-s2209

RA-009-C9RA07327B-s2210

RA-009-C9RA07327B-s2211

RA-009-C9RA07327B-s2212

RA-009-C9RA07327B-s2213

RA-009-C9RA07327B-s2214

RA-009-C9RA07327B-s2215

RA-009-C9RA07327B-s2216

RA-009-C9RA07327B-s2217

RA-009-C9RA07327B-s2218

RA-009-C9RA07327B-s2219

RA-009-C9RA07327B-s2220

RA-009-C9RA07327B-s2221

RA-009-C9RA07327B-s2222

RA-009-C9RA07327B-s2223

RA-009-C9RA07327B-s2224

RA-009-C9RA07327B-s2225

RA-009-C9RA07327B-s2226

RA-009-C9RA07327B-s2227

RA-009-C9RA07327B-s2228

RA-009-C9RA07327B-s2229

RA-009-C9RA07327B-s2230

RA-009-C9RA07327B-s2231

RA-009-C9RA07327B-s2232

RA-009-C9RA07327B-s2233

RA-009-C9RA07327B-s2234

RA-009-C9RA07327B-s2235

RA-009-C9RA07327B-s2236

RA-009-C9RA07327B-s2237

RA-009-C9RA07327B-s2238

RA-009-C9RA07327B-s2239

RA-009-C9RA07327B-s2240

RA-009-C9RA07327B-s2241

RA-009-C9RA07327B-s2242

RA-009-C9RA07327B-s2243

RA-009-C9RA07327B-s2244

RA-009-C9RA07327B-s2245

RA-009-C9RA07327B-s2246

RA-009-C9RA07327B-s2247

RA-009-C9RA07327B-s2248

RA-009-C9RA07327B-s2249

RA-009-C9RA07327B-s2250

RA-009-C9RA07327B-s2251

RA-009-C9RA07327B-s2252

RA-009-C9RA07327B-s2253

RA-009-C9RA07327B-s2254

RA-009-C9RA07327B-s2255

RA-009-C9RA07327B-s2256

RA-009-C9RA07327B-s2257

RA-009-C9RA07327B-s2258

RA-009-C9RA07327B-s2259

RA-009-C9RA07327B-s2260

RA-009-C9RA07327B-s2261

RA-009-C9RA07327B-s2262

RA-009-C9RA07327B-s2263

RA-009-C9RA07327B-s2264

RA-009-C9RA07327B-s2265

RA-009-C9RA07327B-s2266

RA-009-C9RA07327B-s2267

RA-009-C9RA07327B-s2268

RA-009-C9RA07327B-s2269

RA-009-C9RA07327B-s2270

RA-009-C9RA07327B-s2271

RA-009-C9RA07327B-s2272

RA-009-C9RA07327B-s2273

RA-009-C9RA07327B-s2274

RA-009-C9RA07327B-s2275

RA-009-C9RA07327B-s2276

RA-009-C9RA07327B-s2277

RA-009-C9RA07327B-s2278

RA-009-C9RA07327B-s2279

RA-009-C9RA07327B-s2280

RA-009-C9RA07327B-s2281

RA-009-C9RA07327B-s2282

RA-009-C9RA07327B-s2283

RA-009-C9RA07327B-s2284

RA-009-C9RA07327B-s2285

RA-009-C9RA07327B-s2286

RA-009-C9RA07327B-s2287

RA-009-C9RA07327B-s2288

RA-009-C9RA07327B-s2289

RA-009-C9RA07327B-s2290

RA-009-C9RA07327B-s2291

RA-009-C9RA07327B-s2292

RA-009-C9RA07327B-s2293

RA-009-C9RA07327B-s2294

RA-009-C9RA07327B-s2295

RA-009-C9RA07327B-s2296

RA-009-C9RA07327B-s2297

RA-009-C9RA07327B-s2298

RA-009-C9RA07327B-s2299

RA-009-C9RA07327B-s2300

RA-009-C9RA07327B-s2301

RA-009-C9RA07327B-s2302

RA-009-C9RA07327B-s2303

RA-009-C9RA07327B-s2304

RA-009-C9RA07327B-s2305

RA-009-C9RA07327B-s2306

RA-009-C9RA07327B-s2307

RA-009-C9RA07327B-s2308

RA-009-C9RA07327B-s2309

RA-009-C9RA07327B-s2310

RA-009-C9RA07327B-s2311

RA-009-C9RA07327B-s2312

RA-009-C9RA07327B-s2313

RA-009-C9RA07327B-s2314

RA-009-C9RA07327B-s2315

RA-009-C9RA07327B-s2316

RA-009-C9RA07327B-s2317

RA-009-C9RA07327B-s2318

RA-009-C9RA07327B-s2319

RA-009-C9RA07327B-s2320

RA-009-C9RA07327B-s2321

RA-009-C9RA07327B-s2322

RA-009-C9RA07327B-s2323

RA-009-C9RA07327B-s2324

RA-009-C9RA07327B-s2325

RA-009-C9RA07327B-s2326

RA-009-C9RA07327B-s2327

RA-009-C9RA07327B-s2328

RA-009-C9RA07327B-s2329

RA-009-C9RA07327B-s2330

RA-009-C9RA07327B-s2331

RA-009-C9RA07327B-s2332

RA-009-C9RA07327B-s2333

RA-009-C9RA07327B-s2334

RA-009-C9RA07327B-s2335

RA-009-C9RA07327B-s2336

RA-009-C9RA07327B-s2337

RA-009-C9RA07327B-s2338

RA-009-C9RA07327B-s2339

RA-009-C9RA07327B-s2340

RA-009-C9RA07327B-s2341

RA-009-C9RA07327B-s2342

RA-009-C9RA07327B-s2343

RA-009-C9RA07327B-s2344

RA-009-C9RA07327B-s2345

RA-009-C9RA07327B-s2346

RA-009-C9RA07327B-s2347

RA-009-C9RA07327B-s2348

RA-009-C9RA07327B-s2349

RA-009-C9RA07327B-s2350

RA-009-C9RA07327B-s2351

RA-009-C9RA07327B-s2352

RA-009-C9RA07327B-s2353

RA-009-C9RA07327B-s2354

RA-009-C9RA07327B-s2355

RA-009-C9RA07327B-s2356

RA-009-C9RA07327B-s2357

RA-009-C9RA07327B-s2358

RA-009-C9RA07327B-s2359

RA-009-C9RA07327B-s2360

RA-009-C9RA07327B-s2361

RA-009-C9RA07327B-s2362

RA-009-C9RA07327B-s2363

RA-009-C9RA07327B-s2364

RA-009-C9RA07327B-s2365

RA-009-C9RA07327B-s2366

RA-009-C9RA07327B-s2367

RA-009-C9RA07327B-s2368

RA-009-C9RA07327B-s2369

RA-009-C9RA07327B-s2370

RA-009-C9RA07327B-s2371

RA-009-C9RA07327B-s2372

RA-009-C9RA07327B-s2373

RA-009-C9RA07327B-s2374

RA-009-C9RA07327B-s2375

RA-009-C9RA07327B-s2376

RA-009-C9RA07327B-s2377

RA-009-C9RA07327B-s2378

RA-009-C9RA07327B-s2379

RA-009-C9RA07327B-s2380

RA-009-C9RA07327B-s2381

RA-009-C9RA07327B-s2382

RA-009-C9RA07327B-s2383

RA-009-C9RA07327B-s2384

RA-009-C9RA07327B-s2385

RA-009-C9RA07327B-s2386

RA-009-C9RA07327B-s2387

RA-009-C9RA07327B-s2388

RA-009-C9RA07327B-s2389

RA-009-C9RA07327B-s2390

RA-009-C9RA07327B-s2391

RA-009-C9RA07327B-s2392

RA-009-C9RA07327B-s2393

RA-009-C9RA07327B-s2394

RA-009-C9RA07327B-s2395

RA-009-C9RA07327B-s2396

RA-009-C9RA07327B-s2397

RA-009-C9RA07327B-s2398

RA-009-C9RA07327B-s2399

RA-009-C9RA07327B-s2400

RA-009-C9RA07327B-s2401

RA-009-C9RA07327B-s2402

RA-009-C9RA07327B-s2403

RA-009-C9RA07327B-s2404

RA-009-C9RA07327B-s2405

RA-009-C9RA07327B-s2406

RA-009-C9RA07327B-s2407

RA-009-C9RA07327B-s2408

RA-009-C9RA07327B-s2409

RA-009-C9RA07327B-s2410

RA-009-C9RA07327B-s2411

RA-009-C9RA07327B-s2412

RA-009-C9RA07327B-s2413

RA-009-C9RA07327B-s2414

RA-009-C9RA07327B-s2415

RA-009-C9RA07327B-s2416

RA-009-C9RA07327B-s2417

RA-009-C9RA07327B-s2418

RA-009-C9RA07327B-s2419

RA-009-C9RA07327B-s2420

RA-009-C9RA07327B-s2421

RA-009-C9RA07327B-s2422

RA-009-C9RA07327B-s2423

RA-009-C9RA07327B-s2424

RA-009-C9RA07327B-s2425

RA-009-C9RA07327B-s2426

RA-009-C9RA07327B-s2427

RA-009-C9RA07327B-s2428

RA-009-C9RA07327B-s2429

RA-009-C9RA07327B-s2430

RA-009-C9RA07327B-s2431

RA-009-C9RA07327B-s2432

RA-009-C9RA07327B-s2433

RA-009-C9RA07327B-s2434

RA-009-C9RA07327B-s2435

RA-009-C9RA07327B-s2436

RA-009-C9RA07327B-s2437

RA-009-C9RA07327B-s2438

RA-009-C9RA07327B-s2439

RA-009-C9RA07327B-s2440

RA-009-C9RA07327B-s2441

RA-009-C9RA07327B-s2442

RA-009-C9RA07327B-s2443

RA-009-C9RA07327B-s2444

RA-009-C9RA07327B-s2445

RA-009-C9RA07327B-s2446

RA-009-C9RA07327B-s2447

RA-009-C9RA07327B-s2448

RA-009-C9RA07327B-s2449

RA-009-C9RA07327B-s2450

RA-009-C9RA07327B-s2451

RA-009-C9RA07327B-s2452

RA-009-C9RA07327B-s2453

RA-009-C9RA07327B-s2454

RA-009-C9RA07327B-s2455

RA-009-C9RA07327B-s2456

RA-009-C9RA07327B-s2457

RA-009-C9RA07327B-s2458

RA-009-C9RA07327B-s2459

RA-009-C9RA07327B-s2460

RA-009-C9RA07327B-s2461

RA-009-C9RA07327B-s2462

RA-009-C9RA07327B-s2463

RA-009-C9RA07327B-s2464

RA-009-C9RA07327B-s2465

RA-009-C9RA07327B-s2466

RA-009-C9RA07327B-s2467

RA-009-C9RA07327B-s2468

RA-009-C9RA07327B-s2469

RA-009-C9RA07327B-s2470

RA-009-C9RA07327B-s2471

RA-009-C9RA07327B-s2472

RA-009-C9RA07327B-s2473

RA-009-C9RA07327B-s2474

RA-009-C9RA07327B-s2475

RA-009-C9RA07327B-s2476

RA-009-C9RA07327B-s2477

RA-009-C9RA07327B-s2478

RA-009-C9RA07327B-s2479

RA-009-C9RA07327B-s2480

RA-009-C9RA07327B-s2481

RA-009-C9RA07327B-s2482

RA-009-C9RA07327B-s2483

RA-009-C9RA07327B-s2484

RA-009-C9RA07327B-s2485

RA-009-C9RA07327B-s2486

RA-009-C9RA07327B-s2487

RA-009-C9RA07327B-s2488

RA-009-C9RA07327B-s2489

RA-009-C9RA07327B-s2490

RA-009-C9RA07327B-s2491

RA-009-C9RA07327B-s2492

RA-009-C9RA07327B-s2493

RA-009-C9RA07327B-s2494

RA-009-C9RA07327B-s2495

RA-009-C9RA07327B-s2496

RA-009-C9RA07327B-s2497

RA-009-C9RA07327B-s2498

RA-009-C9RA07327B-s2499

RA-009-C9RA07327B-s2500

RA-009-C9RA07327B-s2501

RA-009-C9RA07327B-s2502

RA-009-C9RA07327B-s2503

RA-009-C9RA07327B-s2504

RA-009-C9RA07327B-s2505

RA-009-C9RA07327B-s2506

RA-009-C9RA07327B-s2507

RA-009-C9RA07327B-s2508

RA-009-C9RA07327B-s2509

RA-009-C9RA07327B-s2510

RA-009-C9RA07327B-s2511

RA-009-C9RA07327B-s2512

RA-009-C9RA07327B-s2513

RA-009-C9RA07327B-s2514

RA-009-C9RA07327B-s2515

RA-009-C9RA07327B-s2516

RA-009-C9RA07327B-s2517

RA-009-C9RA07327B-s2518

RA-009-C9RA07327B-s2519

RA-009-C9RA07327B-s2520

RA-009-C9RA07327B-s2521

RA-009-C9RA07327B-s2522

RA-009-C9RA07327B-s2523

RA-009-C9RA07327B-s2524

RA-009-C9RA07327B-s2525

RA-009-C9RA07327B-s2526

RA-009-C9RA07327B-s2527

RA-009-C9RA07327B-s2528

RA-009-C9RA07327B-s2529

RA-009-C9RA07327B-s2530

RA-009-C9RA07327B-s2531

RA-009-C9RA07327B-s2532

RA-009-C9RA07327B-s2533

RA-009-C9RA07327B-s2534

RA-009-C9RA07327B-s2535

RA-009-C9RA07327B-s2536

RA-009-C9RA07327B-s2537

RA-009-C9RA07327B-s2538

RA-009-C9RA07327B-s2539

RA-009-C9RA07327B-s2540

RA-009-C9RA07327B-s2541

RA-009-C9RA07327B-s2542

RA-009-C9RA07327B-s2543

RA-009-C9RA07327B-s2544

RA-009-C9RA07327B-s2545

RA-009-C9RA07327B-s2546

RA-009-C9RA07327B-s2547

RA-009-C9RA07327B-s2548

RA-009-C9RA07327B-s2549

RA-009-C9RA07327B-s2550

RA-009-C9RA07327B-s2551

RA-009-C9RA07327B-s2552

RA-009-C9RA07327B-s2553

RA-009-C9RA07327B-s2554

RA-009-C9RA07327B-s2555

RA-009-C9RA07327B-s2556

RA-009-C9RA07327B-s2557

RA-009-C9RA07327B-s2558

RA-009-C9RA07327B-s2559

RA-009-C9RA07327B-s2560

RA-009-C9RA07327B-s2561

RA-009-C9RA07327B-s2562

RA-009-C9RA07327B-s2563

RA-009-C9RA07327B-s2564

RA-009-C9RA07327B-s2565

RA-009-C9RA07327B-s2566

RA-009-C9RA07327B-s2567

RA-009-C9RA07327B-s2568

RA-009-C9RA07327B-s2569

RA-009-C9RA07327B-s2570

RA-009-C9RA07327B-s2571

RA-009-C9RA07327B-s2572

RA-009-C9RA07327B-s2573

RA-009-C9RA07327B-s2574

RA-009-C9RA07327B-s2575

RA-009-C9RA07327B-s2576

RA-009-C9RA07327B-s2577

RA-009-C9RA07327B-s2578

RA-009-C9RA07327B-s2579

RA-009-C9RA07327B-s2580

RA-009-C9RA07327B-s2581

RA-009-C9RA07327B-s2582

RA-009-C9RA07327B-s2583

RA-009-C9RA07327B-s2584

RA-009-C9RA07327B-s2585

RA-009-C9RA07327B-s2586

RA-009-C9RA07327B-s2587

RA-009-C9RA07327B-s2588

RA-009-C9RA07327B-s2589

RA-009-C9RA07327B-s2590

RA-009-C9RA07327B-s2591

RA-009-C9RA07327B-s2592

RA-009-C9RA07327B-s2593

RA-009-C9RA07327B-s2594

RA-009-C9RA07327B-s2595

RA-009-C9RA07327B-s2596

RA-009-C9RA07327B-s2597

RA-009-C9RA07327B-s2598

RA-009-C9RA07327B-s2599

RA-009-C9RA07327B-s2600

RA-009-C9RA07327B-s2601

RA-009-C9RA07327B-s2602

RA-009-C9RA07327B-s2603

RA-009-C9RA07327B-s2604

RA-009-C9RA07327B-s2605

RA-009-C9RA07327B-s2606

RA-009-C9RA07327B-s2607

RA-009-C9RA07327B-s2608

RA-009-C9RA07327B-s2609

RA-009-C9RA07327B-s2610

RA-009-C9RA07327B-s2611

RA-009-C9RA07327B-s2612

RA-009-C9RA07327B-s2613

RA-009-C9RA07327B-s2614

RA-009-C9RA07327B-s2615

RA-009-C9RA07327B-s2616

RA-009-C9RA07327B-s2617

RA-009-C9RA07327B-s2618

RA-009-C9RA07327B-s2619

RA-009-C9RA07327B-s2620

RA-009-C9RA07327B-s2621

RA-009-C9RA07327B-s2622

RA-009-C9RA07327B-s2623

RA-009-C9RA07327B-s2624

RA-009-C9RA07327B-s2625

RA-009-C9RA07327B-s2626

RA-009-C9RA07327B-s2627

RA-009-C9RA07327B-s2628

RA-009-C9RA07327B-s2629

RA-009-C9RA07327B-s2630

RA-009-C9RA07327B-s2631

RA-009-C9RA07327B-s2632

RA-009-C9RA07327B-s2633

RA-009-C9RA07327B-s2634

RA-009-C9RA07327B-s2635

RA-009-C9RA07327B-s2636

RA-009-C9RA07327B-s2637

RA-009-C9RA07327B-s2638

RA-009-C9RA07327B-s2639

RA-009-C9RA07327B-s2640

RA-009-C9RA07327B-s2641

RA-009-C9RA07327B-s2642

RA-009-C9RA07327B-s2643

RA-009-C9RA07327B-s2644

RA-009-C9RA07327B-s2645

RA-009-C9RA07327B-s2646

RA-009-C9RA07327B-s2647

RA-009-C9RA07327B-s2648

RA-009-C9RA07327B-s2649

RA-009-C9RA07327B-s2650

RA-009-C9RA07327B-s2651

RA-009-C9RA07327B-s2652

RA-009-C9RA07327B-s2653

RA-009-C9RA07327B-s2654

RA-009-C9RA07327B-s2655

RA-009-C9RA07327B-s2656

RA-009-C9RA07327B-s2657

RA-009-C9RA07327B-s2658

RA-009-C9RA07327B-s2659

RA-009-C9RA07327B-s2660

RA-009-C9RA07327B-s2661

RA-009-C9RA07327B-s2662

RA-009-C9RA07327B-s2663

RA-009-C9RA07327B-s2664

RA-009-C9RA07327B-s2665

RA-009-C9RA07327B-s2666

RA-009-C9RA07327B-s2667

RA-009-C9RA07327B-s2668

RA-009-C9RA07327B-s2669

RA-009-C9RA07327B-s2670

RA-009-C9RA07327B-s2671

RA-009-C9RA07327B-s2672

RA-009-C9RA07327B-s2673

RA-009-C9RA07327B-s2674

RA-009-C9RA07327B-s2675

RA-009-C9RA07327B-s2676

RA-009-C9RA07327B-s2677

RA-009-C9RA07327B-s2678

RA-009-C9RA07327B-s2679

RA-009-C9RA07327B-s2680

RA-009-C9RA07327B-s2681

RA-009-C9RA07327B-s2682

RA-009-C9RA07327B-s2683

RA-009-C9RA07327B-s2684

RA-009-C9RA07327B-s2685

RA-009-C9RA07327B-s2686

RA-009-C9RA07327B-s2687

RA-009-C9RA07327B-s2688

RA-009-C9RA07327B-s2689

RA-009-C9RA07327B-s2690

RA-009-C9RA07327B-s2691

RA-009-C9RA07327B-s2692

RA-009-C9RA07327B-s2693

RA-009-C9RA07327B-s2694

RA-009-C9RA07327B-s2695

RA-009-C9RA07327B-s2696

RA-009-C9RA07327B-s2697

RA-009-C9RA07327B-s2698

RA-009-C9RA07327B-s2699

RA-009-C9RA07327B-s2700

RA-009-C9RA07327B-s2701

RA-009-C9RA07327B-s2702

RA-009-C9RA07327B-s2703

RA-009-C9RA07327B-s2704

RA-009-C9RA07327B-s2705

RA-009-C9RA07327B-s2706

RA-009-C9RA07327B-s2707

RA-009-C9RA07327B-s2708

RA-009-C9RA07327B-s2709

RA-009-C9RA07327B-s2710

RA-009-C9RA07327B-s2711

RA-009-C9RA07327B-s2712

RA-009-C9RA07327B-s2713

RA-009-C9RA07327B-s2714

RA-009-C9RA07327B-s2715

RA-009-C9RA07327B-s2716

RA-009-C9RA07327B-s2717

RA-009-C9RA07327B-s2718

RA-009-C9RA07327B-s2719

RA-009-C9RA07327B-s2720

RA-009-C9RA07327B-s2721

RA-009-C9RA07327B-s2722

RA-009-C9RA07327B-s2723

RA-009-C9RA07327B-s2724

RA-009-C9RA07327B-s2725

RA-009-C9RA07327B-s2726

RA-009-C9RA07327B-s2727

RA-009-C9RA07327B-s2728

RA-009-C9RA07327B-s2729

RA-009-C9RA07327B-s2730

RA-009-C9RA07327B-s2731

RA-009-C9RA07327B-s2732

RA-009-C9RA07327B-s2733

RA-009-C9RA07327B-s2734

RA-009-C9RA07327B-s2735

RA-009-C9RA07327B-s2736

RA-009-C9RA07327B-s2737

RA-009-C9RA07327B-s2738

RA-009-C9RA07327B-s2739

RA-009-C9RA07327B-s2740

RA-009-C9RA07327B-s2741

RA-009-C9RA07327B-s2742

RA-009-C9RA07327B-s2743

RA-009-C9RA07327B-s2744

RA-009-C9RA07327B-s2745

RA-009-C9RA07327B-s2746

RA-009-C9RA07327B-s2747

RA-009-C9RA07327B-s2748

RA-009-C9RA07327B-s2749

RA-009-C9RA07327B-s2750

RA-009-C9RA07327B-s2751

RA-009-C9RA07327B-s2752

RA-009-C9RA07327B-s2753

RA-009-C9RA07327B-s2754

RA-009-C9RA07327B-s2755

RA-009-C9RA07327B-s2756

RA-009-C9RA07327B-s2757

RA-009-C9RA07327B-s2758

RA-009-C9RA07327B-s2759

RA-009-C9RA07327B-s2760

RA-009-C9RA07327B-s2761

RA-009-C9RA07327B-s2762

RA-009-C9RA07327B-s2763

RA-009-C9RA07327B-s2764

RA-009-C9RA07327B-s2765

RA-009-C9RA07327B-s2766

RA-009-C9RA07327B-s2767

RA-009-C9RA07327B-s2768

RA-009-C9RA07327B-s2769

RA-009-C9RA07327B-s2770

RA-009-C9RA07327B-s2771

RA-009-C9RA07327B-s2772

RA-009-C9RA07327B-s2773

RA-009-C9RA07327B-s2774

RA-009-C9RA07327B-s2775

RA-009-C9RA07327B-s2776

RA-009-C9RA07327B-s2777

RA-009-C9RA07327B-s2778

RA-009-C9RA07327B-s2779

RA-009-C9RA07327B-s2780

RA-009-C9RA07327B-s2781

RA-009-C9RA07327B-s2782

RA-009-C9RA07327B-s2783

RA-009-C9RA07327B-s2784

RA-009-C9RA07327B-s2785

RA-009-C9RA07327B-s2786

RA-009-C9RA07327B-s2787

RA-009-C9RA07327B-s2788

RA-009-C9RA07327B-s2789

RA-009-C9RA07327B-s2790

RA-009-C9RA07327B-s2791

RA-009-C9RA07327B-s2792

RA-009-C9RA07327B-s2793

RA-009-C9RA07327B-s2794

RA-009-C9RA07327B-s2795

RA-009-C9RA07327B-s2796

RA-009-C9RA07327B-s2797

RA-009-C9RA07327B-s2798

RA-009-C9RA07327B-s2799

RA-009-C9RA07327B-s2800

RA-009-C9RA07327B-s2801

RA-009-C9RA07327B-s2802

RA-009-C9RA07327B-s2803

RA-009-C9RA07327B-s2804

RA-009-C9RA07327B-s2805

RA-009-C9RA07327B-s2806

RA-009-C9RA07327B-s2807

RA-009-C9RA07327B-s2808

RA-009-C9RA07327B-s2809

RA-009-C9RA07327B-s2810

RA-009-C9RA07327B-s2811

RA-009-C9RA07327B-s2812

RA-009-C9RA07327B-s2813

RA-009-C9RA07327B-s2814

RA-009-C9RA07327B-s2815

RA-009-C9RA07327B-s2816

RA-009-C9RA07327B-s2817

RA-009-C9RA07327B-s2818

RA-009-C9RA07327B-s2819

RA-009-C9RA07327B-s2820

RA-009-C9RA07327B-s2821

RA-009-C9RA07327B-s2822

RA-009-C9RA07327B-s2823

RA-009-C9RA07327B-s2824

RA-009-C9RA07327B-s2825

RA-009-C9RA07327B-s2826

RA-009-C9RA07327B-s2827

RA-009-C9RA07327B-s2828

RA-009-C9RA07327B-s2829

RA-009-C9RA07327B-s2830

RA-009-C9RA07327B-s2831

RA-009-C9RA07327B-s2832

RA-009-C9RA07327B-s2833

RA-009-C9RA07327B-s2834

RA-009-C9RA07327B-s2835

RA-009-C9RA07327B-s2836

RA-009-C9RA07327B-s2837

RA-009-C9RA07327B-s2838

RA-009-C9RA07327B-s2839

RA-009-C9RA07327B-s2840

RA-009-C9RA07327B-s2841

RA-009-C9RA07327B-s2842

RA-009-C9RA07327B-s2843

RA-009-C9RA07327B-s2844

RA-009-C9RA07327B-s2845

RA-009-C9RA07327B-s2846

RA-009-C9RA07327B-s2847

RA-009-C9RA07327B-s2848

RA-009-C9RA07327B-s2849

RA-009-C9RA07327B-s2850

RA-009-C9RA07327B-s2851

RA-009-C9RA07327B-s2852

RA-009-C9RA07327B-s2853

RA-009-C9RA07327B-s2854

RA-009-C9RA07327B-s2855

RA-009-C9RA07327B-s2856

RA-009-C9RA07327B-s2857

RA-009-C9RA07327B-s2858

RA-009-C9RA07327B-s2859

RA-009-C9RA07327B-s2860

RA-009-C9RA07327B-s2861

RA-009-C9RA07327B-s2862

RA-009-C9RA07327B-s2863

RA-009-C9RA07327B-s2864

RA-009-C9RA07327B-s2865

RA-009-C9RA07327B-s2866

RA-009-C9RA07327B-s2867

RA-009-C9RA07327B-s2868

RA-009-C9RA07327B-s2869

RA-009-C9RA07327B-s2870

RA-009-C9RA07327B-s2871

RA-009-C9RA07327B-s2872

RA-009-C9RA07327B-s2873

RA-009-C9RA07327B-s2874

RA-009-C9RA07327B-s2875

RA-009-C9RA07327B-s2876

RA-009-C9RA07327B-s2877

RA-009-C9RA07327B-s2878

RA-009-C9RA07327B-s2879

RA-009-C9RA07327B-s2880

RA-009-C9RA07327B-s2881

RA-009-C9RA07327B-s2882

RA-009-C9RA07327B-s2883

RA-009-C9RA07327B-s2884

RA-009-C9RA07327B-s2885

RA-009-C9RA07327B-s2886

RA-009-C9RA07327B-s2887

RA-009-C9RA07327B-s2888

RA-009-C9RA07327B-s2889

RA-009-C9RA07327B-s2890

RA-009-C9RA07327B-s2891

RA-009-C9RA07327B-s2892

RA-009-C9RA07327B-s2893

RA-009-C9RA07327B-s2894

RA-009-C9RA07327B-s2895

RA-009-C9RA07327B-s2896

RA-009-C9RA07327B-s2897

RA-009-C9RA07327B-s2898

RA-009-C9RA07327B-s2899

RA-009-C9RA07327B-s2900

RA-009-C9RA07327B-s2901

RA-009-C9RA07327B-s2902

RA-009-C9RA07327B-s2903

RA-009-C9RA07327B-s2904

RA-009-C9RA07327B-s2905

RA-009-C9RA07327B-s2906

RA-009-C9RA07327B-s2907

RA-009-C9RA07327B-s2908

RA-009-C9RA07327B-s2909

RA-009-C9RA07327B-s2910

RA-009-C9RA07327B-s2911

RA-009-C9RA07327B-s2912

RA-009-C9RA07327B-s2913

RA-009-C9RA07327B-s2914

RA-009-C9RA07327B-s2915

RA-009-C9RA07327B-s2916

RA-009-C9RA07327B-s2917

RA-009-C9RA07327B-s2918

RA-009-C9RA07327B-s2919

RA-009-C9RA07327B-s2920

RA-009-C9RA07327B-s2921

RA-009-C9RA07327B-s2922

RA-009-C9RA07327B-s2923

RA-009-C9RA07327B-s2924

RA-009-C9RA07327B-s2925

RA-009-C9RA07327B-s2926

RA-009-C9RA07327B-s2927

RA-009-C9RA07327B-s2928

RA-009-C9RA07327B-s2929

RA-009-C9RA07327B-s2930

RA-009-C9RA07327B-s2931

RA-009-C9RA07327B-s2932

RA-009-C9RA07327B-s2933

RA-009-C9RA07327B-s2934

RA-009-C9RA07327B-s2935

RA-009-C9RA07327B-s2936

RA-009-C9RA07327B-s2937

RA-009-C9RA07327B-s2938

RA-009-C9RA07327B-s2939

RA-009-C9RA07327B-s2940

RA-009-C9RA07327B-s2941

RA-009-C9RA07327B-s2942

RA-009-C9RA07327B-s2943

RA-009-C9RA07327B-s2944

RA-009-C9RA07327B-s2945

RA-009-C9RA07327B-s2946

RA-009-C9RA07327B-s2947

RA-009-C9RA07327B-s2948

RA-009-C9RA07327B-s2949

RA-009-C9RA07327B-s2950

RA-009-C9RA07327B-s2951

RA-009-C9RA07327B-s2952

RA-009-C9RA07327B-s2953

RA-009-C9RA07327B-s2954

RA-009-C9RA07327B-s2955

RA-009-C9RA07327B-s2956

RA-009-C9RA07327B-s2957

RA-009-C9RA07327B-s2958

RA-009-C9RA07327B-s2959

RA-009-C9RA07327B-s2960

RA-009-C9RA07327B-s2961

RA-009-C9RA07327B-s2962

RA-009-C9RA07327B-s2963

RA-009-C9RA07327B-s2964

RA-009-C9RA07327B-s2965

RA-009-C9RA07327B-s2966

RA-009-C9RA07327B-s2967

RA-009-C9RA07327B-s2968

RA-009-C9RA07327B-s2969

RA-009-C9RA07327B-s2970

RA-009-C9RA07327B-s2971

RA-009-C9RA07327B-s2972

RA-009-C9RA07327B-s2973

RA-009-C9RA07327B-s2974

RA-009-C9RA07327B-s2975

RA-009-C9RA07327B-s2976

RA-009-C9RA07327B-s2977

RA-009-C9RA07327B-s2978

RA-009-C9RA07327B-s2979

RA-009-C9RA07327B-s2980

RA-009-C9RA07327B-s2981

RA-009-C9RA07327B-s2982

RA-009-C9RA07327B-s2983

RA-009-C9RA07327B-s2984

RA-009-C9RA07327B-s2985

RA-009-C9RA07327B-s2986

RA-009-C9RA07327B-s2987

RA-009-C9RA07327B-s2988

RA-009-C9RA07327B-s2989

RA-009-C9RA07327B-s2990

RA-009-C9RA07327B-s2991

RA-009-C9RA07327B-s2992

RA-009-C9RA07327B-s2993

RA-009-C9RA07327B-s2994

RA-009-C9RA07327B-s2995

RA-009-C9RA07327B-s2996

RA-009-C9RA07327B-s2997

RA-009-C9RA07327B-s2998

RA-009-C9RA07327B-s2999

RA-009-C9RA07327B-s3000

RA-009-C9RA07327B-s3001

RA-009-C9RA07327B-s3002

RA-009-C9RA07327B-s3003

RA-009-C9RA07327B-s3004

RA-009-C9RA07327B-s3005

RA-009-C9RA07327B-s3006

RA-009-C9RA07327B-s3007

RA-009-C9RA07327B-s3008

RA-009-C9RA07327B-s3009

RA-009-C9RA07327B-s3010

RA-009-C9RA07327B-s3011

RA-009-C9RA07327B-s3012

RA-009-C9RA07327B-s3013

RA-009-C9RA07327B-s3014

RA-009-C9RA07327B-s3015

RA-009-C9RA07327B-s3016

RA-009-C9RA07327B-s3017

RA-009-C9RA07327B-s3018

RA-009-C9RA07327B-s3019

RA-009-C9RA07327B-s3020

RA-009-C9RA07327B-s3021

RA-009-C9RA07327B-s3022

RA-009-C9RA07327B-s3023

RA-009-C9RA07327B-s3024

RA-009-C9RA07327B-s3025

RA-009-C9RA07327B-s3026

RA-009-C9RA07327B-s3027

RA-009-C9RA07327B-s3028

RA-009-C9RA07327B-s3029

RA-009-C9RA07327B-s3030

RA-009-C9RA07327B-s3031

RA-009-C9RA07327B-s3032

RA-009-C9RA07327B-s3033

RA-009-C9RA07327B-s3034

RA-009-C9RA07327B-s3035

RA-009-C9RA07327B-s3036

RA-009-C9RA07327B-s3037

RA-009-C9RA07327B-s3038

RA-009-C9RA07327B-s3039

RA-009-C9RA07327B-s3040

RA-009-C9RA07327B-s3041

RA-009-C9RA07327B-s3042

RA-009-C9RA07327B-s3043

RA-009-C9RA07327B-s3044

RA-009-C9RA07327B-s3045

RA-009-C9RA07327B-s3046

RA-009-C9RA07327B-s3047

RA-009-C9RA07327B-s3048

RA-009-C9RA07327B-s3049

RA-009-C9RA07327B-s3050

RA-009-C9RA07327B-s3051

RA-009-C9RA07327B-s3052

RA-009-C9RA07327B-s3053

RA-009-C9RA07327B-s3054

RA-009-C9RA07327B-s3055

RA-009-C9RA07327B-s3056

RA-009-C9RA07327B-s3057

RA-009-C9RA07327B-s3058

RA-009-C9RA07327B-s3059

RA-009-C9RA07327B-s3060

RA-009-C9RA07327B-s3061

RA-009-C9RA07327B-s3062

RA-009-C9RA07327B-s3063

RA-009-C9RA07327B-s3064

RA-009-C9RA07327B-s3065

RA-009-C9RA07327B-s3066

RA-009-C9RA07327B-s3067

RA-009-C9RA07327B-s3068

RA-009-C9RA07327B-s3069

RA-009-C9RA07327B-s3070

RA-009-C9RA07327B-s3071

RA-009-C9RA07327B-s3072

RA-009-C9RA07327B-s3073

RA-009-C9RA07327B-s3074

RA-009-C9RA07327B-s3075

RA-009-C9RA07327B-s3076

RA-009-C9RA07327B-s3077

RA-009-C9RA07327B-s3078

RA-009-C9RA07327B-s3079

RA-009-C9RA07327B-s3080

RA-009-C9RA07327B-s3081

RA-009-C9RA07327B-s3082

RA-009-C9RA07327B-s3083

RA-009-C9RA07327B-s3084

RA-009-C9RA07327B-s3085

RA-009-C9RA07327B-s3086

RA-009-C9RA07327B-s3087

RA-009-C9RA07327B-s3088

RA-009-C9RA07327B-s3089

RA-009-C9RA07327B-s3090

RA-009-C9RA07327B-s3091

RA-009-C9RA07327B-s3092

RA-009-C9RA07327B-s3093

RA-009-C9RA07327B-s3094

RA-009-C9RA07327B-s3095

RA-009-C9RA07327B-s3096

RA-009-C9RA07327B-s3097

RA-009-C9RA07327B-s3098

RA-009-C9RA07327B-s3099

RA-009-C9RA07327B-s3100

RA-009-C9RA07327B-s3101

RA-009-C9RA07327B-s3102

RA-009-C9RA07327B-s3103

RA-009-C9RA07327B-s3104

RA-009-C9RA07327B-s3105

RA-009-C9RA07327B-s3106

RA-009-C9RA07327B-s3107

RA-009-C9RA07327B-s3108

RA-009-C9RA07327B-s3109

RA-009-C9RA07327B-s3110

RA-009-C9RA07327B-s3111

RA-009-C9RA07327B-s3112

RA-009-C9RA07327B-s3113

RA-009-C9RA07327B-s3114

RA-009-C9RA07327B-s3115

RA-009-C9RA07327B-s3116

RA-009-C9RA07327B-s3117

RA-009-C9RA07327B-s3118

RA-009-C9RA07327B-s3119

RA-009-C9RA07327B-s3120

RA-009-C9RA07327B-s3121

RA-009-C9RA07327B-s3122

RA-009-C9RA07327B-s3123

RA-009-C9RA07327B-s3124

RA-009-C9RA07327B-s3125

RA-009-C9RA07327B-s3126

RA-009-C9RA07327B-s3127

RA-009-C9RA07327B-s3128

RA-009-C9RA07327B-s3129

RA-009-C9RA07327B-s3130

RA-009-C9RA07327B-s3131

RA-009-C9RA07327B-s3132

RA-009-C9RA07327B-s3133

RA-009-C9RA07327B-s3134

RA-009-C9RA07327B-s3135

RA-009-C9RA07327B-s3136

RA-009-C9RA07327B-s3137

RA-009-C9RA07327B-s3138

RA-009-C9RA07327B-s3139

RA-009-C9RA07327B-s3140

RA-009-C9RA07327B-s3141

RA-009-C9RA07327B-s3142

RA-009-C9RA07327B-s3143

RA-009-C9RA07327B-s3144

RA-009-C9RA07327B-s3145

RA-009-C9RA07327B-s3146

RA-009-C9RA07327B-s3147

RA-009-C9RA07327B-s3148

RA-009-C9RA07327B-s3149

RA-009-C9RA07327B-s3150

RA-009-C9RA07327B-s3151

RA-009-C9RA07327B-s3152

RA-009-C9RA07327B-s3153

RA-009-C9RA07327B-s3154

RA-009-C9RA07327B-s3155

RA-009-C9RA07327B-s3156

RA-009-C9RA07327B-s3157

RA-009-C9RA07327B-s3158

RA-009-C9RA07327B-s3159

RA-009-C9RA07327B-s3160

RA-009-C9RA07327B-s3161

RA-009-C9RA07327B-s3162

RA-009-C9RA07327B-s3163

RA-009-C9RA07327B-s3164

RA-009-C9RA07327B-s3165

RA-009-C9RA07327B-s3166

RA-009-C9RA07327B-s3167

RA-009-C9RA07327B-s3168

RA-009-C9RA07327B-s3169

RA-009-C9RA07327B-s3170

RA-009-C9RA07327B-s3171

RA-009-C9RA07327B-s3172

RA-009-C9RA07327B-s3173

RA-009-C9RA07327B-s3174

RA-009-C9RA07327B-s3175

RA-009-C9RA07327B-s3176

RA-009-C9RA07327B-s3177

RA-009-C9RA07327B-s3178

RA-009-C9RA07327B-s3179

RA-009-C9RA07327B-s3180

RA-009-C9RA07327B-s3181

RA-009-C9RA07327B-s3182

RA-009-C9RA07327B-s3183

RA-009-C9RA07327B-s3184

RA-009-C9RA07327B-s3185

RA-009-C9RA07327B-s3186

RA-009-C9RA07327B-s3187

RA-009-C9RA07327B-s3188

RA-009-C9RA07327B-s3189

RA-009-C9RA07327B-s3190

RA-009-C9RA07327B-s3191

RA-009-C9RA07327B-s3192

RA-009-C9RA07327B-s3193

RA-009-C9RA07327B-s3194

RA-009-C9RA07327B-s3195

RA-009-C9RA07327B-s3196

RA-009-C9RA07327B-s3197

RA-009-C9RA07327B-s3198

RA-009-C9RA07327B-s3199

RA-009-C9RA07327B-s3200

RA-009-C9RA07327B-s3201

RA-009-C9RA07327B-s3202

RA-009-C9RA07327B-s3203

RA-009-C9RA07327B-s3204

RA-009-C9RA07327B-s3205

RA-009-C9RA07327B-s3206

RA-009-C9RA07327B-s3207

RA-009-C9RA07327B-s3208

RA-009-C9RA07327B-s3209

RA-009-C9RA07327B-s3210

RA-009-C9RA07327B-s3211

RA-009-C9RA07327B-s3212

RA-009-C9RA07327B-s3213

RA-009-C9RA07327B-s3214

RA-009-C9RA07327B-s3215

RA-009-C9RA07327B-s3216

RA-009-C9RA07327B-s3217

RA-009-C9RA07327B-s3218

RA-009-C9RA07327B-s3219

RA-009-C9RA07327B-s3220

RA-009-C9RA07327B-s3221

RA-009-C9RA07327B-s3222

RA-009-C9RA07327B-s3223

RA-009-C9RA07327B-s3224

RA-009-C9RA07327B-s3225

RA-009-C9RA07327B-s3226

RA-009-C9RA07327B-s3227

RA-009-C9RA07327B-s3228

RA-009-C9RA07327B-s3229

RA-009-C9RA07327B-s3230

RA-009-C9RA07327B-s3231

RA-009-C9RA07327B-s3232

RA-009-C9RA07327B-s3233

RA-009-C9RA07327B-s3234

RA-009-C9RA07327B-s3235

RA-009-C9RA07327B-s3236

RA-009-C9RA07327B-s3237

RA-009-C9RA07327B-s3238

RA-009-C9RA07327B-s3239

RA-009-C9RA07327B-s3240

RA-009-C9RA07327B-s3241

RA-009-C9RA07327B-s3242

RA-009-C9RA07327B-s3243

RA-009-C9RA07327B-s3244

RA-009-C9RA07327B-s3245

RA-009-C9RA07327B-s3246

RA-009-C9RA07327B-s3247

RA-009-C9RA07327B-s3248

RA-009-C9RA07327B-s3249

RA-009-C9RA07327B-s3250

RA-009-C9RA07327B-s3251

RA-009-C9RA07327B-s3252

RA-009-C9RA07327B-s3253

RA-009-C9RA07327B-s3254

RA-009-C9RA07327B-s3255

RA-009-C9RA07327B-s3256

RA-009-C9RA07327B-s3257

RA-009-C9RA07327B-s3258

RA-009-C9RA07327B-s3259

RA-009-C9RA07327B-s3260

RA-009-C9RA07327B-s3261

RA-009-C9RA07327B-s3262

RA-009-C9RA07327B-s3263

RA-009-C9RA07327B-s3264

RA-009-C9RA07327B-s3265

RA-009-C9RA07327B-s3266

RA-009-C9RA07327B-s3267

RA-009-C9RA07327B-s3268

RA-009-C9RA07327B-s3269

RA-009-C9RA07327B-s3270

RA-009-C9RA07327B-s3271

RA-009-C9RA07327B-s3272

RA-009-C9RA07327B-s3273

RA-009-C9RA07327B-s3274

RA-009-C9RA07327B-s3275

RA-009-C9RA07327B-s3276

RA-009-C9RA07327B-s3277

RA-009-C9RA07327B-s3278

RA-009-C9RA07327B-s3279

RA-009-C9RA07327B-s3280

RA-009-C9RA07327B-s3281

RA-009-C9RA07327B-s3282

RA-009-C9RA07327B-s3283

RA-009-C9RA07327B-s3284

RA-009-C9RA07327B-s3285

RA-009-C9RA07327B-s3286

RA-009-C9RA07327B-s3287

RA-009-C9RA07327B-s3288

RA-009-C9RA07327B-s3289

RA-009-C9RA07327B-s3290

RA-009-C9RA07327B-s3291

RA-009-C9RA07327B-s3292

RA-009-C9RA07327B-s3293

RA-009-C9RA07327B-s3294

RA-009-C9RA07327B-s3295

RA-009-C9RA07327B-s3296

RA-009-C9RA07327B-s3297

RA-009-C9RA07327B-s3298

RA-009-C9RA07327B-s3299

RA-009-C9RA07327B-s3300

RA-009-C9RA07327B-s3301

RA-009-C9RA07327B-s3302

RA-009-C9RA07327B-s3303

RA-009-C9RA07327B-s3304

RA-009-C9RA07327B-s3305

RA-009-C9RA07327B-s3306

RA-009-C9RA07327B-s3307

RA-009-C9RA07327B-s3308

RA-009-C9RA07327B-s3309

RA-009-C9RA07327B-s3310

RA-009-C9RA07327B-s3311

RA-009-C9RA07327B-s3312

RA-009-C9RA07327B-s3313

RA-009-C9RA07327B-s3314

RA-009-C9RA07327B-s3315

RA-009-C9RA07327B-s3316

RA-009-C9RA07327B-s3317

RA-009-C9RA07327B-s3318

RA-009-C9RA07327B-s3319

RA-009-C9RA07327B-s3320

RA-009-C9RA07327B-s3321

RA-009-C9RA07327B-s3322

RA-009-C9RA07327B-s3323

RA-009-C9RA07327B-s3324

RA-009-C9RA07327B-s3325

RA-009-C9RA07327B-s3326

RA-009-C9RA07327B-s3327

RA-009-C9RA07327B-s3328

RA-009-C9RA07327B-s3329

RA-009-C9RA07327B-s3330

RA-009-C9RA07327B-s3331

RA-009-C9RA07327B-s3332

RA-009-C9RA07327B-s3333

RA-009-C9RA07327B-s3334

RA-009-C9RA07327B-s3335

RA-009-C9RA07327B-s3336

RA-009-C9RA07327B-s3337

RA-009-C9RA07327B-s3338

RA-009-C9RA07327B-s3339

RA-009-C9RA07327B-s3340

RA-009-C9RA07327B-s3341

RA-009-C9RA07327B-s3342

RA-009-C9RA07327B-s3343

RA-009-C9RA07327B-s3344

RA-009-C9RA07327B-s3345

RA-009-C9RA07327B-s3346

RA-009-C9RA07327B-s3347

RA-009-C9RA07327B-s3348

RA-009-C9RA07327B-s3349

RA-009-C9RA07327B-s3350

RA-009-C9RA07327B-s3351

RA-009-C9RA07327B-s3352

RA-009-C9RA07327B-s3353

RA-009-C9RA07327B-s3354

RA-009-C9RA07327B-s3355

RA-009-C9RA07327B-s3356

RA-009-C9RA07327B-s3357

RA-009-C9RA07327B-s3358

RA-009-C9RA07327B-s3359

RA-009-C9RA07327B-s3360

RA-009-C9RA07327B-s3361

RA-009-C9RA07327B-s3362

RA-009-C9RA07327B-s3363

RA-009-C9RA07327B-s3364

RA-009-C9RA07327B-s3365

RA-009-C9RA07327B-s3366

RA-009-C9RA07327B-s3367

RA-009-C9RA07327B-s3368

RA-009-C9RA07327B-s3369

RA-009-C9RA07327B-s3370

RA-009-C9RA07327B-s3371

RA-009-C9RA07327B-s3372

RA-009-C9RA07327B-s3373

RA-009-C9RA07327B-s3374

RA-009-C9RA07327B-s3375

RA-009-C9RA07327B-s3376

RA-009-C9RA07327B-s3377

RA-009-C9RA07327B-s3378

RA-009-C9RA07327B-s3379

RA-009-C9RA07327B-s3380

RA-009-C9RA07327B-s3381

RA-009-C9RA07327B-s3382

RA-009-C9RA07327B-s3383

RA-009-C9RA07327B-s3384

RA-009-C9RA07327B-s3385

RA-009-C9RA07327B-s3386

RA-009-C9RA07327B-s3387

RA-009-C9RA07327B-s3388

RA-009-C9RA07327B-s3389

RA-009-C9RA07327B-s3390

RA-009-C9RA07327B-s3391

RA-009-C9RA07327B-s3392

RA-009-C9RA07327B-s3393

RA-009-C9RA07327B-s3394

RA-009-C9RA07327B-s3395

RA-009-C9RA07327B-s3396

RA-009-C9RA07327B-s3397

RA-009-C9RA07327B-s3398

RA-009-C9RA07327B-s3399

RA-009-C9RA07327B-s3400

RA-009-C9RA07327B-s3401

RA-009-C9RA07327B-s3402

RA-009-C9RA07327B-s3403

RA-009-C9RA07327B-s3404

RA-009-C9RA07327B-s3405

RA-009-C9RA07327B-s3406

RA-009-C9RA07327B-s3407

RA-009-C9RA07327B-s3408

RA-009-C9RA07327B-s3409

RA-009-C9RA07327B-s3410

RA-009-C9RA07327B-s3411

RA-009-C9RA07327B-s3412

RA-009-C9RA07327B-s3413

RA-009-C9RA07327B-s3414

RA-009-C9RA07327B-s3415

RA-009-C9RA07327B-s3416

RA-009-C9RA07327B-s3417

RA-009-C9RA07327B-s3418

RA-009-C9RA07327B-s3419

RA-009-C9RA07327B-s3420

RA-009-C9RA07327B-s3421

RA-009-C9RA07327B-s3422

RA-009-C9RA07327B-s3423

RA-009-C9RA07327B-s3424

RA-009-C9RA07327B-s3425

RA-009-C9RA07327B-s3426

RA-009-C9RA07327B-s3427

RA-009-C9RA07327B-s3428

RA-009-C9RA07327B-s3429

RA-009-C9RA07327B-s3430

RA-009-C9RA07327B-s3431

RA-009-C9RA07327B-s3432

RA-009-C9RA07327B-s3433

RA-009-C9RA07327B-s3434

RA-009-C9RA07327B-s3435

RA-009-C9RA07327B-s3436

RA-009-C9RA07327B-s3437

RA-009-C9RA07327B-s3438

RA-009-C9RA07327B-s3439

RA-009-C9RA07327B-s3440

RA-009-C9RA07327B-s3441

RA-009-C9RA07327B-s3442

RA-009-C9RA07327B-s3443

RA-009-C9RA07327B-s3444

RA-009-C9RA07327B-s3445

RA-009-C9RA07327B-s3446

RA-009-C9RA07327B-s3447

RA-009-C9RA07327B-s3448

RA-009-C9RA07327B-s3449

RA-009-C9RA07327B-s3450

RA-009-C9RA07327B-s3451

RA-009-C9RA07327B-s3452

RA-009-C9RA07327B-s3453

RA-009-C9RA07327B-s3454

RA-009-C9RA07327B-s3455

RA-009-C9RA07327B-s3456

RA-009-C9RA07327B-s3457

RA-009-C9RA07327B-s3458

RA-009-C9RA07327B-s3459

RA-009-C9RA07327B-s3460

RA-009-C9RA07327B-s3461

RA-009-C9RA07327B-s3462

RA-009-C9RA07327B-s3463

RA-009-C9RA07327B-s3464

RA-009-C9RA07327B-s3465

RA-009-C9RA07327B-s3466

RA-009-C9RA07327B-s3467

RA-009-C9RA07327B-s3468

RA-009-C9RA07327B-s3469

RA-009-C9RA07327B-s3470

RA-009-C9RA07327B-s3471

RA-009-C9RA07327B-s3472

RA-009-C9RA07327B-s3473

RA-009-C9RA07327B-s3474

RA-009-C9RA07327B-s3475

RA-009-C9RA07327B-s3476

RA-009-C9RA07327B-s3477

RA-009-C9RA07327B-s3478

RA-009-C9RA07327B-s3479

RA-009-C9RA07327B-s3480

RA-009-C9RA07327B-s3481

RA-009-C9RA07327B-s3482

RA-009-C9RA07327B-s3483

RA-009-C9RA07327B-s3484

RA-009-C9RA07327B-s3485

RA-009-C9RA07327B-s3486

RA-009-C9RA07327B-s3487

RA-009-C9RA07327B-s3488

RA-009-C9RA07327B-s3489

RA-009-C9RA07327B-s3490

RA-009-C9RA07327B-s3491

RA-009-C9RA07327B-s3492

RA-009-C9RA07327B-s3493

RA-009-C9RA07327B-s3494

RA-009-C9RA07327B-s3495

RA-009-C9RA07327B-s3496

RA-009-C9RA07327B-s3497

RA-009-C9RA07327B-s3498

RA-009-C9RA07327B-s3499

RA-009-C9RA07327B-s3500

RA-009-C9RA07327B-s3501

RA-009-C9RA07327B-s3502

RA-009-C9RA07327B-s3503

RA-009-C9RA07327B-s3504

RA-009-C9RA07327B-s3505

RA-009-C9RA07327B-s3506

RA-009-C9RA07327B-s3507

RA-009-C9RA07327B-s3508

RA-009-C9RA07327B-s3509

RA-009-C9RA07327B-s3510

RA-009-C9RA07327B-s3511

RA-009-C9RA07327B-s3512

RA-009-C9RA07327B-s3513

RA-009-C9RA07327B-s3514

RA-009-C9RA07327B-s3515

RA-009-C9RA07327B-s3516

RA-009-C9RA07327B-s3517

RA-009-C9RA07327B-s3518

RA-009-C9RA07327B-s3519

RA-009-C9RA07327B-s3520

RA-009-C9RA07327B-s3521

RA-009-C9RA07327B-s3522

RA-009-C9RA07327B-s3523

RA-009-C9RA07327B-s3524

RA-009-C9RA07327B-s3525

RA-009-C9RA07327B-s3526

RA-009-C9RA07327B-s3527

RA-009-C9RA07327B-s3528

RA-009-C9RA07327B-s3529

RA-009-C9RA07327B-s3530

RA-009-C9RA07327B-s3531

RA-009-C9RA07327B-s3532

RA-009-C9RA07327B-s3533

RA-009-C9RA07327B-s3534

RA-009-C9RA07327B-s3535

RA-009-C9RA07327B-s3536

RA-009-C9RA07327B-s3537

RA-009-C9RA07327B-s3538

RA-009-C9RA07327B-s3539

RA-009-C9RA07327B-s3540

RA-009-C9RA07327B-s3541

RA-009-C9RA07327B-s3542

RA-009-C9RA07327B-s3543

RA-009-C9RA07327B-s3544

RA-009-C9RA07327B-s3545

RA-009-C9RA07327B-s3546

RA-009-C9RA07327B-s3547

RA-009-C9RA07327B-s3548

RA-009-C9RA07327B-s3549

RA-009-C9RA07327B-s3550

RA-009-C9RA07327B-s3551

RA-009-C9RA07327B-s3552

RA-009-C9RA07327B-s3553

RA-009-C9RA07327B-s3554

RA-009-C9RA07327B-s3555

RA-009-C9RA07327B-s3556

RA-009-C9RA07327B-s3557

RA-009-C9RA07327B-s3558

RA-009-C9RA07327B-s3559

RA-009-C9RA07327B-s3560

RA-009-C9RA07327B-s3561

RA-009-C9RA07327B-s3562

RA-009-C9RA07327B-s3563

RA-009-C9RA07327B-s3564

RA-009-C9RA07327B-s3565

RA-009-C9RA07327B-s3566

RA-009-C9RA07327B-s3567

RA-009-C9RA07327B-s3568

RA-009-C9RA07327B-s3569

RA-009-C9RA07327B-s3570

RA-009-C9RA07327B-s3571

RA-009-C9RA07327B-s3572

RA-009-C9RA07327B-s3573

RA-009-C9RA07327B-s3574

RA-009-C9RA07327B-s3575

RA-009-C9RA07327B-s3576

RA-009-C9RA07327B-s3577

RA-009-C9RA07327B-s3578

RA-009-C9RA07327B-s3579

RA-009-C9RA07327B-s3580

RA-009-C9RA07327B-s3581

RA-009-C9RA07327B-s3582

RA-009-C9RA07327B-s3583

RA-009-C9RA07327B-s3584

RA-009-C9RA07327B-s3585

RA-009-C9RA07327B-s3586

RA-009-C9RA07327B-s3587

RA-009-C9RA07327B-s3588

RA-009-C9RA07327B-s3589

RA-009-C9RA07327B-s3590

RA-009-C9RA07327B-s3591

RA-009-C9RA07327B-s3592

RA-009-C9RA07327B-s3593

RA-009-C9RA07327B-s3594

RA-009-C9RA07327B-s3595

RA-009-C9RA07327B-s3596

RA-009-C9RA07327B-s3597

RA-009-C9RA07327B-s3598

RA-009-C9RA07327B-s3599

RA-009-C9RA07327B-s3600

RA-009-C9RA07327B-s3601

RA-009-C9RA07327B-s3602

RA-009-C9RA07327B-s3603

RA-009-C9RA07327B-s3604

RA-009-C9RA07327B-s3605

RA-009-C9RA07327B-s3606

RA-009-C9RA07327B-s3607

RA-009-C9RA07327B-s3608

RA-009-C9RA07327B-s3609

RA-009-C9RA07327B-s3610

RA-009-C9RA07327B-s3611

RA-009-C9RA07327B-s3612

RA-009-C9RA07327B-s3613

RA-009-C9RA07327B-s3614

RA-009-C9RA07327B-s3615

RA-009-C9RA07327B-s3616

RA-009-C9RA07327B-s3617

RA-009-C9RA07327B-s3618

RA-009-C9RA07327B-s3619

RA-009-C9RA07327B-s3620

RA-009-C9RA07327B-s3621

RA-009-C9RA07327B-s3622

RA-009-C9RA07327B-s3623

RA-009-C9RA07327B-s3624

RA-009-C9RA07327B-s3625

RA-009-C9RA07327B-s3626

RA-009-C9RA07327B-s3627

RA-009-C9RA07327B-s3628

RA-009-C9RA07327B-s3629

RA-009-C9RA07327B-s3630

RA-009-C9RA07327B-s3631

RA-009-C9RA07327B-s3632

RA-009-C9RA07327B-s3633

RA-009-C9RA07327B-s3634

RA-009-C9RA07327B-s3635

RA-009-C9RA07327B-s3636

RA-009-C9RA07327B-s3637

RA-009-C9RA07327B-s3638

RA-009-C9RA07327B-s3639

RA-009-C9RA07327B-s3640

RA-009-C9RA07327B-s3641

RA-009-C9RA07327B-s3642

RA-009-C9RA07327B-s3643

RA-009-C9RA07327B-s3644

RA-009-C9RA07327B-s3645

RA-009-C9RA07327B-s3646

RA-009-C9RA07327B-s3647

RA-009-C9RA07327B-s3648

RA-009-C9RA07327B-s3649

RA-009-C9RA07327B-s3650

RA-009-C9RA07327B-s3651

RA-009-C9RA07327B-s3652

RA-009-C9RA07327B-s3653

RA-009-C9RA07327B-s3654

RA-009-C9RA07327B-s3655

RA-009-C9RA07327B-s3656

RA-009-C9RA07327B-s3657

RA-009-C9RA07327B-s3658

RA-009-C9RA07327B-s3659

RA-009-C9RA07327B-s3660

RA-009-C9RA07327B-s3661

RA-009-C9RA07327B-s3662

RA-009-C9RA07327B-s3663

RA-009-C9RA07327B-s3664

RA-009-C9RA07327B-s3665

RA-009-C9RA07327B-s3666

RA-009-C9RA07327B-s3667

RA-009-C9RA07327B-s3668

RA-009-C9RA07327B-s3669

RA-009-C9RA07327B-s3670

RA-009-C9RA07327B-s3671

RA-009-C9RA07327B-s3672

RA-009-C9RA07327B-s3673

RA-009-C9RA07327B-s3674

RA-009-C9RA07327B-s3675

RA-009-C9RA07327B-s3676

RA-009-C9RA07327B-s3677

RA-009-C9RA07327B-s3678

RA-009-C9RA07327B-s3679

RA-009-C9RA07327B-s3680

RA-009-C9RA07327B-s3681

RA-009-C9RA07327B-s3682

RA-009-C9RA07327B-s3683

RA-009-C9RA07327B-s3684

RA-009-C9RA07327B-s3685

RA-009-C9RA07327B-s3686

RA-009-C9RA07327B-s3687

RA-009-C9RA07327B-s3688

RA-009-C9RA07327B-s3689

RA-009-C9RA07327B-s3690

RA-009-C9RA07327B-s3691

RA-009-C9RA07327B-s3692

RA-009-C9RA07327B-s3693

RA-009-C9RA07327B-s3694

RA-009-C9RA07327B-s3695

RA-009-C9RA07327B-s3696

RA-009-C9RA07327B-s3697

RA-009-C9RA07327B-s3698

RA-009-C9RA07327B-s3699

RA-009-C9RA07327B-s3700

RA-009-C9RA07327B-s3701

RA-009-C9RA07327B-s3702

RA-009-C9RA07327B-s3703

RA-009-C9RA07327B-s3704

RA-009-C9RA07327B-s3705

RA-009-C9RA07327B-s3706

RA-009-C9RA07327B-s3707

RA-009-C9RA07327B-s3708

RA-009-C9RA07327B-s3709

RA-009-C9RA07327B-s3710

RA-009-C9RA07327B-s3711

RA-009-C9RA07327B-s3712

RA-009-C9RA07327B-s3713

RA-009-C9RA07327B-s3714

RA-009-C9RA07327B-s3715

RA-009-C9RA07327B-s3716

RA-009-C9RA07327B-s3717

RA-009-C9RA07327B-s3718

RA-009-C9RA07327B-s3719

RA-009-C9RA07327B-s3720

RA-009-C9RA07327B-s3721

RA-009-C9RA07327B-s3722

RA-009-C9RA07327B-s3723

RA-009-C9RA07327B-s3724

RA-009-C9RA07327B-s3725

RA-009-C9RA07327B-s3726

RA-009-C9RA07327B-s3727

RA-009-C9RA07327B-s3728

RA-009-C9RA07327B-s3729

RA-009-C9RA07327B-s3730

RA-009-C9RA07327B-s3731

RA-009-C9RA07327B-s3732

RA-009-C9RA07327B-s3733

RA-009-C9RA07327B-s3734

RA-009-C9RA07327B-s3735

RA-009-C9RA07327B-s3736

RA-009-C9RA07327B-s3737

RA-009-C9RA07327B-s3738

RA-009-C9RA07327B-s3739

RA-009-C9RA07327B-s3740

RA-009-C9RA07327B-s3741

RA-009-C9RA07327B-s3742

RA-009-C9RA07327B-s3743

RA-009-C9RA07327B-s3744

RA-009-C9RA07327B-s3745

RA-009-C9RA07327B-s3746

RA-009-C9RA07327B-s3747

RA-009-C9RA07327B-s3748

RA-009-C9RA07327B-s3749

RA-009-C9RA07327B-s3750

RA-009-C9RA07327B-s3751

RA-009-C9RA07327B-s3752

RA-009-C9RA07327B-s3753

RA-009-C9RA07327B-s3754

RA-009-C9RA07327B-s3755

RA-009-C9RA07327B-s3756

RA-009-C9RA07327B-s3757

RA-009-C9RA07327B-s3758

RA-009-C9RA07327B-s3759

RA-009-C9RA07327B-s3760

RA-009-C9RA07327B-s3761

RA-009-C9RA07327B-s3762

RA-009-C9RA07327B-s3763

RA-009-C9RA07327B-s3764

RA-009-C9RA07327B-s3765

RA-009-C9RA07327B-s3766

RA-009-C9RA07327B-s3767

RA-009-C9RA07327B-s3768

RA-009-C9RA07327B-s3769

RA-009-C9RA07327B-s3770

RA-009-C9RA07327B-s3771

RA-009-C9RA07327B-s3772

RA-009-C9RA07327B-s3773

RA-009-C9RA07327B-s3774

RA-009-C9RA07327B-s3775

RA-009-C9RA07327B-s3776

RA-009-C9RA07327B-s3777

RA-009-C9RA07327B-s3778

RA-009-C9RA07327B-s3779

RA-009-C9RA07327B-s3780

RA-009-C9RA07327B-s3781

RA-009-C9RA07327B-s3782

RA-009-C9RA07327B-s3783

RA-009-C9RA07327B-s3784

RA-009-C9RA07327B-s3785

RA-009-C9RA07327B-s3786

RA-009-C9RA07327B-s3787

RA-009-C9RA07327B-s3788

RA-009-C9RA07327B-s3789

RA-009-C9RA07327B-s3790

RA-009-C9RA07327B-s3791

RA-009-C9RA07327B-s3792

RA-009-C9RA07327B-s3793

RA-009-C9RA07327B-s3794

RA-009-C9RA07327B-s3795

RA-009-C9RA07327B-s3796

RA-009-C9RA07327B-s3797

RA-009-C9RA07327B-s3798

RA-009-C9RA07327B-s3799

RA-009-C9RA07327B-s3800

RA-009-C9RA07327B-s3801

RA-009-C9RA07327B-s3802

RA-009-C9RA07327B-s3803

RA-009-C9RA07327B-s3804

RA-009-C9RA07327B-s3805

RA-009-C9RA07327B-s3806

RA-009-C9RA07327B-s3807

RA-009-C9RA07327B-s3808

RA-009-C9RA07327B-s3809

RA-009-C9RA07327B-s3810

RA-009-C9RA07327B-s3811

RA-009-C9RA07327B-s3812

RA-009-C9RA07327B-s3813

RA-009-C9RA07327B-s3814

RA-009-C9RA07327B-s3815

RA-009-C9RA07327B-s3816

RA-009-C9RA07327B-s3817

RA-009-C9RA07327B-s3818

RA-009-C9RA07327B-s3819

RA-009-C9RA07327B-s3820

RA-009-C9RA07327B-s3821

RA-009-C9RA07327B-s3822

RA-009-C9RA07327B-s3823

RA-009-C9RA07327B-s3824

RA-009-C9RA07327B-s3825

RA-009-C9RA07327B-s3826

RA-009-C9RA07327B-s3827

RA-009-C9RA07327B-s3828

RA-009-C9RA07327B-s3829

RA-009-C9RA07327B-s3830

RA-009-C9RA07327B-s3831

RA-009-C9RA07327B-s3832

RA-009-C9RA07327B-s3833

RA-009-C9RA07327B-s3834

RA-009-C9RA07327B-s3835

RA-009-C9RA07327B-s3836

RA-009-C9RA07327B-s3837

RA-009-C9RA07327B-s3838

RA-009-C9RA07327B-s3839

RA-009-C9RA07327B-s3840

RA-009-C9RA07327B-s3841

RA-009-C9RA07327B-s3842

RA-009-C9RA07327B-s3843

RA-009-C9RA07327B-s3844

RA-009-C9RA07327B-s3845

RA-009-C9RA07327B-s3846

RA-009-C9RA07327B-s3847

RA-009-C9RA07327B-s3848

RA-009-C9RA07327B-s3849

RA-009-C9RA07327B-s3850

RA-009-C9RA07327B-s3851

RA-009-C9RA07327B-s3852

RA-009-C9RA07327B-s3853

RA-009-C9RA07327B-s3854

RA-009-C9RA07327B-s3855

RA-009-C9RA07327B-s3856

RA-009-C9RA07327B-s3857

RA-009-C9RA07327B-s3858

RA-009-C9RA07327B-s3859

RA-009-C9RA07327B-s3860

RA-009-C9RA07327B-s3861

RA-009-C9RA07327B-s3862

RA-009-C9RA07327B-s3863

RA-009-C9RA07327B-s3864

RA-009-C9RA07327B-s3865

RA-009-C9RA07327B-s3866

RA-009-C9RA07327B-s3867

RA-009-C9RA07327B-s3868

RA-009-C9RA07327B-s3869

RA-009-C9RA07327B-s3870

RA-009-C9RA07327B-s3871

RA-009-C9RA07327B-s3872

RA-009-C9RA07327B-s3873

RA-009-C9RA07327B-s3874

RA-009-C9RA07327B-s3875

RA-009-C9RA07327B-s3876

RA-009-C9RA07327B-s3877

RA-009-C9RA07327B-s3878

RA-009-C9RA07327B-s3879

RA-009-C9RA07327B-s3880

RA-009-C9RA07327B-s3881

RA-009-C9RA07327B-s3882

RA-009-C9RA07327B-s3883

RA-009-C9RA07327B-s3884

RA-009-C9RA07327B-s3885

RA-009-C9RA07327B-s3886

RA-009-C9RA07327B-s3887

RA-009-C9RA07327B-s3888

RA-009-C9RA07327B-s3889

RA-009-C9RA07327B-s3890

RA-009-C9RA07327B-s3891

RA-009-C9RA07327B-s3892

RA-009-C9RA07327B-s3893

RA-009-C9RA07327B-s3894

RA-009-C9RA07327B-s3895

RA-009-C9RA07327B-s3896

RA-009-C9RA07327B-s3897

RA-009-C9RA07327B-s3898

RA-009-C9RA07327B-s3899

RA-009-C9RA07327B-s3900

RA-009-C9RA07327B-s3901

RA-009-C9RA07327B-s3902

RA-009-C9RA07327B-s3903

RA-009-C9RA07327B-s3904

RA-009-C9RA07327B-s3905

RA-009-C9RA07327B-s3906

RA-009-C9RA07327B-s3907

RA-009-C9RA07327B-s3908

RA-009-C9RA07327B-s3909

RA-009-C9RA07327B-s3910

RA-009-C9RA07327B-s3911

RA-009-C9RA07327B-s3912

RA-009-C9RA07327B-s3913

RA-009-C9RA07327B-s3914

RA-009-C9RA07327B-s3915

RA-009-C9RA07327B-s3916

RA-009-C9RA07327B-s3917

RA-009-C9RA07327B-s3918

RA-009-C9RA07327B-s3919

RA-009-C9RA07327B-s3920

RA-009-C9RA07327B-s3921

RA-009-C9RA07327B-s3922

RA-009-C9RA07327B-s3923

RA-009-C9RA07327B-s3924

RA-009-C9RA07327B-s3925

RA-009-C9RA07327B-s3926

RA-009-C9RA07327B-s3927

RA-009-C9RA07327B-s3928

RA-009-C9RA07327B-s3929

RA-009-C9RA07327B-s3930

RA-009-C9RA07327B-s3931

RA-009-C9RA07327B-s3932

RA-009-C9RA07327B-s3933

RA-009-C9RA07327B-s3934

RA-009-C9RA07327B-s3935

RA-009-C9RA07327B-s3936

RA-009-C9RA07327B-s3937

RA-009-C9RA07327B-s3938

RA-009-C9RA07327B-s3939

RA-009-C9RA07327B-s3940

RA-009-C9RA07327B-s3941

RA-009-C9RA07327B-s3942

RA-009-C9RA07327B-s3943

RA-009-C9RA07327B-s3944

RA-009-C9RA07327B-s3945

RA-009-C9RA07327B-s3946

RA-009-C9RA07327B-s3947

RA-009-C9RA07327B-s3948

RA-009-C9RA07327B-s3949

RA-009-C9RA07327B-s3950

RA-009-C9RA07327B-s3951

RA-009-C9RA07327B-s3952

RA-009-C9RA07327B-s3953

RA-009-C9RA07327B-s3954

RA-009-C9RA07327B-s3955

RA-009-C9RA07327B-s3956

RA-009-C9RA07327B-s3957

RA-009-C9RA07327B-s3958

RA-009-C9RA07327B-s3959

RA-009-C9RA07327B-s3960

RA-009-C9RA07327B-s3961

RA-009-C9RA07327B-s3962

RA-009-C9RA07327B-s3963

RA-009-C9RA07327B-s3964

RA-009-C9RA07327B-s3965

RA-009-C9RA07327B-s3966

RA-009-C9RA07327B-s3967

RA-009-C9RA07327B-s3968

RA-009-C9RA07327B-s3969

RA-009-C9RA07327B-s3970

RA-009-C9RA07327B-s3971

RA-009-C9RA07327B-s3972

RA-009-C9RA07327B-s3973

RA-009-C9RA07327B-s3974

RA-009-C9RA07327B-s3975

RA-009-C9RA07327B-s3976

RA-009-C9RA07327B-s3977

RA-009-C9RA07327B-s3978

RA-009-C9RA07327B-s3979

RA-009-C9RA07327B-s3980

RA-009-C9RA07327B-s3981

RA-009-C9RA07327B-s3982

RA-009-C9RA07327B-s3983

RA-009-C9RA07327B-s3984

RA-009-C9RA07327B-s3985

RA-009-C9RA07327B-s3986

RA-009-C9RA07327B-s3987

RA-009-C9RA07327B-s3988

RA-009-C9RA07327B-s3989

RA-009-C9RA07327B-s3990

RA-009-C9RA07327B-s3991

RA-009-C9RA07327B-s3992

RA-009-C9RA07327B-s3993

RA-009-C9RA07327B-s3994

RA-009-C9RA07327B-s3995

RA-009-C9RA07327B-s3996

RA-009-C9RA07327B-s3997

RA-009-C9RA07327B-s3998

RA-009-C9RA07327B-s3999

RA-009-C9RA07327B-s4000

RA-009-C9RA07327B-s4001

RA-009-C9RA07327B-s4002

RA-009-C9RA07327B-s4003

RA-009-C9RA07327B-s4004

RA-009-C9RA07327B-s4005

RA-009-C9RA07327B-s4006

RA-009-C9RA07327B-s4007

RA-009-C9RA07327B-s4008

RA-009-C9RA07327B-s4009

RA-009-C9RA07327B-s4010

RA-009-C9RA07327B-s4011

RA-009-C9RA07327B-s4012

RA-009-C9RA07327B-s4013

RA-009-C9RA07327B-s4014

RA-009-C9RA07327B-s4015

RA-009-C9RA07327B-s4016

RA-009-C9RA07327B-s4017

RA-009-C9RA07327B-s4018

RA-009-C9RA07327B-s4019

RA-009-C9RA07327B-s4020

RA-009-C9RA07327B-s4021

RA-009-C9RA07327B-s4022

RA-009-C9RA07327B-s4023

RA-009-C9RA07327B-s4024

RA-009-C9RA07327B-s4025

RA-009-C9RA07327B-s4026

RA-009-C9RA07327B-s4027

RA-009-C9RA07327B-s4028

RA-009-C9RA07327B-s4029

RA-009-C9RA07327B-s4030

RA-009-C9RA07327B-s4031

RA-009-C9RA07327B-s4032

RA-009-C9RA07327B-s4033

RA-009-C9RA07327B-s4034

RA-009-C9RA07327B-s4035

RA-009-C9RA07327B-s4036

RA-009-C9RA07327B-s4037

RA-009-C9RA07327B-s4038

RA-009-C9RA07327B-s4039

RA-009-C9RA07327B-s4040

RA-009-C9RA07327B-s4041

RA-009-C9RA07327B-s4042

RA-009-C9RA07327B-s4043

RA-009-C9RA07327B-s4044

RA-009-C9RA07327B-s4045

RA-009-C9RA07327B-s4046

RA-009-C9RA07327B-s4047

RA-009-C9RA07327B-s4048

RA-009-C9RA07327B-s4049

RA-009-C9RA07327B-s4050

RA-009-C9RA07327B-s4051

RA-009-C9RA07327B-s4052

RA-009-C9RA07327B-s4053

RA-009-C9RA07327B-s4054

RA-009-C9RA07327B-s4055

RA-009-C9RA07327B-s4056

RA-009-C9RA07327B-s4057

RA-009-C9RA07327B-s4058

RA-009-C9RA07327B-s4059

RA-009-C9RA07327B-s4060

RA-009-C9RA07327B-s4061

RA-009-C9RA07327B-s4062

RA-009-C9RA07327B-s4063

RA-009-C9RA07327B-s4064

RA-009-C9RA07327B-s4065

RA-009-C9RA07327B-s4066

RA-009-C9RA07327B-s4067

RA-009-C9RA07327B-s4068

RA-009-C9RA07327B-s4069

RA-009-C9RA07327B-s4070

RA-009-C9RA07327B-s4071

RA-009-C9RA07327B-s4072

RA-009-C9RA07327B-s4073

RA-009-C9RA07327B-s4074

RA-009-C9RA07327B-s4075

RA-009-C9RA07327B-s4076

RA-009-C9RA07327B-s4077

RA-009-C9RA07327B-s4078

RA-009-C9RA07327B-s4079

RA-009-C9RA07327B-s4080

RA-009-C9RA07327B-s4081

RA-009-C9RA07327B-s4082

RA-009-C9RA07327B-s4083

RA-009-C9RA07327B-s4084

RA-009-C9RA07327B-s4085

RA-009-C9RA07327B-s4086

RA-009-C9RA07327B-s4087

RA-009-C9RA07327B-s4088

RA-009-C9RA07327B-s4089

RA-009-C9RA07327B-s4090

RA-009-C9RA07327B-s4091

RA-009-C9RA07327B-s4092

RA-009-C9RA07327B-s4093

RA-009-C9RA07327B-s4094

RA-009-C9RA07327B-s4095

RA-009-C9RA07327B-s4096

RA-009-C9RA07327B-s4097

RA-009-C9RA07327B-s4098

RA-009-C9RA07327B-s4099

RA-009-C9RA07327B-s4100

RA-009-C9RA07327B-s4101

RA-009-C9RA07327B-s4102

RA-009-C9RA07327B-s4103

RA-009-C9RA07327B-s4104

RA-009-C9RA07327B-s4105

RA-009-C9RA07327B-s4106

RA-009-C9RA07327B-s4107

RA-009-C9RA07327B-s4108

RA-009-C9RA07327B-s4109

RA-009-C9RA07327B-s4110

RA-009-C9RA07327B-s4111

RA-009-C9RA07327B-s4112

RA-009-C9RA07327B-s4113

RA-009-C9RA07327B-s4114

RA-009-C9RA07327B-s4115

RA-009-C9RA07327B-s4116

RA-009-C9RA07327B-s4117

RA-009-C9RA07327B-s4118

RA-009-C9RA07327B-s4119

RA-009-C9RA07327B-s4120

RA-009-C9RA07327B-s4121

RA-009-C9RA07327B-s4122

RA-009-C9RA07327B-s4123

RA-009-C9RA07327B-s4124

RA-009-C9RA07327B-s4125

RA-009-C9RA07327B-s4126

RA-009-C9RA07327B-s4127

RA-009-C9RA07327B-s4128

RA-009-C9RA07327B-s4129

RA-009-C9RA07327B-s4130

RA-009-C9RA07327B-s4131

RA-009-C9RA07327B-s4132

RA-009-C9RA07327B-s4133

RA-009-C9RA07327B-s4134

RA-009-C9RA07327B-s4135

RA-009-C9RA07327B-s4136

RA-009-C9RA07327B-s4137

RA-009-C9RA07327B-s4138

RA-009-C9RA07327B-s4139

RA-009-C9RA07327B-s4140

RA-009-C9RA07327B-s4141

RA-009-C9RA07327B-s4142

RA-009-C9RA07327B-s4143

RA-009-C9RA07327B-s4144

RA-009-C9RA07327B-s4145

RA-009-C9RA07327B-s4146

RA-009-C9RA07327B-s4147

RA-009-C9RA07327B-s4148

RA-009-C9RA07327B-s4149

RA-009-C9RA07327B-s4150

RA-009-C9RA07327B-s4151

RA-009-C9RA07327B-s4152

RA-009-C9RA07327B-s4153

RA-009-C9RA07327B-s4154

RA-009-C9RA07327B-s4155

RA-009-C9RA07327B-s4156

RA-009-C9RA07327B-s4157

RA-009-C9RA07327B-s4158

RA-009-C9RA07327B-s4159

RA-009-C9RA07327B-s4160

RA-009-C9RA07327B-s4161

RA-009-C9RA07327B-s4162

RA-009-C9RA07327B-s4163

RA-009-C9RA07327B-s4164

RA-009-C9RA07327B-s4165

RA-009-C9RA07327B-s4166

RA-009-C9RA07327B-s4167

RA-009-C9RA07327B-s4168

RA-009-C9RA07327B-s4169

RA-009-C9RA07327B-s4170

RA-009-C9RA07327B-s4171

RA-009-C9RA07327B-s4172

RA-009-C9RA07327B-s4173

RA-009-C9RA07327B-s4174

RA-009-C9RA07327B-s4175

RA-009-C9RA07327B-s4176

RA-009-C9RA07327B-s4177

RA-009-C9RA07327B-s4178

RA-009-C9RA07327B-s4179

RA-009-C9RA07327B-s4180

RA-009-C9RA07327B-s4181

RA-009-C9RA07327B-s4182

RA-009-C9RA07327B-s4183

RA-009-C9RA07327B-s4184

RA-009-C9RA07327B-s4185

RA-009-C9RA07327B-s4186

RA-009-C9RA07327B-s4187

RA-009-C9RA07327B-s4188

RA-009-C9RA07327B-s4189

RA-009-C9RA07327B-s4190

RA-009-C9RA07327B-s4191

RA-009-C9RA07327B-s4192

RA-009-C9RA07327B-s4193

RA-009-C9RA07327B-s4194

RA-009-C9RA07327B-s4195

RA-009-C9RA07327B-s4196

RA-009-C9RA07327B-s4197

RA-009-C9RA07327B-s4198

RA-009-C9RA07327B-s4199

RA-009-C9RA07327B-s4200

RA-009-C9RA07327B-s4201

RA-009-C9RA07327B-s4202

RA-009-C9RA07327B-s4203

RA-009-C9RA07327B-s4204

RA-009-C9RA07327B-s4205

RA-009-C9RA07327B-s4206

RA-009-C9RA07327B-s4207

RA-009-C9RA07327B-s4208

RA-009-C9RA07327B-s4209

RA-009-C9RA07327B-s4210

RA-009-C9RA07327B-s4211

RA-009-C9RA07327B-s4212

RA-009-C9RA07327B-s4213

RA-009-C9RA07327B-s4214

RA-009-C9RA07327B-s4215

RA-009-C9RA07327B-s4216

RA-009-C9RA07327B-s4217

RA-009-C9RA07327B-s4218

RA-009-C9RA07327B-s4219

RA-009-C9RA07327B-s4220

RA-009-C9RA07327B-s4221

RA-009-C9RA07327B-s4222

RA-009-C9RA07327B-s4223

RA-009-C9RA07327B-s4224

RA-009-C9RA07327B-s4225

RA-009-C9RA07327B-s4226

RA-009-C9RA07327B-s4227

RA-009-C9RA07327B-s4228

RA-009-C9RA07327B-s4229

RA-009-C9RA07327B-s4230

RA-009-C9RA07327B-s4231

RA-009-C9RA07327B-s4232

RA-009-C9RA07327B-s4233

RA-009-C9RA07327B-s4234

RA-009-C9RA07327B-s4235

RA-009-C9RA07327B-s4236

RA-009-C9RA07327B-s4237

RA-009-C9RA07327B-s4238

RA-009-C9RA07327B-s4239

RA-009-C9RA07327B-s4240

RA-009-C9RA07327B-s4241

RA-009-C9RA07327B-s4242

RA-009-C9RA07327B-s4243

RA-009-C9RA07327B-s4244

RA-009-C9RA07327B-s4245

RA-009-C9RA07327B-s4246

RA-009-C9RA07327B-s4247

RA-009-C9RA07327B-s4248

RA-009-C9RA07327B-s4249

RA-009-C9RA07327B-s4250

RA-009-C9RA07327B-s4251

RA-009-C9RA07327B-s4252

RA-009-C9RA07327B-s4253

RA-009-C9RA07327B-s4254

RA-009-C9RA07327B-s4255

RA-009-C9RA07327B-s4256

RA-009-C9RA07327B-s4257

RA-009-C9RA07327B-s4258

RA-009-C9RA07327B-s4259

RA-009-C9RA07327B-s4260

RA-009-C9RA07327B-s4261

RA-009-C9RA07327B-s4262

RA-009-C9RA07327B-s4263

RA-009-C9RA07327B-s4264

RA-009-C9RA07327B-s4265

RA-009-C9RA07327B-s4266

RA-009-C9RA07327B-s4267

RA-009-C9RA07327B-s4268

RA-009-C9RA07327B-s4269

RA-009-C9RA07327B-s4270

RA-009-C9RA07327B-s4271

RA-009-C9RA07327B-s4272

RA-009-C9RA07327B-s4273

RA-009-C9RA07327B-s4274

RA-009-C9RA07327B-s4275

RA-009-C9RA07327B-s4276

RA-009-C9RA07327B-s4277

RA-009-C9RA07327B-s4278

RA-009-C9RA07327B-s4279

RA-009-C9RA07327B-s4280

RA-009-C9RA07327B-s4281

RA-009-C9RA07327B-s4282

RA-009-C9RA07327B-s4283

RA-009-C9RA07327B-s4284

RA-009-C9RA07327B-s4285

RA-009-C9RA07327B-s4286

RA-009-C9RA07327B-s4287

RA-009-C9RA07327B-s4288

RA-009-C9RA07327B-s4289

RA-009-C9RA07327B-s4290

RA-009-C9RA07327B-s4291

RA-009-C9RA07327B-s4292

RA-009-C9RA07327B-s4293

RA-009-C9RA07327B-s4294

RA-009-C9RA07327B-s4295

RA-009-C9RA07327B-s4296

RA-009-C9RA07327B-s4297

RA-009-C9RA07327B-s4298

RA-009-C9RA07327B-s4299

RA-009-C9RA07327B-s4300

RA-009-C9RA07327B-s4301

RA-009-C9RA07327B-s4302

RA-009-C9RA07327B-s4303

RA-009-C9RA07327B-s4304

RA-009-C9RA07327B-s4305

RA-009-C9RA07327B-s4306

RA-009-C9RA07327B-s4307

RA-009-C9RA07327B-s4308

RA-009-C9RA07327B-s4309

RA-009-C9RA07327B-s4310

RA-009-C9RA07327B-s4311

RA-009-C9RA07327B-s4312

RA-009-C9RA07327B-s4313

RA-009-C9RA07327B-s4314

RA-009-C9RA07327B-s4315

RA-009-C9RA07327B-s4316

RA-009-C9RA07327B-s4317

RA-009-C9RA07327B-s4318

RA-009-C9RA07327B-s4319

RA-009-C9RA07327B-s4320

RA-009-C9RA07327B-s4321

RA-009-C9RA07327B-s4322

RA-009-C9RA07327B-s4323

RA-009-C9RA07327B-s4324

RA-009-C9RA07327B-s4325

RA-009-C9RA07327B-s4326

RA-009-C9RA07327B-s4327

RA-009-C9RA07327B-s4328

RA-009-C9RA07327B-s4329

RA-009-C9RA07327B-s4330

RA-009-C9RA07327B-s4331

RA-009-C9RA07327B-s4332

RA-009-C9RA07327B-s4333

RA-009-C9RA07327B-s4334

RA-009-C9RA07327B-s4335

RA-009-C9RA07327B-s4336

RA-009-C9RA07327B-s4337

RA-009-C9RA07327B-s4338

RA-009-C9RA07327B-s4339

RA-009-C9RA07327B-s4340

RA-009-C9RA07327B-s4341

RA-009-C9RA07327B-s4342

RA-009-C9RA07327B-s4343

RA-009-C9RA07327B-s4344

RA-009-C9RA07327B-s4345

RA-009-C9RA07327B-s4346

RA-009-C9RA07327B-s4347

RA-009-C9RA07327B-s4348

RA-009-C9RA07327B-s4349

RA-009-C9RA07327B-s4350

RA-009-C9RA07327B-s4351

RA-009-C9RA07327B-s4352

RA-009-C9RA07327B-s4353

RA-009-C9RA07327B-s4354

RA-009-C9RA07327B-s4355

RA-009-C9RA07327B-s4356

RA-009-C9RA07327B-s4357

RA-009-C9RA07327B-s4358

RA-009-C9RA07327B-s4359

RA-009-C9RA07327B-s4360

RA-009-C9RA07327B-s4361

RA-009-C9RA07327B-s4362

RA-009-C9RA07327B-s4363

RA-009-C9RA07327B-s4364

RA-009-C9RA07327B-s4365

RA-009-C9RA07327B-s4366

RA-009-C9RA07327B-s4367

RA-009-C9RA07327B-s4368

RA-009-C9RA07327B-s4369

RA-009-C9RA07327B-s4370

RA-009-C9RA07327B-s4371

RA-009-C9RA07327B-s4372

RA-009-C9RA07327B-s4373

RA-009-C9RA07327B-s4374

RA-009-C9RA07327B-s4375

RA-009-C9RA07327B-s4376

RA-009-C9RA07327B-s4377

RA-009-C9RA07327B-s4378

RA-009-C9RA07327B-s4379

RA-009-C9RA07327B-s4380

RA-009-C9RA07327B-s4381

RA-009-C9RA07327B-s4382

RA-009-C9RA07327B-s4383

RA-009-C9RA07327B-s4384

RA-009-C9RA07327B-s4385

RA-009-C9RA07327B-s4386

RA-009-C9RA07327B-s4387

RA-009-C9RA07327B-s4388

RA-009-C9RA07327B-s4389

RA-009-C9RA07327B-s4390

RA-009-C9RA07327B-s4391

RA-009-C9RA07327B-s4392

RA-009-C9RA07327B-s4393

RA-009-C9RA07327B-s4394

RA-009-C9RA07327B-s4395

RA-009-C9RA07327B-s4396

RA-009-C9RA07327B-s4397

RA-009-C9RA07327B-s4398

RA-009-C9RA07327B-s4399

RA-009-C9RA07327B-s4400

RA-009-C9RA07327B-s4401

RA-009-C9RA07327B-s4402

RA-009-C9RA07327B-s4403

RA-009-C9RA07327B-s4404

RA-009-C9RA07327B-s4405

RA-009-C9RA07327B-s4406

RA-009-C9RA07327B-s4407

RA-009-C9RA07327B-s4408

RA-009-C9RA07327B-s4409

RA-009-C9RA07327B-s4410

RA-009-C9RA07327B-s4411

RA-009-C9RA07327B-s4412

RA-009-C9RA07327B-s4413

RA-009-C9RA07327B-s4414

RA-009-C9RA07327B-s4415

RA-009-C9RA07327B-s4416

RA-009-C9RA07327B-s4417

RA-009-C9RA07327B-s4418

RA-009-C9RA07327B-s4419

RA-009-C9RA07327B-s4420

RA-009-C9RA07327B-s4421

RA-009-C9RA07327B-s4422

RA-009-C9RA07327B-s4423

RA-009-C9RA07327B-s4424

RA-009-C9RA07327B-s4425

RA-009-C9RA07327B-s4426

RA-009-C9RA07327B-s4427

RA-009-C9RA07327B-s4428

RA-009-C9RA07327B-s4429

RA-009-C9RA07327B-s4430

RA-009-C9RA07327B-s4431

RA-009-C9RA07327B-s4432

RA-009-C9RA07327B-s4433

RA-009-C9RA07327B-s4434

RA-009-C9RA07327B-s4435

RA-009-C9RA07327B-s4436

RA-009-C9RA07327B-s4437

RA-009-C9RA07327B-s4438

RA-009-C9RA07327B-s4439

RA-009-C9RA07327B-s4440

RA-009-C9RA07327B-s4441

RA-009-C9RA07327B-s4442

RA-009-C9RA07327B-s4443

RA-009-C9RA07327B-s4444

RA-009-C9RA07327B-s4445

RA-009-C9RA07327B-s4446

RA-009-C9RA07327B-s4447

RA-009-C9RA07327B-s4448

RA-009-C9RA07327B-s4449

RA-009-C9RA07327B-s4450

RA-009-C9RA07327B-s4451

RA-009-C9RA07327B-s4452

RA-009-C9RA07327B-s4453

RA-009-C9RA07327B-s4454

RA-009-C9RA07327B-s4455

RA-009-C9RA07327B-s4456

RA-009-C9RA07327B-s4457

RA-009-C9RA07327B-s4458

RA-009-C9RA07327B-s4459

RA-009-C9RA07327B-s4460

RA-009-C9RA07327B-s4461

RA-009-C9RA07327B-s4462

RA-009-C9RA07327B-s4463

RA-009-C9RA07327B-s4464

RA-009-C9RA07327B-s4465

RA-009-C9RA07327B-s4466

RA-009-C9RA07327B-s4467

RA-009-C9RA07327B-s4468

RA-009-C9RA07327B-s4469

RA-009-C9RA07327B-s4470

RA-009-C9RA07327B-s4471

RA-009-C9RA07327B-s4472

RA-009-C9RA07327B-s4473

RA-009-C9RA07327B-s4474

RA-009-C9RA07327B-s4475

RA-009-C9RA07327B-s4476

RA-009-C9RA07327B-s4477

RA-009-C9RA07327B-s4478

RA-009-C9RA07327B-s4479

RA-009-C9RA07327B-s4480

RA-009-C9RA07327B-s4481

RA-009-C9RA07327B-s4482

RA-009-C9RA07327B-s4483

RA-009-C9RA07327B-s4484

RA-009-C9RA07327B-s4485

RA-009-C9RA07327B-s4486

RA-009-C9RA07327B-s4487

RA-009-C9RA07327B-s4488

RA-009-C9RA07327B-s4489

RA-009-C9RA07327B-s4490

RA-009-C9RA07327B-s4491

RA-009-C9RA07327B-s4492

RA-009-C9RA07327B-s4493

RA-009-C9RA07327B-s4494

RA-009-C9RA07327B-s4495

RA-009-C9RA07327B-s4496

RA-009-C9RA07327B-s4497

RA-009-C9RA07327B-s4498

RA-009-C9RA07327B-s4499

RA-009-C9RA07327B-s4500

RA-009-C9RA07327B-s4501

RA-009-C9RA07327B-s4502

RA-009-C9RA07327B-s4503

RA-009-C9RA07327B-s4504

RA-009-C9RA07327B-s4505

RA-009-C9RA07327B-s4506

RA-009-C9RA07327B-s4507

RA-009-C9RA07327B-s4508

RA-009-C9RA07327B-s4509

RA-009-C9RA07327B-s4510

RA-009-C9RA07327B-s4511

RA-009-C9RA07327B-s4512

RA-009-C9RA07327B-s4513

RA-009-C9RA07327B-s4514

RA-009-C9RA07327B-s4515

RA-009-C9RA07327B-s4516

RA-009-C9RA07327B-s4517

RA-009-C9RA07327B-s4518

RA-009-C9RA07327B-s4519

RA-009-C9RA07327B-s4520

RA-009-C9RA07327B-s4521

RA-009-C9RA07327B-s4522

RA-009-C9RA07327B-s4523

RA-009-C9RA07327B-s4524

RA-009-C9RA07327B-s4525

RA-009-C9RA07327B-s4526

RA-009-C9RA07327B-s4527

RA-009-C9RA07327B-s4528

RA-009-C9RA07327B-s4529

RA-009-C9RA07327B-s4530

RA-009-C9RA07327B-s4531

RA-009-C9RA07327B-s4532

RA-009-C9RA07327B-s4533

RA-009-C9RA07327B-s4534

RA-009-C9RA07327B-s4535

RA-009-C9RA07327B-s4536

RA-009-C9RA07327B-s4537

RA-009-C9RA07327B-s4538

RA-009-C9RA07327B-s4539

RA-009-C9RA07327B-s4540

RA-009-C9RA07327B-s4541

RA-009-C9RA07327B-s4542

RA-009-C9RA07327B-s4543

RA-009-C9RA07327B-s4544

RA-009-C9RA07327B-s4545

RA-009-C9RA07327B-s4546

RA-009-C9RA07327B-s4547

RA-009-C9RA07327B-s4548

RA-009-C9RA07327B-s4549

RA-009-C9RA07327B-s4550

RA-009-C9RA07327B-s4551

RA-009-C9RA07327B-s4552

RA-009-C9RA07327B-s4553

RA-009-C9RA07327B-s4554

RA-009-C9RA07327B-s4555

RA-009-C9RA07327B-s4556

RA-009-C9RA07327B-s4557

RA-009-C9RA07327B-s4558

RA-009-C9RA07327B-s4559

RA-009-C9RA07327B-s4560

RA-009-C9RA07327B-s4561

RA-009-C9RA07327B-s4562

RA-009-C9RA07327B-s4563

RA-009-C9RA07327B-s4564

RA-009-C9RA07327B-s4565

RA-009-C9RA07327B-s4566

RA-009-C9RA07327B-s4567

RA-009-C9RA07327B-s4568

RA-009-C9RA07327B-s4569

RA-009-C9RA07327B-s4570

RA-009-C9RA07327B-s4571

RA-009-C9RA07327B-s4572

RA-009-C9RA07327B-s4573

RA-009-C9RA07327B-s4574

RA-009-C9RA07327B-s4575

RA-009-C9RA07327B-s4576

RA-009-C9RA07327B-s4577

RA-009-C9RA07327B-s4578

RA-009-C9RA07327B-s4579

RA-009-C9RA07327B-s4580

RA-009-C9RA07327B-s4581

RA-009-C9RA07327B-s4582

RA-009-C9RA07327B-s4583

RA-009-C9RA07327B-s4584

RA-009-C9RA07327B-s4585

RA-009-C9RA07327B-s4586

RA-009-C9RA07327B-s4587

RA-009-C9RA07327B-s4588

RA-009-C9RA07327B-s4589

RA-009-C9RA07327B-s4590

RA-009-C9RA07327B-s4591

RA-009-C9RA07327B-s4592

RA-009-C9RA07327B-s4593

RA-009-C9RA07327B-s4594

RA-009-C9RA07327B-s4595

RA-009-C9RA07327B-s4596

RA-009-C9RA07327B-s4597

RA-009-C9RA07327B-s4598

RA-009-C9RA07327B-s4599

RA-009-C9RA07327B-s4600

RA-009-C9RA07327B-s4601

RA-009-C9RA07327B-s4602

RA-009-C9RA07327B-s4603

RA-009-C9RA07327B-s4604

RA-009-C9RA07327B-s4605

RA-009-C9RA07327B-s4606

RA-009-C9RA07327B-s4607

RA-009-C9RA07327B-s4608

RA-009-C9RA07327B-s4609

RA-009-C9RA07327B-s4610

RA-009-C9RA07327B-s4611

RA-009-C9RA07327B-s4612

RA-009-C9RA07327B-s4613

RA-009-C9RA07327B-s4614

RA-009-C9RA07327B-s4615

RA-009-C9RA07327B-s4616

RA-009-C9RA07327B-s4617

RA-009-C9RA07327B-s4618

RA-009-C9RA07327B-s4619

RA-009-C9RA07327B-s4620

RA-009-C9RA07327B-s4621

RA-009-C9RA07327B-s4622

RA-009-C9RA07327B-s4623

RA-009-C9RA07327B-s4624

RA-009-C9RA07327B-s4625

RA-009-C9RA07327B-s4626

RA-009-C9RA07327B-s4627

RA-009-C9RA07327B-s4628

RA-009-C9RA07327B-s4629

RA-009-C9RA07327B-s4630

RA-009-C9RA07327B-s4631

RA-009-C9RA07327B-s4632

RA-009-C9RA07327B-s4633

RA-009-C9RA07327B-s4634

RA-009-C9RA07327B-s4635

RA-009-C9RA07327B-s4636

RA-009-C9RA07327B-s4637

RA-009-C9RA07327B-s4638

RA-009-C9RA07327B-s4639

RA-009-C9RA07327B-s4640

RA-009-C9RA07327B-s4641

RA-009-C9RA07327B-s4642

RA-009-C9RA07327B-s4643

RA-009-C9RA07327B-s4644

RA-009-C9RA07327B-s4645

RA-009-C9RA07327B-s4646

RA-009-C9RA07327B-s4647

RA-009-C9RA07327B-s4648

RA-009-C9RA07327B-s4649

RA-009-C9RA07327B-s4650

RA-009-C9RA07327B-s4651

RA-009-C9RA07327B-s4652

RA-009-C9RA07327B-s4653

RA-009-C9RA07327B-s4654

RA-009-C9RA07327B-s4655

RA-009-C9RA07327B-s4656

RA-009-C9RA07327B-s4657

RA-009-C9RA07327B-s4658

RA-009-C9RA07327B-s4659

RA-009-C9RA07327B-s4660

RA-009-C9RA07327B-s4661

RA-009-C9RA07327B-s4662

RA-009-C9RA07327B-s4663

RA-009-C9RA07327B-s4664

RA-009-C9RA07327B-s4665

RA-009-C9RA07327B-s4666

RA-009-C9RA07327B-s4667

RA-009-C9RA07327B-s4668

RA-009-C9RA07327B-s4669

RA-009-C9RA07327B-s4670

RA-009-C9RA07327B-s4671

RA-009-C9RA07327B-s4672

RA-009-C9RA07327B-s4673

RA-009-C9RA07327B-s4674

RA-009-C9RA07327B-s4675

RA-009-C9RA07327B-s4676

RA-009-C9RA07327B-s4677

RA-009-C9RA07327B-s4678

RA-009-C9RA07327B-s4679

RA-009-C9RA07327B-s4680

RA-009-C9RA07327B-s4681

RA-009-C9RA07327B-s4682

RA-009-C9RA07327B-s4683

RA-009-C9RA07327B-s4684

RA-009-C9RA07327B-s4685

RA-009-C9RA07327B-s4686

RA-009-C9RA07327B-s4687

RA-009-C9RA07327B-s4688

RA-009-C9RA07327B-s4689

RA-009-C9RA07327B-s4690

RA-009-C9RA07327B-s4691

RA-009-C9RA07327B-s4692

RA-009-C9RA07327B-s4693

RA-009-C9RA07327B-s4694

RA-009-C9RA07327B-s4695

RA-009-C9RA07327B-s4696

RA-009-C9RA07327B-s4697

RA-009-C9RA07327B-s4698

RA-009-C9RA07327B-s4699

RA-009-C9RA07327B-s4700

RA-009-C9RA07327B-s4701

RA-009-C9RA07327B-s4702

RA-009-C9RA07327B-s4703

RA-009-C9RA07327B-s4704

RA-009-C9RA07327B-s4705

RA-009-C9RA07327B-s4706

RA-009-C9RA07327B-s4707

RA-009-C9RA07327B-s4708

RA-009-C9RA07327B-s4709

RA-009-C9RA07327B-s4710

RA-009-C9RA07327B-s4711

RA-009-C9RA07327B-s4712

RA-009-C9RA07327B-s4713

RA-009-C9RA07327B-s4714

RA-009-C9RA07327B-s4715

RA-009-C9RA07327B-s4716

RA-009-C9RA07327B-s4717

RA-009-C9RA07327B-s4718

RA-009-C9RA07327B-s4719

RA-009-C9RA07327B-s4720

RA-009-C9RA07327B-s4721

RA-009-C9RA07327B-s4722

RA-009-C9RA07327B-s4723

RA-009-C9RA07327B-s4724

RA-009-C9RA07327B-s4725

RA-009-C9RA07327B-s4726

RA-009-C9RA07327B-s4727

RA-009-C9RA07327B-s4728

RA-009-C9RA07327B-s4729

RA-009-C9RA07327B-s4730

RA-009-C9RA07327B-s4731

RA-009-C9RA07327B-s4732

RA-009-C9RA07327B-s4733

RA-009-C9RA07327B-s4734

RA-009-C9RA07327B-s4735

RA-009-C9RA07327B-s4736

RA-009-C9RA07327B-s4737

RA-009-C9RA07327B-s4738

RA-009-C9RA07327B-s4739

RA-009-C9RA07327B-s4740

RA-009-C9RA07327B-s4741

RA-009-C9RA07327B-s4742

RA-009-C9RA07327B-s4743

RA-009-C9RA07327B-s4744

RA-009-C9RA07327B-s4745

RA-009-C9RA07327B-s4746

RA-009-C9RA07327B-s4747

RA-009-C9RA07327B-s4748

RA-009-C9RA07327B-s4749

RA-009-C9RA07327B-s4750

RA-009-C9RA07327B-s4751

RA-009-C9RA07327B-s4752

RA-009-C9RA07327B-s4753

RA-009-C9RA07327B-s4754

RA-009-C9RA07327B-s4755

RA-009-C9RA07327B-s4756

RA-009-C9RA07327B-s4757

RA-009-C9RA07327B-s4758

RA-009-C9RA07327B-s4759

RA-009-C9RA07327B-s4760

RA-009-C9RA07327B-s4761

RA-009-C9RA07327B-s4762

RA-009-C9RA07327B-s4763

RA-009-C9RA07327B-s4764

RA-009-C9RA07327B-s4765

RA-009-C9RA07327B-s4766

RA-009-C9RA07327B-s4767

RA-009-C9RA07327B-s4768

RA-009-C9RA07327B-s4769

RA-009-C9RA07327B-s4770

RA-009-C9RA07327B-s4771

RA-009-C9RA07327B-s4772

RA-009-C9RA07327B-s4773

RA-009-C9RA07327B-s4774

RA-009-C9RA07327B-s4775

RA-009-C9RA07327B-s4776

RA-009-C9RA07327B-s4777

RA-009-C9RA07327B-s4778

RA-009-C9RA07327B-s4779

RA-009-C9RA07327B-s4780

RA-009-C9RA07327B-s4781

RA-009-C9RA07327B-s4782

RA-009-C9RA07327B-s4783

RA-009-C9RA07327B-s4784

RA-009-C9RA07327B-s4785

RA-009-C9RA07327B-s4786

RA-009-C9RA07327B-s4787

RA-009-C9RA07327B-s4788

RA-009-C9RA07327B-s4789

RA-009-C9RA07327B-s4790

RA-009-C9RA07327B-s4791

RA-009-C9RA07327B-s4792

RA-009-C9RA07327B-s4793

RA-009-C9RA07327B-s4794

RA-009-C9RA07327B-s4795

RA-009-C9RA07327B-s4796

RA-009-C9RA07327B-s4797

RA-009-C9RA07327B-s4798

RA-009-C9RA07327B-s4799

RA-009-C9RA07327B-s4800

RA-009-C9RA07327B-s4801

RA-009-C9RA07327B-s4802

RA-009-C9RA07327B-s4803

RA-009-C9RA07327B-s4804

RA-009-C9RA07327B-s4805

RA-009-C9RA07327B-s4806

RA-009-C9RA07327B-s4807

RA-009-C9RA07327B-s4808

RA-009-C9RA07327B-s4809

RA-009-C9RA07327B-s4810

RA-009-C9RA07327B-s4811

RA-009-C9RA07327B-s4812

RA-009-C9RA07327B-s4813

RA-009-C9RA07327B-s4814

RA-009-C9RA07327B-s4815

RA-009-C9RA07327B-s4816

RA-009-C9RA07327B-s4817

RA-009-C9RA07327B-s4818

RA-009-C9RA07327B-s4819

RA-009-C9RA07327B-s4820

RA-009-C9RA07327B-s4821

RA-009-C9RA07327B-s4822

RA-009-C9RA07327B-s4823

RA-009-C9RA07327B-s4824

RA-009-C9RA07327B-s4825

RA-009-C9RA07327B-s4826

RA-009-C9RA07327B-s4827

RA-009-C9RA07327B-s4828

RA-009-C9RA07327B-s4829

RA-009-C9RA07327B-s4830

RA-009-C9RA07327B-s4831

RA-009-C9RA07327B-s4832

RA-009-C9RA07327B-s4833

RA-009-C9RA07327B-s4834

RA-009-C9RA07327B-s4835

RA-009-C9RA07327B-s4836

RA-009-C9RA07327B-s4837

RA-009-C9RA07327B-s4838

RA-009-C9RA07327B-s4839

RA-009-C9RA07327B-s4840

RA-009-C9RA07327B-s4841

RA-009-C9RA07327B-s4842

RA-009-C9RA07327B-s4843

RA-009-C9RA07327B-s4844

RA-009-C9RA07327B-s4845

RA-009-C9RA07327B-s4846

RA-009-C9RA07327B-s4847

RA-009-C9RA07327B-s4848

RA-009-C9RA07327B-s4849

RA-009-C9RA07327B-s4850

RA-009-C9RA07327B-s4851

RA-009-C9RA07327B-s4852

RA-009-C9RA07327B-s4853

RA-009-C9RA07327B-s4854

RA-009-C9RA07327B-s4855

RA-009-C9RA07327B-s4856

RA-009-C9RA07327B-s4857

RA-009-C9RA07327B-s4858

RA-009-C9RA07327B-s4859

RA-009-C9RA07327B-s4860

RA-009-C9RA07327B-s4861

RA-009-C9RA07327B-s4862

RA-009-C9RA07327B-s4863

RA-009-C9RA07327B-s4864

RA-009-C9RA07327B-s4865

RA-009-C9RA07327B-s4866

RA-009-C9RA07327B-s4867

RA-009-C9RA07327B-s4868

RA-009-C9RA07327B-s4869

RA-009-C9RA07327B-s4870

RA-009-C9RA07327B-s4871

RA-009-C9RA07327B-s4872

RA-009-C9RA07327B-s4873

RA-009-C9RA07327B-s4874

RA-009-C9RA07327B-s4875

RA-009-C9RA07327B-s4876

RA-009-C9RA07327B-s4877

RA-009-C9RA07327B-s4878

RA-009-C9RA07327B-s4879

RA-009-C9RA07327B-s4880

RA-009-C9RA07327B-s4881

RA-009-C9RA07327B-s4882

RA-009-C9RA07327B-s4883

RA-009-C9RA07327B-s4884

RA-009-C9RA07327B-s4885

RA-009-C9RA07327B-s4886

RA-009-C9RA07327B-s4887

RA-009-C9RA07327B-s4888

RA-009-C9RA07327B-s4889

RA-009-C9RA07327B-s4890

RA-009-C9RA07327B-s4891

RA-009-C9RA07327B-s4892

RA-009-C9RA07327B-s4893

RA-009-C9RA07327B-s4894

RA-009-C9RA07327B-s4895

RA-009-C9RA07327B-s4896

RA-009-C9RA07327B-s4897

RA-009-C9RA07327B-s4898

RA-009-C9RA07327B-s4899

RA-009-C9RA07327B-s4900

RA-009-C9RA07327B-s4901

RA-009-C9RA07327B-s4902

RA-009-C9RA07327B-s4903

RA-009-C9RA07327B-s4904

RA-009-C9RA07327B-s4905

RA-009-C9RA07327B-s4906

RA-009-C9RA07327B-s4907

RA-009-C9RA07327B-s4908

RA-009-C9RA07327B-s4909

RA-009-C9RA07327B-s4910

RA-009-C9RA07327B-s4911

RA-009-C9RA07327B-s4912

RA-009-C9RA07327B-s4913

RA-009-C9RA07327B-s4914

RA-009-C9RA07327B-s4915

RA-009-C9RA07327B-s4916

RA-009-C9RA07327B-s4917

RA-009-C9RA07327B-s4918

RA-009-C9RA07327B-s4919

RA-009-C9RA07327B-s4920

RA-009-C9RA07327B-s4921

RA-009-C9RA07327B-s4922

RA-009-C9RA07327B-s4923

RA-009-C9RA07327B-s4924

RA-009-C9RA07327B-s4925

RA-009-C9RA07327B-s4926

RA-009-C9RA07327B-s4927

RA-009-C9RA07327B-s4928

RA-009-C9RA07327B-s4929

RA-009-C9RA07327B-s4930

RA-009-C9RA07327B-s4931

RA-009-C9RA07327B-s4932

RA-009-C9RA07327B-s4933

RA-009-C9RA07327B-s4934

RA-009-C9RA07327B-s4935

RA-009-C9RA07327B-s4936

RA-009-C9RA07327B-s4937

RA-009-C9RA07327B-s4938

RA-009-C9RA07327B-s4939

RA-009-C9RA07327B-s4940

RA-009-C9RA07327B-s4941

RA-009-C9RA07327B-s4942

RA-009-C9RA07327B-s4943

RA-009-C9RA07327B-s4944

RA-009-C9RA07327B-s4945

RA-009-C9RA07327B-s4946

RA-009-C9RA07327B-s4947

RA-009-C9RA07327B-s4948

RA-009-C9RA07327B-s4949

RA-009-C9RA07327B-s4950

RA-009-C9RA07327B-s4951

RA-009-C9RA07327B-s4952

RA-009-C9RA07327B-s4953

RA-009-C9RA07327B-s4954

RA-009-C9RA07327B-s4955

RA-009-C9RA07327B-s4956

RA-009-C9RA07327B-s4957

RA-009-C9RA07327B-s4958

RA-009-C9RA07327B-s4959

RA-009-C9RA07327B-s4960

RA-009-C9RA07327B-s4961

RA-009-C9RA07327B-s4962

RA-009-C9RA07327B-s4963

RA-009-C9RA07327B-s4964

RA-009-C9RA07327B-s4965

RA-009-C9RA07327B-s4966

RA-009-C9RA07327B-s4967

RA-009-C9RA07327B-s4968

RA-009-C9RA07327B-s4969

RA-009-C9RA07327B-s4970

RA-009-C9RA07327B-s4971

RA-009-C9RA07327B-s4972

RA-009-C9RA07327B-s4973

RA-009-C9RA07327B-s4974

RA-009-C9RA07327B-s4975

RA-009-C9RA07327B-s4976

RA-009-C9RA07327B-s4977

RA-009-C9RA07327B-s4978

RA-009-C9RA07327B-s4979

RA-009-C9RA07327B-s4980

RA-009-C9RA07327B-s4981

RA-009-C9RA07327B-s4982

RA-009-C9RA07327B-s4983

RA-009-C9RA07327B-s4984

RA-009-C9RA07327B-s4985

RA-009-C9RA07327B-s4986

RA-009-C9RA07327B-s4987

RA-009-C9RA07327B-s4988

RA-009-C9RA07327B-s4989

RA-009-C9RA07327B-s4990

RA-009-C9RA07327B-s4991

RA-009-C9RA07327B-s4992

RA-009-C9RA07327B-s4993

RA-009-C9RA07327B-s4994

RA-009-C9RA07327B-s4995

RA-009-C9RA07327B-s4996

RA-009-C9RA07327B-s4997

RA-009-C9RA07327B-s4998

RA-009-C9RA07327B-s4999

RA-009-C9RA07327B-s5000

RA-009-C9RA07327B-s5001

RA-009-C9RA07327B-s5002

RA-009-C9RA07327B-s5003

RA-009-C9RA07327B-s5004

RA-009-C9RA07327B-s5005

RA-009-C9RA07327B-s5006

RA-009-C9RA07327B-s5007

RA-009-C9RA07327B-s5008

RA-009-C9RA07327B-s5009

RA-009-C9RA07327B-s5010

RA-009-C9RA07327B-s5011

RA-009-C9RA07327B-s5012

RA-009-C9RA07327B-s5013

RA-009-C9RA07327B-s5014

RA-009-C9RA07327B-s5015

RA-009-C9RA07327B-s5016

RA-009-C9RA07327B-s5017

RA-009-C9RA07327B-s5018

RA-009-C9RA07327B-s5019

RA-009-C9RA07327B-s5020

RA-009-C9RA07327B-s5021

RA-009-C9RA07327B-s5022

RA-009-C9RA07327B-s5023

RA-009-C9RA07327B-s5024

RA-009-C9RA07327B-s5025

RA-009-C9RA07327B-s5026

RA-009-C9RA07327B-s5027

RA-009-C9RA07327B-s5028

RA-009-C9RA07327B-s5029

RA-009-C9RA07327B-s5030

RA-009-C9RA07327B-s5031

RA-009-C9RA07327B-s5032

RA-009-C9RA07327B-s5033

RA-009-C9RA07327B-s5034

RA-009-C9RA07327B-s5035

RA-009-C9RA07327B-s5036

RA-009-C9RA07327B-s5037

RA-009-C9RA07327B-s5038

RA-009-C9RA07327B-s5039

RA-009-C9RA07327B-s5040

RA-009-C9RA07327B-s5041

RA-009-C9RA07327B-s5042

RA-009-C9RA07327B-s5043

RA-009-C9RA07327B-s5044

RA-009-C9RA07327B-s5045

RA-009-C9RA07327B-s5046

RA-009-C9RA07327B-s5047

RA-009-C9RA07327B-s5048

RA-009-C9RA07327B-s5049

RA-009-C9RA07327B-s5050

RA-009-C9RA07327B-s5051

RA-009-C9RA07327B-s5052

RA-009-C9RA07327B-s5053

RA-009-C9RA07327B-s5054

RA-009-C9RA07327B-s5055

RA-009-C9RA07327B-s5056

RA-009-C9RA07327B-s5057

RA-009-C9RA07327B-s5058

RA-009-C9RA07327B-s5059

RA-009-C9RA07327B-s5060

RA-009-C9RA07327B-s5061

RA-009-C9RA07327B-s5062

RA-009-C9RA07327B-s5063

RA-009-C9RA07327B-s5064

RA-009-C9RA07327B-s5065

RA-009-C9RA07327B-s5066

RA-009-C9RA07327B-s5067

RA-009-C9RA07327B-s5068

RA-009-C9RA07327B-s5069

RA-009-C9RA07327B-s5070

RA-009-C9RA07327B-s5071

RA-009-C9RA07327B-s5072

RA-009-C9RA07327B-s5073

RA-009-C9RA07327B-s5074

RA-009-C9RA07327B-s5075

RA-009-C9RA07327B-s5076

RA-009-C9RA07327B-s5077

RA-009-C9RA07327B-s5078

RA-009-C9RA07327B-s5079

RA-009-C9RA07327B-s5080

RA-009-C9RA07327B-s5081

RA-009-C9RA07327B-s5082

RA-009-C9RA07327B-s5083

RA-009-C9RA07327B-s5084

RA-009-C9RA07327B-s5085

RA-009-C9RA07327B-s5086

RA-009-C9RA07327B-s5087

RA-009-C9RA07327B-s5088

RA-009-C9RA07327B-s5089

RA-009-C9RA07327B-s5090

RA-009-C9RA07327B-s5091

RA-009-C9RA07327B-s5092

RA-009-C9RA07327B-s5093

RA-009-C9RA07327B-s5094

RA-009-C9RA07327B-s5095

RA-009-C9RA07327B-s5096

RA-009-C9RA07327B-s5097

RA-009-C9RA07327B-s5098

RA-009-C9RA07327B-s5099

RA-009-C9RA07327B-s5100

RA-009-C9RA07327B-s5101

RA-009-C9RA07327B-s5102

RA-009-C9RA07327B-s5103

RA-009-C9RA07327B-s5104

RA-009-C9RA07327B-s5105

RA-009-C9RA07327B-s5106

RA-009-C9RA07327B-s5107

RA-009-C9RA07327B-s5108

RA-009-C9RA07327B-s5109

RA-009-C9RA07327B-s5110

RA-009-C9RA07327B-s5111

RA-009-C9RA07327B-s5112

RA-009-C9RA07327B-s5113

RA-009-C9RA07327B-s5114

RA-009-C9RA07327B-s5115

RA-009-C9RA07327B-s5116

RA-009-C9RA07327B-s5117

RA-009-C9RA07327B-s5118

RA-009-C9RA07327B-s5119

RA-009-C9RA07327B-s5120

RA-009-C9RA07327B-s5121

RA-009-C9RA07327B-s5122

RA-009-C9RA07327B-s5123

RA-009-C9RA07327B-s5124

RA-009-C9RA07327B-s5125

RA-009-C9RA07327B-s5126

RA-009-C9RA07327B-s5127

RA-009-C9RA07327B-s5128

RA-009-C9RA07327B-s5129

RA-009-C9RA07327B-s5130

RA-009-C9RA07327B-s5131

RA-009-C9RA07327B-s5132

RA-009-C9RA07327B-s5133

RA-009-C9RA07327B-s5134

RA-009-C9RA07327B-s5135

RA-009-C9RA07327B-s5136

RA-009-C9RA07327B-s5137

RA-009-C9RA07327B-s5138

RA-009-C9RA07327B-s5139

RA-009-C9RA07327B-s5140

RA-009-C9RA07327B-s5141

RA-009-C9RA07327B-s5142

RA-009-C9RA07327B-s5143

RA-009-C9RA07327B-s5144

RA-009-C9RA07327B-s5145

RA-009-C9RA07327B-s5146

RA-009-C9RA07327B-s5147

RA-009-C9RA07327B-s5148

RA-009-C9RA07327B-s5149

RA-009-C9RA07327B-s5150

RA-009-C9RA07327B-s5151

RA-009-C9RA07327B-s5152

RA-009-C9RA07327B-s5153

RA-009-C9RA07327B-s5154

RA-009-C9RA07327B-s5155

RA-009-C9RA07327B-s5156

RA-009-C9RA07327B-s5157

RA-009-C9RA07327B-s5158

RA-009-C9RA07327B-s5159

RA-009-C9RA07327B-s5160

RA-009-C9RA07327B-s5161

RA-009-C9RA07327B-s5162

RA-009-C9RA07327B-s5163

RA-009-C9RA07327B-s5164

RA-009-C9RA07327B-s5165

RA-009-C9RA07327B-s5166

RA-009-C9RA07327B-s5167

RA-009-C9RA07327B-s5168

RA-009-C9RA07327B-s5169

RA-009-C9RA07327B-s5170

RA-009-C9RA07327B-s5171

RA-009-C9RA07327B-s5172

RA-009-C9RA07327B-s5173

RA-009-C9RA07327B-s5174

RA-009-C9RA07327B-s5175

RA-009-C9RA07327B-s5176

RA-009-C9RA07327B-s5177

RA-009-C9RA07327B-s5178

RA-009-C9RA07327B-s5179

RA-009-C9RA07327B-s5180

RA-009-C9RA07327B-s5181

RA-009-C9RA07327B-s5182

RA-009-C9RA07327B-s5183

RA-009-C9RA07327B-s5184

RA-009-C9RA07327B-s5185

RA-009-C9RA07327B-s5186

RA-009-C9RA07327B-s5187

RA-009-C9RA07327B-s5188

RA-009-C9RA07327B-s5189

RA-009-C9RA07327B-s5190

RA-009-C9RA07327B-s5191

RA-009-C9RA07327B-s5192

RA-009-C9RA07327B-s5193

RA-009-C9RA07327B-s5194

RA-009-C9RA07327B-s5195

RA-009-C9RA07327B-s5196

RA-009-C9RA07327B-s5197

RA-009-C9RA07327B-s5198

RA-009-C9RA07327B-s5199

RA-009-C9RA07327B-s5200

RA-009-C9RA07327B-s5201

RA-009-C9RA07327B-s5202

RA-009-C9RA07327B-s5203

RA-009-C9RA07327B-s5204

RA-009-C9RA07327B-s5205

RA-009-C9RA07327B-s5206

RA-009-C9RA07327B-s5207

RA-009-C9RA07327B-s5208

RA-009-C9RA07327B-s5209

RA-009-C9RA07327B-s5210

RA-009-C9RA07327B-s5211

RA-009-C9RA07327B-s5212

RA-009-C9RA07327B-s5213

RA-009-C9RA07327B-s5214

RA-009-C9RA07327B-s5215

RA-009-C9RA07327B-s5216

RA-009-C9RA07327B-s5217

RA-009-C9RA07327B-s5218

RA-009-C9RA07327B-s5219

RA-009-C9RA07327B-s5220

RA-009-C9RA07327B-s5221

RA-009-C9RA07327B-s5222

RA-009-C9RA07327B-s5223

RA-009-C9RA07327B-s5224

RA-009-C9RA07327B-s5225

RA-009-C9RA07327B-s5226

RA-009-C9RA07327B-s5227

RA-009-C9RA07327B-s5228

RA-009-C9RA07327B-s5229

RA-009-C9RA07327B-s5230

RA-009-C9RA07327B-s5231

RA-009-C9RA07327B-s5232

RA-009-C9RA07327B-s5233

RA-009-C9RA07327B-s5234

RA-009-C9RA07327B-s5235

RA-009-C9RA07327B-s5236

RA-009-C9RA07327B-s5237

RA-009-C9RA07327B-s5238

RA-009-C9RA07327B-s5239

RA-009-C9RA07327B-s5240

RA-009-C9RA07327B-s5241

RA-009-C9RA07327B-s5242

RA-009-C9RA07327B-s5243

RA-009-C9RA07327B-s5244

RA-009-C9RA07327B-s5245

RA-009-C9RA07327B-s5246

RA-009-C9RA07327B-s5247

RA-009-C9RA07327B-s5248

RA-009-C9RA07327B-s5249

RA-009-C9RA07327B-s5250

RA-009-C9RA07327B-s5251

RA-009-C9RA07327B-s5252

RA-009-C9RA07327B-s5253

RA-009-C9RA07327B-s5254

RA-009-C9RA07327B-s5255

RA-009-C9RA07327B-s5256

RA-009-C9RA07327B-s5257

RA-009-C9RA07327B-s5258

RA-009-C9RA07327B-s5259

RA-009-C9RA07327B-s5260

RA-009-C9RA07327B-s5261

RA-009-C9RA07327B-s5262

RA-009-C9RA07327B-s5263

RA-009-C9RA07327B-s5264

RA-009-C9RA07327B-s5265

RA-009-C9RA07327B-s5266

RA-009-C9RA07327B-s5267

RA-009-C9RA07327B-s5268

RA-009-C9RA07327B-s5269

RA-009-C9RA07327B-s5270

RA-009-C9RA07327B-s5271

RA-009-C9RA07327B-s5272

RA-009-C9RA07327B-s5273

RA-009-C9RA07327B-s5274

RA-009-C9RA07327B-s5275

RA-009-C9RA07327B-s5276

RA-009-C9RA07327B-s5277

RA-009-C9RA07327B-s5278

RA-009-C9RA07327B-s5279

RA-009-C9RA07327B-s5280

RA-009-C9RA07327B-s5281

RA-009-C9RA07327B-s5282

RA-009-C9RA07327B-s5283

RA-009-C9RA07327B-s5284

RA-009-C9RA07327B-s5285

RA-009-C9RA07327B-s5286

RA-009-C9RA07327B-s5287

RA-009-C9RA07327B-s5288

RA-009-C9RA07327B-s5289

RA-009-C9RA07327B-s5290

RA-009-C9RA07327B-s5291

RA-009-C9RA07327B-s5292

RA-009-C9RA07327B-s5293

RA-009-C9RA07327B-s5294

RA-009-C9RA07327B-s5295

RA-009-C9RA07327B-s5296

RA-009-C9RA07327B-s5297

RA-009-C9RA07327B-s5298

RA-009-C9RA07327B-s5299

RA-009-C9RA07327B-s5300

RA-009-C9RA07327B-s5301

RA-009-C9RA07327B-s5302

RA-009-C9RA07327B-s5303

RA-009-C9RA07327B-s5304

RA-009-C9RA07327B-s5305

RA-009-C9RA07327B-s5306

RA-009-C9RA07327B-s5307

RA-009-C9RA07327B-s5308

RA-009-C9RA07327B-s5309

RA-009-C9RA07327B-s5310

RA-009-C9RA07327B-s5311

RA-009-C9RA07327B-s5312

RA-009-C9RA07327B-s5313

RA-009-C9RA07327B-s5314

RA-009-C9RA07327B-s5315

RA-009-C9RA07327B-s5316

RA-009-C9RA07327B-s5317

RA-009-C9RA07327B-s5318

RA-009-C9RA07327B-s5319

RA-009-C9RA07327B-s5320

RA-009-C9RA07327B-s5321

RA-009-C9RA07327B-s5322

RA-009-C9RA07327B-s5323

RA-009-C9RA07327B-s5324

RA-009-C9RA07327B-s5325

RA-009-C9RA07327B-s5326

RA-009-C9RA07327B-s5327

RA-009-C9RA07327B-s5328

RA-009-C9RA07327B-s5329

RA-009-C9RA07327B-s5330

RA-009-C9RA07327B-s5331

RA-009-C9RA07327B-s5332

RA-009-C9RA07327B-s5333

RA-009-C9RA07327B-s5334

RA-009-C9RA07327B-s5335

RA-009-C9RA07327B-s5336

RA-009-C9RA07327B-s5337

RA-009-C9RA07327B-s5338

RA-009-C9RA07327B-s5339

RA-009-C9RA07327B-s5340

RA-009-C9RA07327B-s5341

RA-009-C9RA07327B-s5342

RA-009-C9RA07327B-s5343

RA-009-C9RA07327B-s5344

RA-009-C9RA07327B-s5345

RA-009-C9RA07327B-s5346

RA-009-C9RA07327B-s5347

RA-009-C9RA07327B-s5348

RA-009-C9RA07327B-s5349

RA-009-C9RA07327B-s5350

RA-009-C9RA07327B-s5351

RA-009-C9RA07327B-s5352

RA-009-C9RA07327B-s5353

RA-009-C9RA07327B-s5354

RA-009-C9RA07327B-s5355

RA-009-C9RA07327B-s5356

RA-009-C9RA07327B-s5357

RA-009-C9RA07327B-s5358

RA-009-C9RA07327B-s5359

RA-009-C9RA07327B-s5360

RA-009-C9RA07327B-s5361

RA-009-C9RA07327B-s5362

RA-009-C9RA07327B-s5363

RA-009-C9RA07327B-s5364

RA-009-C9RA07327B-s5365

RA-009-C9RA07327B-s5366

RA-009-C9RA07327B-s5367

RA-009-C9RA07327B-s5368

RA-009-C9RA07327B-s5369

RA-009-C9RA07327B-s5370

RA-009-C9RA07327B-s5371

RA-009-C9RA07327B-s5372

RA-009-C9RA07327B-s5373

RA-009-C9RA07327B-s5374

RA-009-C9RA07327B-s5375

RA-009-C9RA07327B-s5376

RA-009-C9RA07327B-s5377

RA-009-C9RA07327B-s5378

RA-009-C9RA07327B-s5379

RA-009-C9RA07327B-s5380

RA-009-C9RA07327B-s5381

RA-009-C9RA07327B-s5382

RA-009-C9RA07327B-s5383

RA-009-C9RA07327B-s5384

RA-009-C9RA07327B-s5385

RA-009-C9RA07327B-s5386

RA-009-C9RA07327B-s5387

RA-009-C9RA07327B-s5388

RA-009-C9RA07327B-s5389

RA-009-C9RA07327B-s5390

RA-009-C9RA07327B-s5391

RA-009-C9RA07327B-s5392

RA-009-C9RA07327B-s5393

RA-009-C9RA07327B-s5394

RA-009-C9RA07327B-s5395

RA-009-C9RA07327B-s5396

RA-009-C9RA07327B-s5397

RA-009-C9RA07327B-s5398

RA-009-C9RA07327B-s5399

RA-009-C9RA07327B-s5400

RA-009-C9RA07327B-s5401

RA-009-C9RA07327B-s5402

RA-009-C9RA07327B-s5403

RA-009-C9RA07327B-s5404

RA-009-C9RA07327B-s5405

RA-009-C9RA07327B-s5406

RA-009-C9RA07327B-s5407

RA-009-C9RA07327B-s5408

RA-009-C9RA07327B-s5409

RA-009-C9RA07327B-s5410

RA-009-C9RA07327B-s5411

RA-009-C9RA07327B-s5412

RA-009-C9RA07327B-s5413

RA-009-C9RA07327B-s5414

RA-009-C9RA07327B-s5415

RA-009-C9RA07327B-s5416

RA-009-C9RA07327B-s5417

RA-009-C9RA07327B-s5418

RA-009-C9RA07327B-s5419

RA-009-C9RA07327B-s5420

RA-009-C9RA07327B-s5421

RA-009-C9RA07327B-s5422

RA-009-C9RA07327B-s5423

RA-009-C9RA07327B-s5424

RA-009-C9RA07327B-s5425

RA-009-C9RA07327B-s5426

RA-009-C9RA07327B-s5427

RA-009-C9RA07327B-s5428

RA-009-C9RA07327B-s5429

RA-009-C9RA07327B-s5430

RA-009-C9RA07327B-s5431

RA-009-C9RA07327B-s5432

RA-009-C9RA07327B-s5433

RA-009-C9RA07327B-s5434

RA-009-C9RA07327B-s5435

RA-009-C9RA07327B-s5436

RA-009-C9RA07327B-s5437

RA-009-C9RA07327B-s5438

RA-009-C9RA07327B-s5439

RA-009-C9RA07327B-s5440

RA-009-C9RA07327B-s5441

RA-009-C9RA07327B-s5442

RA-009-C9RA07327B-s5443

RA-009-C9RA07327B-s5444

RA-009-C9RA07327B-s5445

RA-009-C9RA07327B-s5446

RA-009-C9RA07327B-s5447

RA-009-C9RA07327B-s5448

RA-009-C9RA07327B-s5449

RA-009-C9RA07327B-s5450

RA-009-C9RA07327B-s5451

RA-009-C9RA07327B-s5452

RA-009-C9RA07327B-s5453

RA-009-C9RA07327B-s5454

RA-009-C9RA07327B-s5455

RA-009-C9RA07327B-s5456

RA-009-C9RA07327B-s5457

RA-009-C9RA07327B-s5458

RA-009-C9RA07327B-s5459

RA-009-C9RA07327B-s5460

RA-009-C9RA07327B-s5461

RA-009-C9RA07327B-s5462

RA-009-C9RA07327B-s5463

RA-009-C9RA07327B-s5464

RA-009-C9RA07327B-s5465

RA-009-C9RA07327B-s5466

RA-009-C9RA07327B-s5467

RA-009-C9RA07327B-s5468

RA-009-C9RA07327B-s5469

RA-009-C9RA07327B-s5470

RA-009-C9RA07327B-s5471

RA-009-C9RA07327B-s5472

RA-009-C9RA07327B-s5473

RA-009-C9RA07327B-s5474

RA-009-C9RA07327B-s5475

RA-009-C9RA07327B-s5476

RA-009-C9RA07327B-s5477

RA-009-C9RA07327B-s5478

RA-009-C9RA07327B-s5479

RA-009-C9RA07327B-s5480

RA-009-C9RA07327B-s5481

RA-009-C9RA07327B-s5482

RA-009-C9RA07327B-s5483

RA-009-C9RA07327B-s5484

RA-009-C9RA07327B-s5485

RA-009-C9RA07327B-s5486

RA-009-C9RA07327B-s5487

RA-009-C9RA07327B-s5488

RA-009-C9RA07327B-s5489

RA-009-C9RA07327B-s5490

RA-009-C9RA07327B-s5491

RA-009-C9RA07327B-s5492

RA-009-C9RA07327B-s5493

RA-009-C9RA07327B-s5494

RA-009-C9RA07327B-s5495

RA-009-C9RA07327B-s5496

RA-009-C9RA07327B-s5497

RA-009-C9RA07327B-s5498

RA-009-C9RA07327B-s5499

RA-009-C9RA07327B-s5500

RA-009-C9RA07327B-s5501

RA-009-C9RA07327B-s5502

RA-009-C9RA07327B-s5503

RA-009-C9RA07327B-s5504

RA-009-C9RA07327B-s5505

RA-009-C9RA07327B-s5506

RA-009-C9RA07327B-s5507

RA-009-C9RA07327B-s5508

RA-009-C9RA07327B-s5509

RA-009-C9RA07327B-s5510

RA-009-C9RA07327B-s5511

RA-009-C9RA07327B-s5512

RA-009-C9RA07327B-s5513

RA-009-C9RA07327B-s5514

RA-009-C9RA07327B-s5515

RA-009-C9RA07327B-s5516

RA-009-C9RA07327B-s5517

RA-009-C9RA07327B-s5518

RA-009-C9RA07327B-s5519

RA-009-C9RA07327B-s5520

RA-009-C9RA07327B-s5521

RA-009-C9RA07327B-s5522

RA-009-C9RA07327B-s5523

RA-009-C9RA07327B-s5524

RA-009-C9RA07327B-s5525

RA-009-C9RA07327B-s5526

RA-009-C9RA07327B-s5527

RA-009-C9RA07327B-s5528

RA-009-C9RA07327B-s5529

RA-009-C9RA07327B-s5530

RA-009-C9RA07327B-s5531

RA-009-C9RA07327B-s5532

RA-009-C9RA07327B-s5533

RA-009-C9RA07327B-s5534

RA-009-C9RA07327B-s5535

RA-009-C9RA07327B-s5536

RA-009-C9RA07327B-s5537

RA-009-C9RA07327B-s5538

RA-009-C9RA07327B-s5539

RA-009-C9RA07327B-s5540

RA-009-C9RA07327B-s5541

RA-009-C9RA07327B-s5542

RA-009-C9RA07327B-s5543

RA-009-C9RA07327B-s5544

RA-009-C9RA07327B-s5545

RA-009-C9RA07327B-s5546

RA-009-C9RA07327B-s5547

RA-009-C9RA07327B-s5548

RA-009-C9RA07327B-s5549

RA-009-C9RA07327B-s5550

RA-009-C9RA07327B-s5551

RA-009-C9RA07327B-s5552

RA-009-C9RA07327B-s5553

RA-009-C9RA07327B-s5554

RA-009-C9RA07327B-s5555

RA-009-C9RA07327B-s5556

RA-009-C9RA07327B-s5557

RA-009-C9RA07327B-s5558

RA-009-C9RA07327B-s5559

RA-009-C9RA07327B-s5560

RA-009-C9RA07327B-s5561

RA-009-C9RA07327B-s5562

RA-009-C9RA07327B-s5563

RA-009-C9RA07327B-s5564

RA-009-C9RA07327B-s5565

RA-009-C9RA07327B-s5566

RA-009-C9RA07327B-s5567

RA-009-C9RA07327B-s5568

RA-009-C9RA07327B-s5569

RA-009-C9RA07327B-s5570

RA-009-C9RA07327B-s5571

RA-009-C9RA07327B-s5572

RA-009-C9RA07327B-s5573

RA-009-C9RA07327B-s5574

RA-009-C9RA07327B-s5575

RA-009-C9RA07327B-s5576

RA-009-C9RA07327B-s5577

RA-009-C9RA07327B-s5578

RA-009-C9RA07327B-s5579

RA-009-C9RA07327B-s5580

RA-009-C9RA07327B-s5581

RA-009-C9RA07327B-s5582

RA-009-C9RA07327B-s5583

RA-009-C9RA07327B-s5584

RA-009-C9RA07327B-s5585

RA-009-C9RA07327B-s5586

RA-009-C9RA07327B-s5587

RA-009-C9RA07327B-s5588

RA-009-C9RA07327B-s5589

RA-009-C9RA07327B-s5590

RA-009-C9RA07327B-s5591

RA-009-C9RA07327B-s5592

RA-009-C9RA07327B-s5593

RA-009-C9RA07327B-s5594

RA-009-C9RA07327B-s5595

RA-009-C9RA07327B-s5596

RA-009-C9RA07327B-s5597

RA-009-C9RA07327B-s5598

RA-009-C9RA07327B-s5599

RA-009-C9RA07327B-s5600

RA-009-C9RA07327B-s5601

RA-009-C9RA07327B-s5602

RA-009-C9RA07327B-s5603

RA-009-C9RA07327B-s5604

RA-009-C9RA07327B-s5605

RA-009-C9RA07327B-s5606

RA-009-C9RA07327B-s5607

RA-009-C9RA07327B-s5608

RA-009-C9RA07327B-s5609

RA-009-C9RA07327B-s5610

RA-009-C9RA07327B-s5611

RA-009-C9RA07327B-s5612

RA-009-C9RA07327B-s5613

RA-009-C9RA07327B-s5614

RA-009-C9RA07327B-s5615

RA-009-C9RA07327B-s5616

RA-009-C9RA07327B-s5617

RA-009-C9RA07327B-s5618

RA-009-C9RA07327B-s5619

RA-009-C9RA07327B-s5620

RA-009-C9RA07327B-s5621

RA-009-C9RA07327B-s5622

RA-009-C9RA07327B-s5623

RA-009-C9RA07327B-s5624

RA-009-C9RA07327B-s5625

RA-009-C9RA07327B-s5626

RA-009-C9RA07327B-s5627

RA-009-C9RA07327B-s5628

RA-009-C9RA07327B-s5629

RA-009-C9RA07327B-s5630

RA-009-C9RA07327B-s5631

RA-009-C9RA07327B-s5632

RA-009-C9RA07327B-s5633

RA-009-C9RA07327B-s5634

RA-009-C9RA07327B-s5635

RA-009-C9RA07327B-s5636

RA-009-C9RA07327B-s5637

RA-009-C9RA07327B-s5638

RA-009-C9RA07327B-s5639

RA-009-C9RA07327B-s5640

RA-009-C9RA07327B-s5641

RA-009-C9RA07327B-s5642

RA-009-C9RA07327B-s5643

RA-009-C9RA07327B-s5644

RA-009-C9RA07327B-s5645

RA-009-C9RA07327B-s5646

RA-009-C9RA07327B-s5647

RA-009-C9RA07327B-s5648

RA-009-C9RA07327B-s5649

RA-009-C9RA07327B-s5650

RA-009-C9RA07327B-s5651

RA-009-C9RA07327B-s5652

RA-009-C9RA07327B-s5653

RA-009-C9RA07327B-s5654

RA-009-C9RA07327B-s5655

RA-009-C9RA07327B-s5656

RA-009-C9RA07327B-s5657

RA-009-C9RA07327B-s5658

RA-009-C9RA07327B-s5659

RA-009-C9RA07327B-s5660

RA-009-C9RA07327B-s5661

RA-009-C9RA07327B-s5662

RA-009-C9RA07327B-s5663

RA-009-C9RA07327B-s5664

RA-009-C9RA07327B-s5665

RA-009-C9RA07327B-s5666

RA-009-C9RA07327B-s5667

RA-009-C9RA07327B-s5668

RA-009-C9RA07327B-s5669

RA-009-C9RA07327B-s5670

RA-009-C9RA07327B-s5671

RA-009-C9RA07327B-s5672

RA-009-C9RA07327B-s5673

RA-009-C9RA07327B-s5674

RA-009-C9RA07327B-s5675

RA-009-C9RA07327B-s5676

RA-009-C9RA07327B-s5677

RA-009-C9RA07327B-s5678

RA-009-C9RA07327B-s5679

RA-009-C9RA07327B-s5680

RA-009-C9RA07327B-s5681

RA-009-C9RA07327B-s5682

RA-009-C9RA07327B-s5683

RA-009-C9RA07327B-s5684

RA-009-C9RA07327B-s5685

RA-009-C9RA07327B-s5686

RA-009-C9RA07327B-s5687

RA-009-C9RA07327B-s5688

RA-009-C9RA07327B-s5689

RA-009-C9RA07327B-s5690

RA-009-C9RA07327B-s5691

RA-009-C9RA07327B-s5692

RA-009-C9RA07327B-s5693

RA-009-C9RA07327B-s5694

RA-009-C9RA07327B-s5695

RA-009-C9RA07327B-s5696

RA-009-C9RA07327B-s5697

RA-009-C9RA07327B-s5698

RA-009-C9RA07327B-s5699

RA-009-C9RA07327B-s5700

RA-009-C9RA07327B-s5701

RA-009-C9RA07327B-s5702

RA-009-C9RA07327B-s5703

RA-009-C9RA07327B-s5704

RA-009-C9RA07327B-s5705

RA-009-C9RA07327B-s5706

RA-009-C9RA07327B-s5707

RA-009-C9RA07327B-s5708

RA-009-C9RA07327B-s5709

RA-009-C9RA07327B-s5710

RA-009-C9RA07327B-s5711

RA-009-C9RA07327B-s5712

RA-009-C9RA07327B-s5713

RA-009-C9RA07327B-s5714

RA-009-C9RA07327B-s5715

RA-009-C9RA07327B-s5716

RA-009-C9RA07327B-s5717

RA-009-C9RA07327B-s5718

RA-009-C9RA07327B-s5719

RA-009-C9RA07327B-s5720

RA-009-C9RA07327B-s5721

RA-009-C9RA07327B-s5722

RA-009-C9RA07327B-s5723

RA-009-C9RA07327B-s5724

RA-009-C9RA07327B-s5725

RA-009-C9RA07327B-s5726

RA-009-C9RA07327B-s5727

RA-009-C9RA07327B-s5728

RA-009-C9RA07327B-s5729

RA-009-C9RA07327B-s5730

RA-009-C9RA07327B-s5731

RA-009-C9RA07327B-s5732

RA-009-C9RA07327B-s5733

RA-009-C9RA07327B-s5734

RA-009-C9RA07327B-s5735

RA-009-C9RA07327B-s5736

RA-009-C9RA07327B-s5737

RA-009-C9RA07327B-s5738

RA-009-C9RA07327B-s5739

RA-009-C9RA07327B-s5740

RA-009-C9RA07327B-s5741

RA-009-C9RA07327B-s5742

RA-009-C9RA07327B-s5743

RA-009-C9RA07327B-s5744

RA-009-C9RA07327B-s5745

RA-009-C9RA07327B-s5746

RA-009-C9RA07327B-s5747

RA-009-C9RA07327B-s5748

RA-009-C9RA07327B-s5749

RA-009-C9RA07327B-s5750

RA-009-C9RA07327B-s5751

RA-009-C9RA07327B-s5752

RA-009-C9RA07327B-s5753

RA-009-C9RA07327B-s5754

RA-009-C9RA07327B-s5755

RA-009-C9RA07327B-s5756

RA-009-C9RA07327B-s5757

RA-009-C9RA07327B-s5758

RA-009-C9RA07327B-s5759

RA-009-C9RA07327B-s5760

RA-009-C9RA07327B-s5761

RA-009-C9RA07327B-s5762

RA-009-C9RA07327B-s5763

RA-009-C9RA07327B-s5764

RA-009-C9RA07327B-s5765

RA-009-C9RA07327B-s5766

RA-009-C9RA07327B-s5767

RA-009-C9RA07327B-s5768

RA-009-C9RA07327B-s5769

RA-009-C9RA07327B-s5770

RA-009-C9RA07327B-s5771

RA-009-C9RA07327B-s5772

RA-009-C9RA07327B-s5773

RA-009-C9RA07327B-s5774

RA-009-C9RA07327B-s5775

RA-009-C9RA07327B-s5776

RA-009-C9RA07327B-s5777

RA-009-C9RA07327B-s5778

RA-009-C9RA07327B-s5779

RA-009-C9RA07327B-s5780

RA-009-C9RA07327B-s5781

RA-009-C9RA07327B-s5782

RA-009-C9RA07327B-s5783

RA-009-C9RA07327B-s5784

RA-009-C9RA07327B-s5785

RA-009-C9RA07327B-s5786

RA-009-C9RA07327B-s5787

RA-009-C9RA07327B-s5788

RA-009-C9RA07327B-s5789

RA-009-C9RA07327B-s5790

RA-009-C9RA07327B-s5791

RA-009-C9RA07327B-s5792

RA-009-C9RA07327B-s5793

RA-009-C9RA07327B-s5794

RA-009-C9RA07327B-s5795

RA-009-C9RA07327B-s5796

RA-009-C9RA07327B-s5797

RA-009-C9RA07327B-s5798

RA-009-C9RA07327B-s5799

RA-009-C9RA07327B-s5800

RA-009-C9RA07327B-s5801

RA-009-C9RA07327B-s5802

RA-009-C9RA07327B-s5803

RA-009-C9RA07327B-s5804

RA-009-C9RA07327B-s5805

RA-009-C9RA07327B-s5806

RA-009-C9RA07327B-s5807

RA-009-C9RA07327B-s5808

RA-009-C9RA07327B-s5809

RA-009-C9RA07327B-s5810

RA-009-C9RA07327B-s5811

RA-009-C9RA07327B-s5812

RA-009-C9RA07327B-s5813

RA-009-C9RA07327B-s5814

RA-009-C9RA07327B-s5815

RA-009-C9RA07327B-s5816

RA-009-C9RA07327B-s5817

RA-009-C9RA07327B-s5818

RA-009-C9RA07327B-s5819

RA-009-C9RA07327B-s5820

RA-009-C9RA07327B-s5821

RA-009-C9RA07327B-s5822

RA-009-C9RA07327B-s5823

RA-009-C9RA07327B-s5824

RA-009-C9RA07327B-s5825

RA-009-C9RA07327B-s5826

RA-009-C9RA07327B-s5827

RA-009-C9RA07327B-s5828

RA-009-C9RA07327B-s5829

RA-009-C9RA07327B-s5830

RA-009-C9RA07327B-s5831

RA-009-C9RA07327B-s5832

RA-009-C9RA07327B-s5833

RA-009-C9RA07327B-s5834

RA-009-C9RA07327B-s5835

RA-009-C9RA07327B-s5836

RA-009-C9RA07327B-s5837

RA-009-C9RA07327B-s5838

RA-009-C9RA07327B-s5839

RA-009-C9RA07327B-s5840

RA-009-C9RA07327B-s5841

RA-009-C9RA07327B-s5842

RA-009-C9RA07327B-s5843

RA-009-C9RA07327B-s5844

RA-009-C9RA07327B-s5845

RA-009-C9RA07327B-s5846

RA-009-C9RA07327B-s5847

RA-009-C9RA07327B-s5848

RA-009-C9RA07327B-s5849

RA-009-C9RA07327B-s5850

RA-009-C9RA07327B-s5851

RA-009-C9RA07327B-s5852

RA-009-C9RA07327B-s5853

RA-009-C9RA07327B-s5854

RA-009-C9RA07327B-s5855

RA-009-C9RA07327B-s5856

RA-009-C9RA07327B-s5857

RA-009-C9RA07327B-s5858

RA-009-C9RA07327B-s5859

RA-009-C9RA07327B-s5860

RA-009-C9RA07327B-s5861

RA-009-C9RA07327B-s5862

RA-009-C9RA07327B-s5863

RA-009-C9RA07327B-s5864

RA-009-C9RA07327B-s5865

RA-009-C9RA07327B-s5866

RA-009-C9RA07327B-s5867

RA-009-C9RA07327B-s5868

RA-009-C9RA07327B-s5869

RA-009-C9RA07327B-s5870

RA-009-C9RA07327B-s5871

RA-009-C9RA07327B-s5872

RA-009-C9RA07327B-s5873

RA-009-C9RA07327B-s5874

RA-009-C9RA07327B-s5875

RA-009-C9RA07327B-s5876

RA-009-C9RA07327B-s5877

RA-009-C9RA07327B-s5878

RA-009-C9RA07327B-s5879

RA-009-C9RA07327B-s5880

RA-009-C9RA07327B-s5881

RA-009-C9RA07327B-s5882

RA-009-C9RA07327B-s5883

RA-009-C9RA07327B-s5884

RA-009-C9RA07327B-s5885

RA-009-C9RA07327B-s5886

RA-009-C9RA07327B-s5887

RA-009-C9RA07327B-s5888

RA-009-C9RA07327B-s5889

RA-009-C9RA07327B-s5890

RA-009-C9RA07327B-s5891

RA-009-C9RA07327B-s5892

RA-009-C9RA07327B-s5893

RA-009-C9RA07327B-s5894

RA-009-C9RA07327B-s5895

RA-009-C9RA07327B-s5896

RA-009-C9RA07327B-s5897

RA-009-C9RA07327B-s5898

RA-009-C9RA07327B-s5899

RA-009-C9RA07327B-s5900

RA-009-C9RA07327B-s5901

RA-009-C9RA07327B-s5902

RA-009-C9RA07327B-s5903

RA-009-C9RA07327B-s5904

RA-009-C9RA07327B-s5905

RA-009-C9RA07327B-s5906

RA-009-C9RA07327B-s5907

RA-009-C9RA07327B-s5908

RA-009-C9RA07327B-s5909

RA-009-C9RA07327B-s5910

RA-009-C9RA07327B-s5911

RA-009-C9RA07327B-s5912

RA-009-C9RA07327B-s5913

RA-009-C9RA07327B-s5914

RA-009-C9RA07327B-s5915

RA-009-C9RA07327B-s5916

RA-009-C9RA07327B-s5917

RA-009-C9RA07327B-s5918

RA-009-C9RA07327B-s5919

RA-009-C9RA07327B-s5920

RA-009-C9RA07327B-s5921

RA-009-C9RA07327B-s5922

RA-009-C9RA07327B-s5923

RA-009-C9RA07327B-s5924

RA-009-C9RA07327B-s5925

RA-009-C9RA07327B-s5926

RA-009-C9RA07327B-s5927

RA-009-C9RA07327B-s5928

RA-009-C9RA07327B-s5929

RA-009-C9RA07327B-s5930

RA-009-C9RA07327B-s5931

RA-009-C9RA07327B-s5932

RA-009-C9RA07327B-s5933

RA-009-C9RA07327B-s5934

RA-009-C9RA07327B-s5935

RA-009-C9RA07327B-s5936

RA-009-C9RA07327B-s5937

RA-009-C9RA07327B-s5938

RA-009-C9RA07327B-s5939

RA-009-C9RA07327B-s5940

RA-009-C9RA07327B-s5941

RA-009-C9RA07327B-s5942

RA-009-C9RA07327B-s5943

RA-009-C9RA07327B-s5944

RA-009-C9RA07327B-s5945

RA-009-C9RA07327B-s5946

RA-009-C9RA07327B-s5947

RA-009-C9RA07327B-s5948

RA-009-C9RA07327B-s5949

RA-009-C9RA07327B-s5950

RA-009-C9RA07327B-s5951

RA-009-C9RA07327B-s5952

RA-009-C9RA07327B-s5953

RA-009-C9RA07327B-s5954

RA-009-C9RA07327B-s5955

RA-009-C9RA07327B-s5956

RA-009-C9RA07327B-s5957

RA-009-C9RA07327B-s5958

RA-009-C9RA07327B-s5959

RA-009-C9RA07327B-s5960

RA-009-C9RA07327B-s5961

RA-009-C9RA07327B-s5962

RA-009-C9RA07327B-s5963

RA-009-C9RA07327B-s5964

RA-009-C9RA07327B-s5965

RA-009-C9RA07327B-s5966

RA-009-C9RA07327B-s5967

RA-009-C9RA07327B-s5968

RA-009-C9RA07327B-s5969

RA-009-C9RA07327B-s5970

RA-009-C9RA07327B-s5971

RA-009-C9RA07327B-s5972

RA-009-C9RA07327B-s5973

RA-009-C9RA07327B-s5974

RA-009-C9RA07327B-s5975

RA-009-C9RA07327B-s5976

RA-009-C9RA07327B-s5977

RA-009-C9RA07327B-s5978

RA-009-C9RA07327B-s5979

RA-009-C9RA07327B-s5980

RA-009-C9RA07327B-s5981

RA-009-C9RA07327B-s5982

RA-009-C9RA07327B-s5983

RA-009-C9RA07327B-s5984

RA-009-C9RA07327B-s5985

RA-009-C9RA07327B-s5986

RA-009-C9RA07327B-s5987

RA-009-C9RA07327B-s5988

RA-009-C9RA07327B-s5989

RA-009-C9RA07327B-s5990

RA-009-C9RA07327B-s5991

RA-009-C9RA07327B-s5992

RA-009-C9RA07327B-s5993

RA-009-C9RA07327B-s5994

RA-009-C9RA07327B-s5995

RA-009-C9RA07327B-s5996

RA-009-C9RA07327B-s5997

RA-009-C9RA07327B-s5998

RA-009-C9RA07327B-s5999

RA-009-C9RA07327B-s6000

RA-009-C9RA07327B-s6001

RA-009-C9RA07327B-s6002

RA-009-C9RA07327B-s6003

RA-009-C9RA07327B-s6004

RA-009-C9RA07327B-s6005

RA-009-C9RA07327B-s6006

RA-009-C9RA07327B-s6007

RA-009-C9RA07327B-s6008

RA-009-C9RA07327B-s6009

RA-009-C9RA07327B-s6010

RA-009-C9RA07327B-s6011

RA-009-C9RA07327B-s6012

RA-009-C9RA07327B-s6013

RA-009-C9RA07327B-s6014

RA-009-C9RA07327B-s6015

RA-009-C9RA07327B-s6016

RA-009-C9RA07327B-s6017

RA-009-C9RA07327B-s6018

RA-009-C9RA07327B-s6019

RA-009-C9RA07327B-s6020

RA-009-C9RA07327B-s6021

RA-009-C9RA07327B-s6022

RA-009-C9RA07327B-s6023

RA-009-C9RA07327B-s6024

RA-009-C9RA07327B-s6025

RA-009-C9RA07327B-s6026

RA-009-C9RA07327B-s6027

RA-009-C9RA07327B-s6028

RA-009-C9RA07327B-s6029

RA-009-C9RA07327B-s6030

RA-009-C9RA07327B-s6031

RA-009-C9RA07327B-s6032

RA-009-C9RA07327B-s6033

RA-009-C9RA07327B-s6034

RA-009-C9RA07327B-s6035

RA-009-C9RA07327B-s6036

RA-009-C9RA07327B-s6037

RA-009-C9RA07327B-s6038

RA-009-C9RA07327B-s6039

RA-009-C9RA07327B-s6040

RA-009-C9RA07327B-s6041

RA-009-C9RA07327B-s6042

RA-009-C9RA07327B-s6043

RA-009-C9RA07327B-s6044

RA-009-C9RA07327B-s6045

RA-009-C9RA07327B-s6046

RA-009-C9RA07327B-s6047

RA-009-C9RA07327B-s6048

RA-009-C9RA07327B-s6049

RA-009-C9RA07327B-s6050

RA-009-C9RA07327B-s6051

RA-009-C9RA07327B-s6052

RA-009-C9RA07327B-s6053

RA-009-C9RA07327B-s6054

RA-009-C9RA07327B-s6055

RA-009-C9RA07327B-s6056

RA-009-C9RA07327B-s6057

RA-009-C9RA07327B-s6058

RA-009-C9RA07327B-s6059

RA-009-C9RA07327B-s6060

RA-009-C9RA07327B-s6061

RA-009-C9RA07327B-s6062

RA-009-C9RA07327B-s6063

RA-009-C9RA07327B-s6064

RA-009-C9RA07327B-s6065

RA-009-C9RA07327B-s6066

RA-009-C9RA07327B-s6067

RA-009-C9RA07327B-s6068

RA-009-C9RA07327B-s6069

RA-009-C9RA07327B-s6070

RA-009-C9RA07327B-s6071

RA-009-C9RA07327B-s6072

RA-009-C9RA07327B-s6073

RA-009-C9RA07327B-s6074

RA-009-C9RA07327B-s6075

RA-009-C9RA07327B-s6076

RA-009-C9RA07327B-s6077

RA-009-C9RA07327B-s6078

RA-009-C9RA07327B-s6079

RA-009-C9RA07327B-s6080

RA-009-C9RA07327B-s6081

RA-009-C9RA07327B-s6082

RA-009-C9RA07327B-s6083

RA-009-C9RA07327B-s6084

RA-009-C9RA07327B-s6085

RA-009-C9RA07327B-s6086

RA-009-C9RA07327B-s6087

RA-009-C9RA07327B-s6088

RA-009-C9RA07327B-s6089

RA-009-C9RA07327B-s6090

RA-009-C9RA07327B-s6091

RA-009-C9RA07327B-s6092

RA-009-C9RA07327B-s6093

RA-009-C9RA07327B-s6094

RA-009-C9RA07327B-s6095

RA-009-C9RA07327B-s6096

RA-009-C9RA07327B-s6097

RA-009-C9RA07327B-s6098

RA-009-C9RA07327B-s6099

RA-009-C9RA07327B-s6100

RA-009-C9RA07327B-s6101

RA-009-C9RA07327B-s6102

RA-009-C9RA07327B-s6103

RA-009-C9RA07327B-s6104

RA-009-C9RA07327B-s6105

RA-009-C9RA07327B-s6106

RA-009-C9RA07327B-s6107

RA-009-C9RA07327B-s6108

RA-009-C9RA07327B-s6109

RA-009-C9RA07327B-s6110

RA-009-C9RA07327B-s6111

RA-009-C9RA07327B-s6112

RA-009-C9RA07327B-s6113

RA-009-C9RA07327B-s6114

RA-009-C9RA07327B-s6115

RA-009-C9RA07327B-s6116

RA-009-C9RA07327B-s6117

RA-009-C9RA07327B-s6118

RA-009-C9RA07327B-s6119

RA-009-C9RA07327B-s6120

RA-009-C9RA07327B-s6121

RA-009-C9RA07327B-s6122

RA-009-C9RA07327B-s6123

RA-009-C9RA07327B-s6124

RA-009-C9RA07327B-s6125

RA-009-C9RA07327B-s6126

RA-009-C9RA07327B-s6127

RA-009-C9RA07327B-s6128

RA-009-C9RA07327B-s6129

RA-009-C9RA07327B-s6130

RA-009-C9RA07327B-s6131

RA-009-C9RA07327B-s6132

RA-009-C9RA07327B-s6133

RA-009-C9RA07327B-s6134

RA-009-C9RA07327B-s6135

RA-009-C9RA07327B-s6136

RA-009-C9RA07327B-s6137

RA-009-C9RA07327B-s6138

RA-009-C9RA07327B-s6139

RA-009-C9RA07327B-s6140

RA-009-C9RA07327B-s6141

RA-009-C9RA07327B-s6142

RA-009-C9RA07327B-s6143

RA-009-C9RA07327B-s6144

RA-009-C9RA07327B-s6145

RA-009-C9RA07327B-s6146

RA-009-C9RA07327B-s6147

RA-009-C9RA07327B-s6148

RA-009-C9RA07327B-s6149

RA-009-C9RA07327B-s6150

RA-009-C9RA07327B-s6151

RA-009-C9RA07327B-s6152

RA-009-C9RA07327B-s6153

RA-009-C9RA07327B-s6154

RA-009-C9RA07327B-s6155

RA-009-C9RA07327B-s6156

RA-009-C9RA07327B-s6157

RA-009-C9RA07327B-s6158

RA-009-C9RA07327B-s6159

RA-009-C9RA07327B-s6160

RA-009-C9RA07327B-s6161

RA-009-C9RA07327B-s6162

RA-009-C9RA07327B-s6163

RA-009-C9RA07327B-s6164

RA-009-C9RA07327B-s6165

RA-009-C9RA07327B-s6166

RA-009-C9RA07327B-s6167

RA-009-C9RA07327B-s6168

RA-009-C9RA07327B-s6169

RA-009-C9RA07327B-s6170

RA-009-C9RA07327B-s6171

RA-009-C9RA07327B-s6172

RA-009-C9RA07327B-s6173

RA-009-C9RA07327B-s6174

RA-009-C9RA07327B-s6175

RA-009-C9RA07327B-s6176

RA-009-C9RA07327B-s6177

RA-009-C9RA07327B-s6178

RA-009-C9RA07327B-s6179

RA-009-C9RA07327B-s6180

RA-009-C9RA07327B-s6181

RA-009-C9RA07327B-s6182

RA-009-C9RA07327B-s6183

RA-009-C9RA07327B-s6184

RA-009-C9RA07327B-s6185

RA-009-C9RA07327B-s6186

RA-009-C9RA07327B-s6187

RA-009-C9RA07327B-s6188

RA-009-C9RA07327B-s6189

RA-009-C9RA07327B-s6190

RA-009-C9RA07327B-s6191

RA-009-C9RA07327B-s6192

RA-009-C9RA07327B-s6193

RA-009-C9RA07327B-s6194

RA-009-C9RA07327B-s6195

RA-009-C9RA07327B-s6196

RA-009-C9RA07327B-s6197

RA-009-C9RA07327B-s6198

RA-009-C9RA07327B-s6199

RA-009-C9RA07327B-s6200

RA-009-C9RA07327B-s6201

RA-009-C9RA07327B-s6202

RA-009-C9RA07327B-s6203

RA-009-C9RA07327B-s6204

RA-009-C9RA07327B-s6205

RA-009-C9RA07327B-s6206

RA-009-C9RA07327B-s6207

RA-009-C9RA07327B-s6208

RA-009-C9RA07327B-s6209

RA-009-C9RA07327B-s6210

RA-009-C9RA07327B-s6211

RA-009-C9RA07327B-s6212

RA-009-C9RA07327B-s6213

RA-009-C9RA07327B-s6214

RA-009-C9RA07327B-s6215

RA-009-C9RA07327B-s6216

RA-009-C9RA07327B-s6217

RA-009-C9RA07327B-s6218

RA-009-C9RA07327B-s6219

RA-009-C9RA07327B-s6220

RA-009-C9RA07327B-s6221

RA-009-C9RA07327B-s6222

RA-009-C9RA07327B-s6223

RA-009-C9RA07327B-s6224

RA-009-C9RA07327B-s6225

RA-009-C9RA07327B-s6226

RA-009-C9RA07327B-s6227

RA-009-C9RA07327B-s6228

RA-009-C9RA07327B-s6229

RA-009-C9RA07327B-s6230

RA-009-C9RA07327B-s6231

RA-009-C9RA07327B-s6232

RA-009-C9RA07327B-s6233

RA-009-C9RA07327B-s6234

RA-009-C9RA07327B-s6235

RA-009-C9RA07327B-s6236

RA-009-C9RA07327B-s6237

RA-009-C9RA07327B-s6238

RA-009-C9RA07327B-s6239

RA-009-C9RA07327B-s6240

RA-009-C9RA07327B-s6241

RA-009-C9RA07327B-s6242

RA-009-C9RA07327B-s6243

RA-009-C9RA07327B-s6244

RA-009-C9RA07327B-s6245

RA-009-C9RA07327B-s6246

RA-009-C9RA07327B-s6247

RA-009-C9RA07327B-s6248

RA-009-C9RA07327B-s6249

RA-009-C9RA07327B-s6250

RA-009-C9RA07327B-s6251

RA-009-C9RA07327B-s6252

RA-009-C9RA07327B-s6253

RA-009-C9RA07327B-s6254

RA-009-C9RA07327B-s6255

RA-009-C9RA07327B-s6256

RA-009-C9RA07327B-s6257

RA-009-C9RA07327B-s6258

RA-009-C9RA07327B-s6259

RA-009-C9RA07327B-s6260

RA-009-C9RA07327B-s6261

RA-009-C9RA07327B-s6262

RA-009-C9RA07327B-s6263

RA-009-C9RA07327B-s6264

RA-009-C9RA07327B-s6265

RA-009-C9RA07327B-s6266

RA-009-C9RA07327B-s6267

RA-009-C9RA07327B-s6268

RA-009-C9RA07327B-s6269

RA-009-C9RA07327B-s6270

RA-009-C9RA07327B-s6271

RA-009-C9RA07327B-s6272

RA-009-C9RA07327B-s6273

RA-009-C9RA07327B-s6274

RA-009-C9RA07327B-s6275

RA-009-C9RA07327B-s6276

RA-009-C9RA07327B-s6277

RA-009-C9RA07327B-s6278

RA-009-C9RA07327B-s6279

RA-009-C9RA07327B-s6280

RA-009-C9RA07327B-s6281

RA-009-C9RA07327B-s6282

RA-009-C9RA07327B-s6283

RA-009-C9RA07327B-s6284

RA-009-C9RA07327B-s6285

RA-009-C9RA07327B-s6286

RA-009-C9RA07327B-s6287

RA-009-C9RA07327B-s6288

RA-009-C9RA07327B-s6289

RA-009-C9RA07327B-s6290

RA-009-C9RA07327B-s6291

RA-009-C9RA07327B-s6292

RA-009-C9RA07327B-s6293

RA-009-C9RA07327B-s6294

RA-009-C9RA07327B-s6295

RA-009-C9RA07327B-s6296

RA-009-C9RA07327B-s6297

RA-009-C9RA07327B-s6298

RA-009-C9RA07327B-s6299

RA-009-C9RA07327B-s6300

RA-009-C9RA07327B-s6301

RA-009-C9RA07327B-s6302

RA-009-C9RA07327B-s6303

RA-009-C9RA07327B-s6304

RA-009-C9RA07327B-s6305

RA-009-C9RA07327B-s6306

RA-009-C9RA07327B-s6307

RA-009-C9RA07327B-s6308

RA-009-C9RA07327B-s6309

RA-009-C9RA07327B-s6310

RA-009-C9RA07327B-s6311

RA-009-C9RA07327B-s6312

RA-009-C9RA07327B-s6313

RA-009-C9RA07327B-s6314

RA-009-C9RA07327B-s6315

RA-009-C9RA07327B-s6316

RA-009-C9RA07327B-s6317

RA-009-C9RA07327B-s6318

RA-009-C9RA07327B-s6319

RA-009-C9RA07327B-s6320

RA-009-C9RA07327B-s6321

RA-009-C9RA07327B-s6322

RA-009-C9RA07327B-s6323

RA-009-C9RA07327B-s6324

RA-009-C9RA07327B-s6325

RA-009-C9RA07327B-s6326

RA-009-C9RA07327B-s6327

RA-009-C9RA07327B-s6328

RA-009-C9RA07327B-s6329

RA-009-C9RA07327B-s6330

RA-009-C9RA07327B-s6331

RA-009-C9RA07327B-s6332

RA-009-C9RA07327B-s6333

RA-009-C9RA07327B-s6334

RA-009-C9RA07327B-s6335

RA-009-C9RA07327B-s6336

RA-009-C9RA07327B-s6337

RA-009-C9RA07327B-s6338

RA-009-C9RA07327B-s6339

RA-009-C9RA07327B-s6340

RA-009-C9RA07327B-s6341

RA-009-C9RA07327B-s6342

RA-009-C9RA07327B-s6343

RA-009-C9RA07327B-s6344

RA-009-C9RA07327B-s6345

RA-009-C9RA07327B-s6346

RA-009-C9RA07327B-s6347

RA-009-C9RA07327B-s6348

RA-009-C9RA07327B-s6349

RA-009-C9RA07327B-s6350

RA-009-C9RA07327B-s6351

RA-009-C9RA07327B-s6352

RA-009-C9RA07327B-s6353

RA-009-C9RA07327B-s6354

RA-009-C9RA07327B-s6355

RA-009-C9RA07327B-s6356

RA-009-C9RA07327B-s6357

RA-009-C9RA07327B-s6358

RA-009-C9RA07327B-s6359

RA-009-C9RA07327B-s6360

RA-009-C9RA07327B-s6361

RA-009-C9RA07327B-s6362

RA-009-C9RA07327B-s6363

RA-009-C9RA07327B-s6364

RA-009-C9RA07327B-s6365

RA-009-C9RA07327B-s6366

RA-009-C9RA07327B-s6367

RA-009-C9RA07327B-s6368

RA-009-C9RA07327B-s6369

RA-009-C9RA07327B-s6370

RA-009-C9RA07327B-s6371

RA-009-C9RA07327B-s6372

RA-009-C9RA07327B-s6373

RA-009-C9RA07327B-s6374

RA-009-C9RA07327B-s6375

RA-009-C9RA07327B-s6376

RA-009-C9RA07327B-s6377

RA-009-C9RA07327B-s6378

RA-009-C9RA07327B-s6379

RA-009-C9RA07327B-s6380

RA-009-C9RA07327B-s6381

RA-009-C9RA07327B-s6382

RA-009-C9RA07327B-s6383

RA-009-C9RA07327B-s6384

## References

[cit1] Furukawa H., Cordova K. E., O'Keeffe M., Yaghi O. M. (2013). The chemistry and applications of metal-organic frameworks. Science.

[cit2] He Y. B., Chen F. L., Li B., Qian G. D., Zhou W., Chen B. L. (2018). Porous metal-organic frameworks for fuel storage. Coord. Chem. Rev..

[cit3] Lee J., Farha O. K., Roberts J., Scheidt K. A., Nguyen S. T., Hupp J. T. (2009). Metal-organic framework materials as catalysts. Chem. Soc. Rev..

[cit4] Sumida K., Rogow D. L., Mason J. A., McDonald T. M., Bloch E. D., Herm Z. R., Bae T. H., Long J. R. (2012). Carbon dioxide capture in metal-organic frameworks. Chem. Rev..

[cit5] Li J. R., Sculley J., Zhou H. C. (2012). Metal-organic frameworks for separations. Chem. Rev..

[cit6] Czaja A. U., Trukhan N., Muller U. (2009). Industrial applications of metal-organic frameworks. Chem. Soc. Rev..

[cit7] Chung Y. G., Camp J., Haranczyk M., Sikora B. J., Bury W., Krungleviciute V., Yildirim T., Farha O. K., Sholl D. S., Snurr R. Q. (2014). Computation-ready, experimental metal-organic frameworks: a tool to enable high-throughput screening of nanoporous crystals. Chem. Mater..

[cit8] Groom C. R., Bruno I. J., Lightfoot M. P., Ward S. C. (2016). The Cambridge structural database. Acta Crystallogr., Sect. B: Struct. Sci., Cryst. Eng. Mater..

[cit9] Woinska M., Grabowsky S., Dominiak P. M., Wozniak K., Jayatilaka D. (2016). Hydrogen atoms can be located accurately and precisely by X-ray crystallography. Sci. Adv..

[cit10] Hirshfeld F. L. (1976). Can X-ray data distinguish bonding effects from vibrational smearing?. Acta Crystallogr., Sect. A: Found. Crystallogr..

[cit11] Capelli S. C., Burgi H. B., Dittrich B., Grabowsky S., Jayatilaka D. (2014). Hirshfeld atom refinement. IUCrJ.

[cit12] Sturluson A., Hyynh M. T., Kaija A. R., Laird C., Sunghyun Y., Hou F., Feng Z., Wilmer C. E., Colon Y. J., Chung Y. G., Siderius D. W., Simon C. M. (2019). The role of molecular modelling and simulation in the discovery and deployment of metal-organic frameworks for gas storage and separation. Mol. Simul..

[cit13] Barthel S., Alexandrov E. V., Proserpio D. M., Smit B. (2018). Distinguising metal-organic frameworks. Cryst. Growth Des..

[cit14] Ding N., Armatas G. S., Kanatzidis M. G. (2010). Metal inorganic frameworks: dynamic flexible architecture with extended pore order built from [Se_3_]^2−^ linkers and [Re_6_Se_6_Br_8_]^2−^ clusters. J. Am. Chem. Soc..

[cit15] Kumar S., Samolia M., Kumar T. J. D. (2018). Hydrogen storage in Sc and Li decorated metal–inorganic framework. ACS Appl. Energy Mater..

[cit16] Nazarian D., Camp J. S., Sholl D. S. (2016). A comprehensive set of high-quality point charges for simulations of metal-organic frameworks. Chem. Mater..

[cit17] Nazarian D., Camp J. S., Chung Y. G., Snurr R. Q., Sholl D. S. (2017). Large-scale refinement of metal-organic framework structures using density functional theory. Chem. Mater..

[cit18] Wilmer C. E., Leaf M., Lee C. Y., Farha O. K., Hauser B. G., Hupp J. T., Snurr R. Q. (2012). Large-scale screening of hypothetical metal-organic frameworks. Nat. Chem..

[cit19] Sikora B. J., Wilmer C. E., Greenfield M. L., Snurr R. Q. (2012). Thermodynamic analysis of Xe/Kr selectivity in over 137000 hypothetical metal-organic frameworks. Chem. Sci..

[cit20] Cui Y. J., Yue Y. F., Qian G. D., Chen B. L. (2012). Luminescent functional metal-organic frameworks. Chem. Rev..

[cit21] Yang Q. Y., Liu D. H., Zhong C. L., Li J. R. (2013). Development of computational methodologies for metal-organic frameworks and their application in gas separations. Chem. Rev..

[cit22] Haldoupis E., Borycz J., Shi H. L., Vogiatzis K. D., Bai P., Queen W. L., Gagliardi L., Siepmann J. I. (2015). *Ab initio* derived force fields for predicting CO_2_ adsorption and accessibility of metal sites in the metal-organic frameworks M-MOF-74 (M = Mn, Co, Ni, Cu). J. Phys. Chem. C.

[cit23] Lamia N., Jorge M., Granato M. A., Paz F. A. A., Chevreau H., Rodrigues A. E. (2009). Adsorption of propane, propylene and isobutane on a metal-organic framework: molecular simulation and experiment. Chem. Eng. Sci..

[cit24] Skoulidas A. I., Sholl D. S. (2005). Self-diffusion and transport diffusion of light gases in metal-organic framework materials assessed using molecular dynamics simulations. J. Phys. Chem. B.

[cit25] Keskin S., Liu J. C., Johnson J. K., Sholl D. S. (2008). Testing the accuracy of correlations for multicomponent mass transport of adsorbed gases in metal-organic frameworks: diffusion of H_2_/CH_4_ mixtures in CuBTC. Langmuir.

[cit26] Grimme S., Hansen A., Brandenburg J. G., Bannwarth C. (2016). Dispersion-corrected mean-field electronic structure methods. Chem. Rev..

[cit27] Hermann J., DiStasio R., Tkatchenko A. (2017). First-principles models for van der waals interactions in molecules and materials: concepts, theory, and applications. Chem. Rev..

[cit28] Kaplan I. G. , Intermolecular Interactions: Physical Picture, Computational Methods and Model Potentials, John Wiley & Sons, West Sussex, England, 2006

[cit29] Wang J. M., Wolf R. M., Caldwell J. W., Kollman P. A., Case D. A. (2004). Development and testing of a general amber force field. J. Comput. Chem..

[cit30] Gates T. S., Odegard G. M., Frankland S. J. V., Clancy T. C. (2005). Computational materials: multi-scale modeling and simulation of nanostructured materials. Compos. Sci. Technol..

[cit31] Murtola T., Bunker A., Vattulainen I., Deserno M., Karttunen M. (2009). Multiscale modeling of emergent materials: biological and soft matter. Phys. Chem. Chem. Phys..

[cit32] Bush B. L., Sheridan R. P. (1993). PATTY - a programmable atom typer and language for automatic classification of atoms in molecular databases. J. Chem. Inf. Comput. Sci..

[cit33] Bristow J. K., Tiana D., Walsh A. (2014). Transferable force field for metal-organic frameworks from first-principles: BTW-FF. J. Chem. Theory Comput..

[cit34] Vanduyfhuys L., Verstraelen T., Vandichel M., Waroquier M., Van Speybroeck V. (2012). *Ab initio* parametrized force field for the flexible metal-organic framework MIL-53(Al). J. Chem. Theory Comput..

[cit35] Vanduyfhuys L., Vandenbrande S., Verstraelen T., Schmid R., Waroquier M., Van Speybroeck V. (2015). QuickFF: a program for a quick and easy derivation of force fields for metal-organic frameworks from *ab initio* input. J. Comput. Chem..

[cit36] Dubbeldam D., Walton K. S., Ellis D. E., Snurr R. Q. (2007). Exceptional negative thermal expansion in isoreticular metal-organic frameworks. Angew. Chem., Int. Ed..

[cit37] Heinen J., Dubbeldam D. (2018). On flexible force fields for metal-organic frameworks: recent developments and future prospects. Wiley Interdiscip. Rev.: Comput. Mol. Sci..

[cit38] Bureekaew S., Amirjalayer S., Tafipolsky M., Spickermann C., Roy T. K., Schmid R. (2013). MOF-FF: a flexible first-principles derived force field for metal-organic frameworks. Phys. Status Solidi B.

[cit39] Ghahremanpour M. M., van Maaren P. J., Caleman C., Hutchison G. R., van der Spoel D. (2018). Polarizable Drude model with s-type Gaussian or Slater charge density for general molecular mechanics force fields. J. Chem. Theory Comput..

[cit40] Visscher K. M., Geerke D. P. (2019). Deriving force-field parameters from first principles using a polarizable and higher order dispersion model. J. Chem. Theory Comput..

[cit41] Lemkul J. A., Huang J., Roux B., MacKerell A. D. (2016). An empirical polarizable force field based on the classical Drude oscillator model: development history and recent applications. Chem. Rev..

[cit42] Ponder J. W., Wu C. J., Ren P. Y., Pande V. S., Chodera J. D., Schnieders M. J., Haque I., Mobley D. L., Lambrecht D. S., DiStasio R. A., Head-Gordon M., Clark G. N. I., Johnson M. E., Head-Gordon T. (2010). Current status of the AMOEBA polarizable force field. J. Phys. Chem. B.

[cit43] Halgren T. A., Damm W. (2001). Polarizable force fields. Curr. Opin. Struct. Biol..

[cit44] Van Vleet M. J., Misquitta A. J., Stone A. J., Schmidtt J. R. (2016). Beyond Born-Mayer: improved models for short-range repulsion in *ab initio* force fields. J. Chem. Theory Comput..

[cit45] Vandenbrande S., Waroquier M., Van Speybroeck V., Verstraelen T. (2017). The monomer electron density force field (MEDFF): a physically inspired model for noncovalent interactions. J. Chem. Theory Comput..

[cit46] Wang L. P., Chen J. H., Van Voorhis T. (2013). Systematic parametrization of polarizable force fields from quantum chemistry data. J. Chem. Theory Comput..

[cit47] Waldher B., Kuta J., Chen S., Henson N., Clark A. E. (2010). ForceFit: a code to fit classical force fields to quantum mechanical potential energy surfaces. J. Comput. Chem..

[cit48] Desgranges C., Delhommelle J. (2015). Many-body effects on the thermodynamics of fluids, mixtures, and nanoconfined fluids. J. Chem. Theory Comput..

[cit49] McDaniel J. G., Schmidt J. R. (2014). First-principles many-body force fields from the gas phase to liquid: a “universal” approach. J. Phys. Chem. B.

[cit50] Kiss P., Baranyai A. (2012). Testing the recent charge-on-spring type polarizable water models. II Vapor-liquid equilibrium. J. Chem. Phys..

[cit51] Allen C. A., Boissonnault J. A., Cirera J., Gulland R., Paesani F., Cohen S. M. (2013). Chemically crosslinked isoreticular metal-organic frameworks. Chem. Commun..

[cit52] Car R., Parrinello M. (1985). Unified approach for molecular dynamics and density-functional theory. Phys. Rev. Lett..

[cit53] Laasonen K., Pasquarello A., Car R., Lee C., Vanderbilt D. (1993). Car-Parrinello molecular dynamics with Vanderbilt ultrasoft pseudopotentials. Phys. Rev. B: Condens. Matter Mater. Phys..

[cit54] Kresse G., Hafner J. (1993). Abinitio molecular-dynamics for liquid-metals. Phys. Rev. B: Condens. Matter Mater. Phys..

[cit55] Kresse G., Hafner J. (1994). Ab-initio molecular-dynamics simulation of the liquid-metal amorphous-semiconductor transition in germanium. Phys. Rev. B: Condens. Matter Mater. Phys..

[cit56] Horton J. T., Allen A. E. A., Dodda L. S., Cole D. J. (2019). QUBEKit: automating the derivation of force field parameters from quantum mechanics. J. Chem. Inf. Model..

[cit57] Xu P., Guidez E. B., Bertoni C., Gordon M. S. (2018). Perspective: *ab initio* force field methods derived from quantum mechanics. J. Chem. Phys..

[cit58] Chen B., Siepmann J. I. (1999). Transferable potentials for phase equilibria. 3. Explicit-hydrogen description of normal alkanes. J. Phys. Chem. B.

[cit59] Chmiela S., Tkatchenko A., Sauceda H. E., Poltavsky I., Schutt K. T., Muller K. R. (2017). Machine learning of accurate energy-conserving molecular force fields. Sci. Adv..

[cit60] Maxwell P., di Pasquale N., Cardamone S., Popelier P. L. A. (2016). The prediction of topologically partitioned intra-atomic and inter-atomic energies by the machine learning method kriging. Theor. Chem. Acc..

[cit61] Botu V., Batra R., Chapman J., Ramprasad R. (2017). Machine learning force fields: construction, validation, and outlook. J. Phys. Chem. C.

[cit62] Bleiziffer P., Schaller K., Riniker S. (2018). Machine learning of partial charges derived from high-quality quantum-mechanical calculations. J. Chem. Inf. Model..

[cit63] Perdew J. P., Burke K., Ernzerhof M. (1996). Generalized gradient approximation made simple. Phys. Rev. Lett..

[cit64] Kresse G., Furthmuller J. (1996). Efficient iterative schemes for *ab initio* total-energy calculations using a plane-wave basis set. Phys. Rev. B: Condens. Matter Mater. Phys..

[cit65] Kresse G., Furthmuller J. (1996). Efficiency of *ab initio* total energy calculations for metals and semiconductors using a plane-wave basis set. Comput. Mater. Sci..

[cit66] Kresse G., Joubert D. (1999). From ultrasoft pseudopotentials to the projector augmented-wave method. Phys. Rev. B: Condens. Matter Mater. Phys..

[cit67] Blochl P. E. (1994). Projector augmented-wave method. Phys. Rev. B: Condens. Matter Mater. Phys..

[cit68] Hafner J. (2008). Ab-initio simulations of materials using VASP: density-functional theory and beyond. J. Comput. Chem..

[cit69] Gabaldon Limas N., Manz T. A. (2018). Introducing DDEC6 atomic population analysis: part 4. Efficient parallel computation of net atomic charges, atomic spin moments, bond orders, and more. RSC Adv..

[cit70] Monkhorst H. J., Pack J. D. (1976). Special points for Brillouin-zone integrations. Phys. Rev. B: Condens. Matter Mater. Phys..

[cit71] Frisch M. J. , Trucks G. W., Schlegel H. B., Scuseria G. E., Robb M. A., Cheeseman J. R., Scalmani G., Barone V., Petersson G. A., Nakatsuji H., Li X., Caricato M., Marenich A. V., Bloino J., Janesko B. G., Gomperts R., Mennucci B., Hratchian H. P., Ortiz J. V., Izmaylov A. F., Sonnenberg J. L., Williams-Young D., Ding F., Lipparini F., Egidi F., Goings J., Peng B., Petrone A., Henderson T., Ranasinghe D., Zakrzewski V. G., Gao J., Rega N., Zheng G., Liang W., Hada M., Ehara M., Toyota K., Fukuda R., Hasegawa J., Ishida M., Nakajima T., Honda Y., Kitao O., Nakai H., Vreven T., Throssell K., Montgomery Jr J. A., Peralta J. E., Ogliaro F., Bearpark M. J., Heyd J. J., Brothers E. N., Kudin K. N., Staroverov V. N., Keith T. A., Kobayashi R., Normand J., Raghavachari K., Rendell A. P., Burant J. C., Iyengar S. S., Tomasi J., Cossi M., Millam J. M., Klene M., Adamo C., Cammi R., Ochterski J. W., Martin R. L., Morokuma K., Farkas O., Foresman J. B. and Fox D. J., Gaussian 16, Revision B.01, Gaussian, Inc., Wallingford CT, 2016

[cit72] Becke A. D. (1993). Density-functional thermochemistry 3. The role of exact exchange. J. Chem. Phys..

[cit73] Stephens P. J., Devlin F. J., Chabalowski C. F., Frisch M. J. (1994). Ab-initio calculation of vibrational absorption and circular-dichroism spectra using density-functional force-fields. J. Phys. Chem..

[cit74] Manz T. A., Chen T., Cole D. J., Gabaldon Limas N., Fiszbein B. (2019). New scaling relations to compute atom-in-material polarizabilities and dispersion coefficients: part 1. Theory and accuracy. RSC Adv..

[cit75] Rappoport D., Furche F. (2010). Property-optimized Gaussian basis sets for molecular response calculations. J. Chem. Phys..

[cit76] Manz T. A., Gabaldon Limas N. (2016). Introducing DDEC6 atomic population analysis: part 1. Charge partitioning theory and methodology. RSC Adv..

[cit77] Manz T. A., Sholl D. S. (2011). Methods for computing accurate atomic spin moments for collinear and noncollinear magnetism in periodic and nonperiodic materials. J. Chem. Theory Comput..

[cit78] Lillestolen T. C., Wheatley R. J. (2008). Redefining the atom: atomic charge densities produced by an iterative stockholder approach. Chem. Commun..

[cit79] Tang K. T., Toennies J. P. (1984). An improved simple-model for the van der Waals potential based on universal damping functions for the dispersion coefficients. J. Chem. Phys..

[cit80] Tang K. T., Toennies J. P. (1992). The damping function of the van der Waals attraction in the potential between rare-gas atoms and metal surfaces. Surf. Sci..

[cit81] Manz T. A., Chen T. (2019). New scaling relations to compute atom-in-material polarizabilities and dispersion coefficients: part 2. Linear-scaling computational algorithms and parallelization. RSC Adv..

[cit82] Starkschall G., Gordon R. G. (1972). Calculation of coefficients in the power series expansion of the long-range dispersion force between atoms. J. Chem. Phys..

[cit83] Axilrod B. M., Teller E. (1943). Interaction of the van der Waals type between three atoms. J. Chem. Phys..

[cit84] Walters E. T., Mohebifar M., Johnson E. R., Rowley C. N. (2018). Evaluating the London dispersion coefficients of protein force fields using the exchange-hole dipole moment model. J. Phys. Chem. B.

[cit85] Jones A. P., Crain J., Sokhan V. P., Whitfield T. W., Martyna G. J. (2013). Quantum Drude oscillator model of atoms and molecules: many-body polarization and dispersion interactions for atomistic simulation. Phys. Rev. B: Condens. Matter Mater. Phys..

[cit86] Whitfield T. W., Martyna G. J. (2007). Low variance energy estimators for systems of quantum Drude oscillators: treating harmonic path integrals with large separations of time scales. J. Chem. Phys..

[cit87] Sadhukhan M., Manby F. R. (2016). Quantum mechanics of Drude oscillators with full Coulomb interaction. Phys. Rev. B.

[cit88] Manz T. A., Sholl D. S. (2012). Improved atoms-in-molecule charge partitioning functional for simultaneously reproducing the electrostatic potential and chemical states in periodic and nonperiodic materials. J. Chem. Theory Comput..

[cit89] Campana C., Mussard B., Woo T. K. (2009). Electrostatic potential derived atomic charges for periodic systems using a modified error functional. J. Chem. Theory Comput..

[cit90] Cox S. R., Williams D. E. (1981). Representation of the molecular electrostatic potential by a net atomic charge model. J. Comput. Chem..

[cit91] Watanabe T., Manz T. A., Sholl D. S. (2011). Accurate treatment of electrostatics during molecular adsorption in nanoporous crystals without assigning point charges to framework atoms. J. Phys. Chem. C.

[cit92] Gabaldon Limas N., Manz T. A. (2016). Introducing DDEC6 atomic population analysis: part 2. Computed results for a wide range of periodic and nonperiodic materials. RSC Adv..

[cit93] Bayly C. I., Cieplak P., Cornell W. D., Kollman P. A. (1993). A well-behaved electrostatic potential based method using charge restraints for deriving atomic charges - the RESP model. J. Phys. Chem..

[cit94] Manz T. A. (2017). Introducing DDEC6 atomic population analysis: part 3. Comprehensive method to compute bond orders. RSC Adv..

[cit95] Hanson R. M. (2010). Jmol - a paradigm shift in crystallographic visualization. J. Appl. Crystallogr..

[cit96] Jmol: an open-source Java viewer for chemical structures in 3D, http://www.jmol.org, accessed August 2019

[cit97] Verstraelen T., Vandenbrande S., Heidar-Zadeh F., Vanduyfhuys L., Van Speybroeck V., Waroquier M., Ayers P. W. (2016). Minimal basis iterative stockholder: atoms in molecules for force-field development. J. Chem. Theory Comput..

[cit98] Wang B., Truhlar D. G. (2010). Including charge penetration effects in molecular modeling. J. Chem. Theory Comput..

[cit99] Freitag M. A., Gordon M. S., Jensen J. H., Stevens W. J. (2000). Evaluation of charge penetration between distributed multipolar expansions. J. Chem. Phys..

[cit100] Vanommeslaeghe K., MacKerell A. D. (2012). Automation of the CHARMM general force field (CGenFF) I: bond perception and atom typing. J. Chem. Inf. Model..

[cit101] Kaminski G. A., Friesner R. A., Tirado-Rives J., Jorgensen W. L. (2001). Evaluation and reparametrization of the OPLS-AA force field for proteins via comparison with accurate quantum chemical calculations on peptides. J. Phys. Chem. B.

[cit102] Klauda J. B., Venable R. M., Freites J. A., O'Connor J. W., Tobias D. J., Mondragon-Ramirez C., Vorobyov I., MacKerell A. D., Pastor R. W. (2010). Update of the CHARMM all-atom additive force field for lipids: validation on six lipid types. J. Phys. Chem. B.

[cit103] Dickson C. J., Madej B. D., Skjevik A. A., Betz R. M., Teigen K., Gould I. R., Walker R. C. (2014). Lipid14: the Amber lipid force field. J. Chem. Theory Comput..

[cit104] Hobza P., Kabelac M., Sponer J., Mejzlik P., Vondrasek J. (1997). Performance of empirical potentials (AMBER, CFF95, CVFF, CHARMM, OPLS, POLTEV), semiempirical quantum chemical methods (AM1, MNDO/M, PM3), and *ab initio* Hartree-Fock method for interaction of DNA bases: comparison with nonempirical beyond Hartree-Fock results. J. Comput. Chem..

[cit105] Brooks B. R., Brooks C. L., Mackerell A. D., Nilsson L., Petrella R. J., Roux B., Won Y., Archontis G., Bartels C., Boresch S., Caflisch A., Caves L., Cui Q., Dinner A. R., Feig M., Fischer S., Gao J., Hodoscek M., Im W., Kuczera K., Lazaridis T., Ma J., Ovchinnikov V., Paci E., Pastor R. W., Post C. B., Pu J. Z., Schaefer M., Tidor B., Venable R. M., Woodcock H. L., Wu X., Yang W., York D. M., Karplus M. (2009). CHARMM: the biomolecular simulation program. J. Comput. Chem..

[cit106] Salomon-Ferrer R., Case D. A., Walker R. C. (2013). An overview of the Amber biomolecular simulation package. Wiley Interdiscip. Rev.: Comput. Mol. Sci..

[cit107] Rappe A. K., Casewit C. J., Colwell K. S., Goddard W. A., Skiff W. M. (1992). UFF: a full periodic-table force-field for molecular mechanics and molecular-dynamics simulations. J. Am. Chem. Soc..

[cit108] Coupry D. E., Addicoat M. A., Heine T. (2016). Extension of the universal force field for metal-organic frameworks. J. Chem. Theory Comput..

[cit109] Addicoat M. A., Vankova N., Akter I. F., Heine T. (2014). Extension of the universal force field to metal-organic frameworks. J. Chem. Theory Comput..

[cit110] Xu Q., Zhong C. L. (2010). A general approach for estimating framework charges in metal-organic frameworks. J. Phys. Chem. C.

[cit111] Zheng C. C., Zhong C. L. (2010). Estimation of framework charges in covalent organic frameworks using connectivity-based atom contribution method. J. Phys. Chem. C.

[cit112] Katritzky A. R., Topsom R. D. (1971). The sigma- and pi-inductive effects. J. Chem. Educ..

[cit113] Smith L. O. and Cristol S. J., Organic Chemistry, Reinhold Publishing Corporation, New York, 1966, p. 212

[cit114] Towns J., Cockerill T., Dahan M., Foster I., Gaither K., Grimshaw A., Hazlewood V., Lathrop S., Lifka D., Peterson G. D., Roskies R., Scott J. R., Wilkins-Diehr N. (2014). XSEDE: accelerating scientific discovery. Comput. Sci. Eng..

[cit115] Altintas C., Avci G., Daglar H., Azar A. N. V., Erucar I., Velioglu S., Keskin S. (2019). An extensive comparative analysis of two MOF databases: high-throughput screening of computation-ready MOFs for CH_4_ and H_2_ adsorption. J. Mater. Chem. A.

[cit116] Moghadam P. Z., Li A., Wiggin S. B., Tao A., Maloney A. G. P., Wood P. A., Ward S. C., Fairen-Jimenez D. (2017). Development of a Cambridge Structural Database subset: a collection of metal-organic frameworks for past, present, and future. Chem. Mater..

[cit117] Altintas C., Avci G., Daglar H., Azar A. N. V., Velioglu S., Erucar I., Keskin S. (2018). Database for CO_2_ separation performances of MOFs based on computational materials screening. ACS Appl. Mater. Interfaces.

